# Khasianine Affects the Expression of Sugar-Sensitive Proteins in Pancreatic Cancer Cells, Which Are Altered in Data from the Rat Model and Patients

**DOI:** 10.1021/acsptsci.3c00013

**Published:** 2023-04-13

**Authors:** Micah N. Sagini, Karel D. Klika, Robert W. Owen, Martin R. Berger

**Affiliations:** † Toxicology and Chemotherapy Unit, German Cancer Research Center (DKFZ), Im Neuenheimer Feld 580, 69120 Heidelberg, Germany; ‡ Molecular Structure Analysis, German Cancer Research Center (DKFZ), Im Neuenheimer Feld 280, 69120 Heidelberg, Germany; § Biochemistry and Biomarkers Unit, German Cancer Research Center (DKFZ), Im Neuenheimer Feld 580, 69120 Heidelberg, Germany

**Keywords:** PDAC, affinity chromatography, khasianine, solasodine glycosides, LSBPs

## Abstract

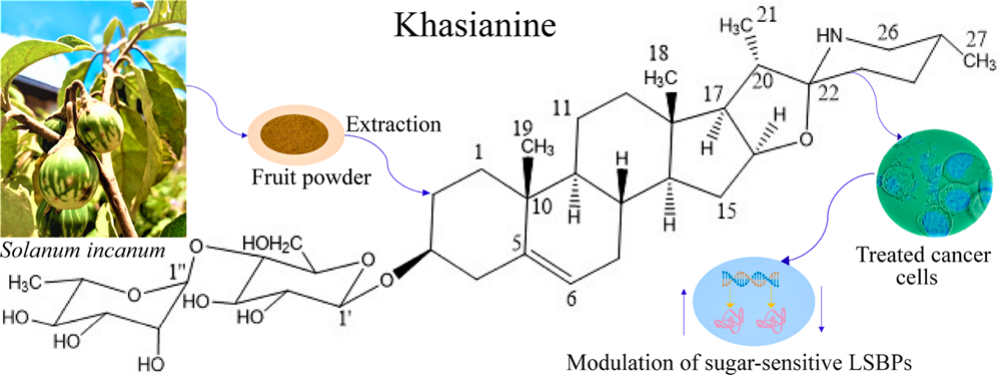

Pancreatic ductal adenocarcinoma (PDAC) is a deadly malignancy with no effective treatment, particularly in the advanced stage. This study explored the antiproliferative activity of khasianine against pancreatic cancer cell lines of human (Suit2-007) and rat (ASML) origin. Khasianine was purified from *Solanum incanum* fruits by silica gel column chromatography and analyzed by LC-MS and NMR spectroscopy. Its effect in pancreatic cancer cells was evaluated by cell proliferation assay, chip array and mass spectrometry. Proteins showing sensitivity to sugars, i.e. sugar-sensitive lactosyl-Sepharose binding proteins (LSBPs), were isolated from Suit2-007 cells by competitive affinity chromatography. The eluted fractions included galactose-, glucose-, rhamnose- and lactose-sensitive LSBPs. The resulting data were analyzed by Chipster, Ingenuity Pathway Analysis (IPA) and GraphPad Prism. Khasianine inhibited proliferation of Suit2-007 and ASML cells with IC_50_ values of 50 and 54 μg/mL, respectively. By comparative analysis, khasianine downregulated lactose-sensitive LSBPs the most (126%) and glucose-sensitive LSBPs the least (85%). Rhamnose-sensitive LSBPs overlapped significantly with lactose-sensitive LSBPs and were the most upregulated in data from patients (23%) and a pancreatic cancer rat model (11.5%). From IPA, the Ras homolog family member A (RhoA) emerged as one of the most activated signaling pathways involving rhamnose-sensitive LSBPs. Khasianine altered the mRNA expression of sugar-sensitive LSBPs, some of which were modulated in data from patients and the rat model. The antiproliferative effect of khasianine in pancreatic cancer cells and the downregulation of rhamnose-sensitive proteins underscore the potential of khasianine in treating pancreatic cancer.

Pancreatic ductal adenocarcinoma (PDAC) is a lethal malignancy and the fourth most frequent cause of cancer-related deaths worldwide.^1^ Treatment modalities for advanced PDAC are limited due to a number of factors, including hardly detectable early metastasis and inherent heterogeneity.^2,3^ The lack of reliable diagnostic markers precludes early detection of pancreatic cancer due to the absence of clinical signs in patients.^4^ Heterogeneous tumors also complicate therapy by conferring a distinct tumor behavior in response to therapy, resulting in variable clinical outcomes.^5^ PDAC has a 5-year survival rate of 5–10% and is projected to be the second-ranked cause of cancer-related mortality by 2030.^6^ If diagnosed early, surgical intervention can prolong survival for eligible patients.^7^ Gemcitabine plus nab-paclitaxel or a combination of folinic acid, 5-fluorouracil, irinotecan and oxaliplatin (FOLFIRINOX) is recommended for treating PDAC.^8,9^ These regimens, however, confer only marginal benefits to patients and are often associated with side effects.^10^ Therefore, novel drugs with less or, preferably, no side effects, which can prolong patient survival, are urgently needed.

In our previous study, we demonstrated that PDAC cell lines (Suit2-007 and ASML) express lactosyl-Sepharose binding proteins (LSBPs), which were shown to bind simple sugars, including galactose (Gal), glucose (Glc), fucose (Fuc), mannose (Man) and rhamnose (Rha).^11−13^ It was also evident that only a subgroup of these proteins could bind these sugars. As some of these proteins were significantly expressed in a rat model for liver metastasis and in data from pancreatic cancer patients, it was anticipated that this property (sugar binding) could offer a rationale for evaluating the biological activity of sugar-bearing compounds against PDAC. In this context, it was presumed that solasodine glycosides such as solamargine and solasonine di- and monoglycosides could induce significant changes in LSBP expression.

Solasodine glycosides are secondary metabolites with anticancer properties and are commonly present in plants belonging to the Solanaceae family. Structurally, they are composed of a steroid nucleus linked to a carbohydrate side chain consisting of one or more sugar moieties.^14,15^ These compounds have been investigated for antiproliferative activities in various cancer cell lines, including colon, breast, human hepatoma, lung and gastric cancers.^16,17^ Solasodine glycosides are believed to target tumor cells by binding to endogenous endocytic lectins (EELs) expressed on the cell surface. In the cell, they inhibit proliferation by triggering apoptosis through intrinsic and extrinsic pathways. Other studies have shown that these compounds could target genes associated with the progression of malignancies, including PDAC.^18,19^ In squamous cell carcinoma, for instance, an extract prepared from *Solanum incanum*, with solamargine as the main component, induced apoptosis by upregulating tumor necrosis factor receptors and Fas. This extract also modulated the mitochondrial apoptotic pathway by upregulating cytochrome c and Bax and downregulating Bcl-X (L).^20^ In HER2-positive breast cancer, solamargine downregulated HER2/neu receptor, which is associated with growth and progression of this malignancy.^21^ In lung cancer, solamargine inhibited cancer cell lines by downregulating prostaglandin E2, DNA methyltransferase 1 (DNMT1) and c-Jun.^22^

The biological activities of solasodine glycosides are attributed to the presence and number of Rha in the carbohydrate moiety.^23,24^ For instance, the activity of solamargine, a three-sugar compound with two Rha’s, is higher than that of khasianine (*O*-α-l-rhamnopyranosyl-(1→4_glc_)-*O*-3-β-d-glucopyranosyl solasodine), which has only two sugars (Rha and Glc).^23^

In the present study, we investigated the anticancer effects of khasianine against pancreatic cancer cells containing LSBPs. In particular, we wanted to quantify the LSBP subgroups showing sensitivity to sugars in cancer cell lines as demonstrated elsewhere.^12^ To achieve these aims, we first isolated khasianine from *S. incanum*, a plant known to contain large amounts of solasodine glycosides. We then quantified sugar-sensitive LSBPs in various cancer cells and evaluated the antiproliferative activity of khasianine against two pancreatic cancer cell lines. To clarify the application of khasianine as a possible treatment for pancreatic cancer, we examined its effect on sugar-sensitive LBSPs and compared these findings with their respective gene expression in a chip array derived from animal models and patients.

## Results

### Isolation of Khasianine by Chromatography

An extract from *S. incanum* obtained using 70% ethanol was further extracted with *n*-butanol, resulting in two phases: an upper yellowish phase and a lower dark-brown phase. When we tested these extracts against human PDAC Suit2-007 cells, the upper *n*-butanol phase showed greater activity than the lower aqueous phase (results not shown). The *n*-butanol extract was further purified by silica gel column chromatography, and khasianine was detected in fractions eluted with 20, 30 and 50% methanol in DCM (Figure S2A–C).

### Confirmation of the Identity of the Purified Compound

**Figure 1:**
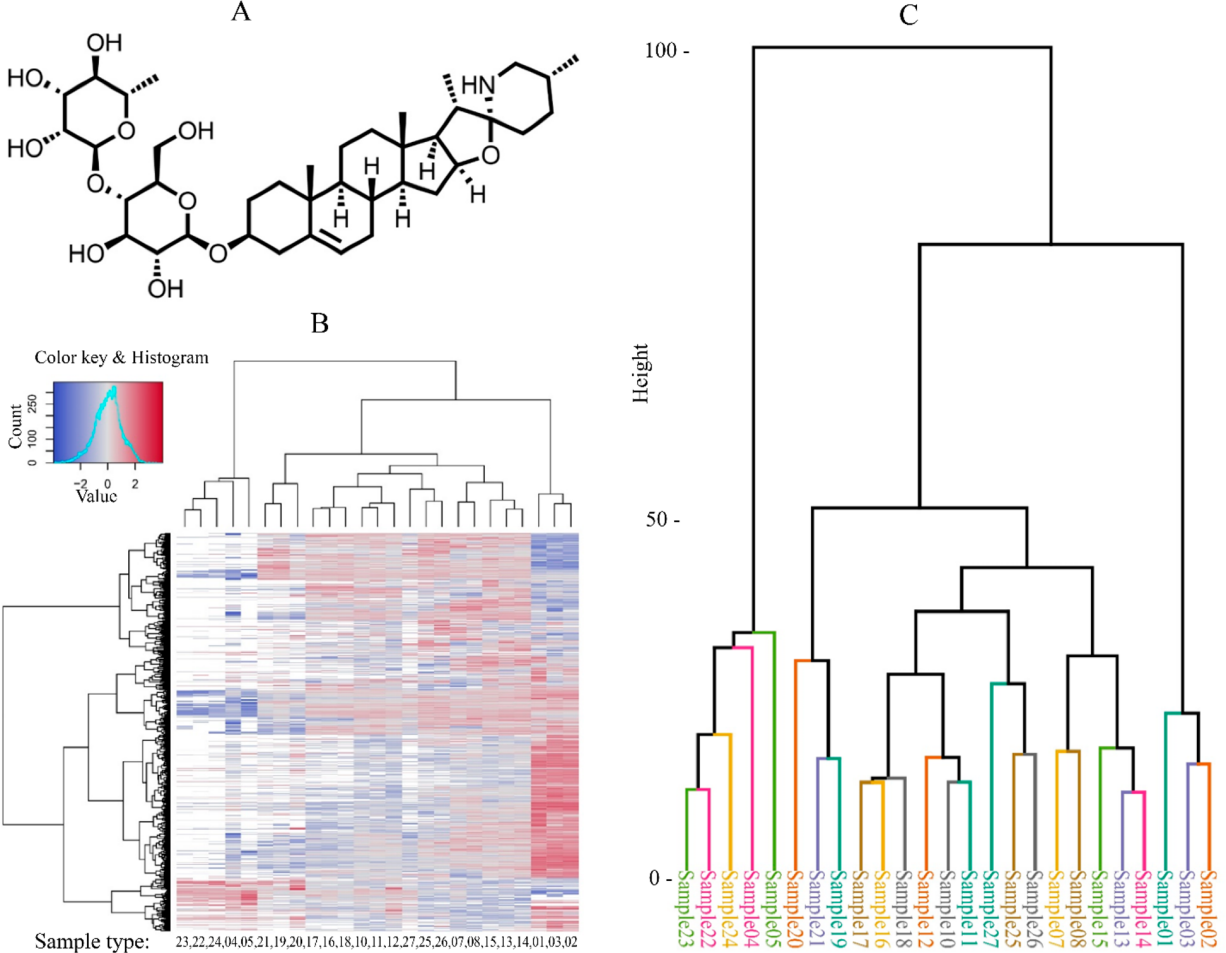
**Isolation of khasianine and sugar-sensitive LSBPs.** A and B represent the structure of khasianine and the resulting heatmap for LSBP fractions as analyzed by MS. Fractions were isolated from Suit2-007 cells by competitive affinity chromatography. Samples analyzed include control LSBP (1–3), Lac binding (4 and 5), non-Lac binding (7 and 8), Gal binding (10–12), non-Gal binding (13–15), Glc binding (16–18), non-Glc binding (19–21), Rha binding (22–24) and non-Rha binding (25–27). The dendrogram in C depicts a hierarchical clustering for all LSBPs fractions.

We confirmed the isolated compound as khasianine (Figure 1A) by NMR as depicted in the ^1^H and ^13^C NMR spectra (Figures S4–S15) and the NMR data (Tables S1 and S2). The ^13^C NMR data of khasianine was in agreement with the published data,^25^ though there were some notable deviations for a few nuclei near the top end of the steroid nucleus. These deviations were presumed to be due to partial protonation of the nitrogen atom that induces δ changes either directly through electronic effects or indirectly by way of conformational changes. In addition, some signals were also clearly exchange-broadened due to a dynamic process. However, the ^13^C δ’s reported by Mahato et al.^25^ and the ^13^C δ’s measured for a commercial sample of khasianine in *d*_5_-pyridine matched extremely well after confirming the assumption that the isolated khasianine was partially protonated by the addition of excess DCl to the commercial khasianine sample. As a result of protonation, for the ^13^C signals with significant deviations between the values for the isolated khasianine and those in the literature, those signals in the commercial sample (khasianine) moved either upfield or downfield according to their respective dispositions between the commercial khasianine sample before the addition of DCl and the partially protonated sample of purified khasianine (Table S1). Likewise, in the ^1^H NMR spectra, the δ’s for the signals of the isolated khasianine sample were intermediate between the δ’s for the signals of the commercial khasianine sample and the protonated commercial khasianine sample when there was a significant disparity in the δ’s for the latter two samples (Table S2). Signal assignments and sugar residue connections for the isolated khasianine sample were determined by the standard application and interpretation of 2D COSY, HSQC and HMBC NMR spectra (Figures S16–S30).

### Sensitivity of LSBP Subgroups from Suit2-007 Cells to Different Sugars

By competitive affinity chromatography we isolated and identified protein subgroups from LSBPs that were initially extracted from Suit2-007 cell lysates. During separation, these proteins could not bind to the lactosyl resin due to the presence of sugars (Gal, Glc, Rha and Lac) in the mobile phase. The protein subgroups resulting from this separation were accordingly named as Gal-sensitive, Glc-sensitive, Rha-sensitive and Lac-sensitive LSBPs. A heatmap analysis of MS data is depicted in Figure 1B, which shows the LSBP fractions eluted with respective loading buffers containing the particular sugars. Also shown are those LSBPs retained in the column but subsequently eluted with a high salt concentration buffer. Protein fractions analyzed by MS include controls, Gal-/non-Gal-sensitive, Glc-/non-Glc-sensitive, Rha-/non-Rha-sensitive and Lac-/non-Lac-sensitive LSBPs (Figure 1B). The chromatograms for these proteins are depicted in Figure S31A–F, and the identities of sugar sensitive-LSBPs are given in Tables S3–S6.

To determine the presence of sugar-sensitive LSBPs in other cancer cell lines, we used the results of the competitive affinity experiment for Suit2-007 cell line as the basis for further investigation. The additional cell lines investigated included ASML and BXPC3 (PDAC), MCF7 (breast cancer) and LST (colorectal cancer). We first isolated LSBPs from these cell lines by affinity chromatography^13^ and extracted the respective protein IDs for each cell line by matching with those of Suit2-007 cells characterized by MS. The total number of LSBPs per cell line and the sugar-sensitive LSBPs matching with those found in the Suit2-007 cells are shown in Table 1.

**Table 1:** Quantification of Sugar-Sensitive LSBPs in Five Cancer Cell Lines

	Suit2-007, *n* (%)^a^	ASML, *n* (%)^a^	BXPC3, *n* (%)^a^	MCF7, *n* (%)^a^	LST, *n* (%)^a^
Total LSBPs^b^	1593	1955	1208	1365	526
Lac^c^	432 (27.1)	364 (18.6)	401 (33.2)	373 (27.3)	215 (41.0)
Gal^d^	699 (43.9)	472 (24.1)	365 (30.2)	365 (26.7)	163 (31.0)
Glc^e^	497 (31.2)	209 (10.7)	307 (25.4)	301 (22.0)	135 (25.6)
Rha^f^	260 (16.3)	151 (7.7)	174 (14.4)	155 (11.4)	129 (24.5)

a Cancer cell lines used for extraction of LSBPs: Suit2-007, ASML, BXPC3 (pancreatic cancer), MCF7 (breast cancer) and LST (colon cancer).

b The number of LSBPs quantified per cell line.

c Lactose,

d galactose,

e glucose and

f rhamnose represent the sugars added to the mobile phase to inhibit the binding of LSBPs by competition. *n* (%) represents the number and relative percentage of sugar-sensitive LSBPs prevented from binding to the resin in the presence of each sugar.

Gal- and Glc-sensitive LSBPs were highest in Suit2-007 and lowest in ASML. These can be ranked in the following order: Suit2-007 > LST > BXPC3 > MCF7 > ASML. On the other hand, Rha- and Lac-sensitive LSBPs were predominant in the LST cell line but less so in the ASML cell line and can be ranked for Rha in the following order: LST > Suit2-007 > BXPC3 > MCF7 > ASML and for Lac in the following order: LST > BXPC3 > MCF7 > Suit2-007 > ASML. From the MS analyses, these cell lines can be ranked based on the total number of LSBPs quantified in the following order: ASML > Suit2-007 > MCF7 > BXPC3 > LST.

### Genes Modulated by Khasianine in Human PDAC Suit2-007 Cells

**Figure 2:**
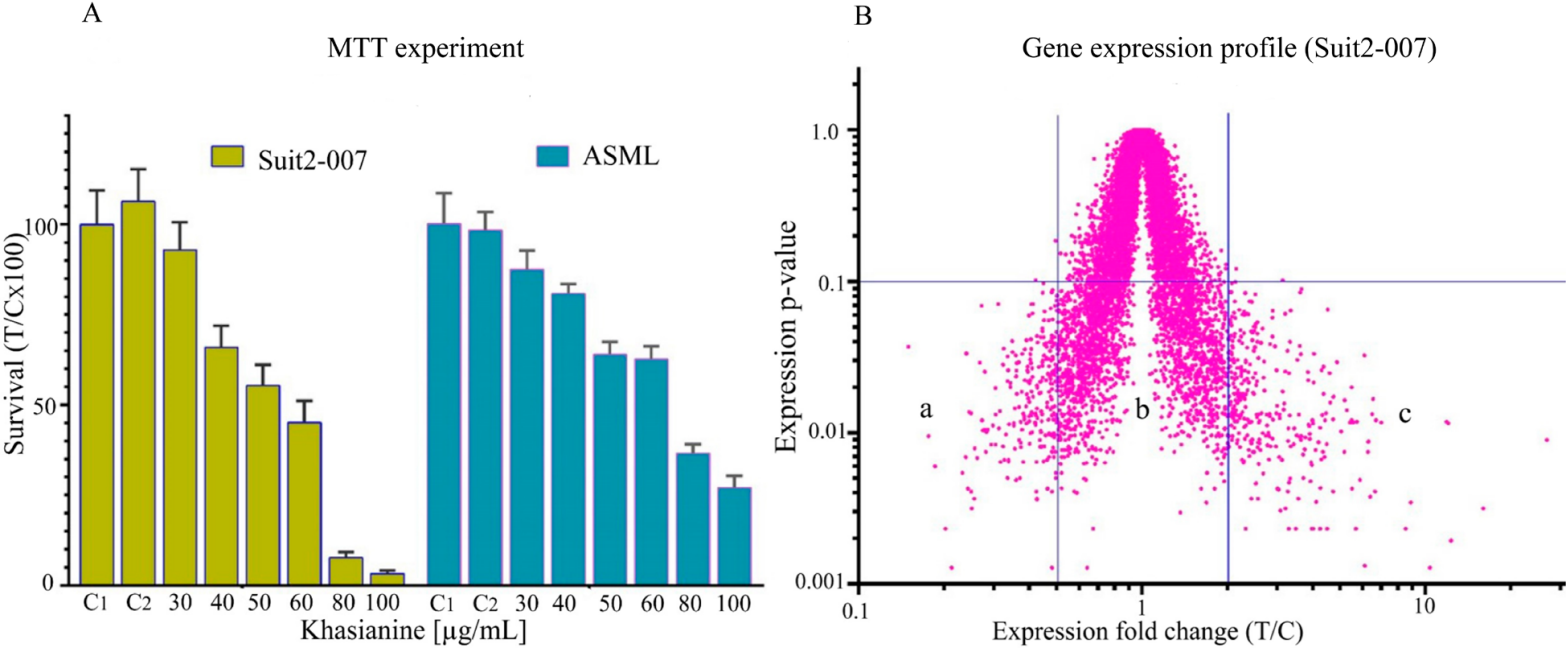
**MTT and chip array experiments.** A shows the effect of khasianine on human Suit2-007 and rat ASML cell lines at 48 h as evaluated by MTT. C1 and C2 represent untreated and vehicle controls, respectively. The experiment was repeated thrice, and final readings were obtained from an average of 8 wells per concentration. B shows a dot plot analysis for the gene expression profile of Suit2-007 cells (from a chip array) that were treated (three replicates) with khasianine (50 μg/mL) for 48 h.

Khasianine inhibited the proliferation of Suit2-007 and ASML cells with IC_50_ values of 50 and 54 μg/mL, respectively (Figure 2A). This finding is in agreement with its activity against other cancer cell lines.^23^ To identify genes modulated by khasianine, we performed a chip array for Suit2-007 cells. With Ingenuity Pathway Analysis (IPA), we analyzed the data and grouped the genes according to their expression fold change. The effect of khasianine on Suit2-007 at the mRNA level is shown in Figure 2B. The observed symmetry for genes in the central rectangle termed “b” (*p* > 0.1) is not replicated for rectangles “a” and “c” (*p* < 0.1), the RNAs of which showed a particular expression.

### Khasianine Downregulated Most LSBPs from Human PDAC Suit2-007 Cells

For evaluating the effect of khasianine at protein level, we focused on LSBPs, which showed affinity for the lactosyl resin and related sugars as reported elsewhere.^12,13^ We presumed that khasianine could modulate LSBPs since it contained the disaccharide unit Rha-Glc. We used IPA to extract gene IDs for sugar-sensitive LSBPs from chip array and MS data for Suit2-007 cells treated by khasianine. The extracted data contained expression fold changes/label-free quantification ratios (LFQs) for individual sugar-sensitive LSBPs. Tables 2 and S3–S6 summarize the significantly modulated sugar-sensitive LSBPs at protein and mRNA levels after the cells were treated with khasianine.

**Table 2:** Sugar-Sensitive LSBPs from Suit2-007 Cells Modulated by Khasianine

Sugar	No. of LSBPs	↓ Khas, at mRNA, *n* (%)^a^	↑ Khas, at mRNA, *n* (%)^a^	↓ Khas, LFQ protein, *n* (%)^b^	↑ Khas, LFQ protein, *n* (%)^b^
Gal	699	126 (18.0)	78 (11.2)	142 (20.3)	15 (2.2)
Glc	497	84 (16.9)	56 (11.3)	85 (17.1)	8 (1.6)
Lac	432	239 (55.3)	63 (14.6)	315 (72.9)	17 (3.9)
Rha	262	49 (18.7)	24 (9.2)	149 (56.9)	10 (3.8)

a Number (*n*) and respective percentage (%) of LSBP genes downregulated (↓) or upregulated (↑) for the respective LSBP subgroups.

b Number of LSBPs identified by label-free quantification mass spectrometry (LFQ-MS) and either downregulated (↓) or upregulated (↑) in the respective LSBP subgroups.

**Figure 3:**
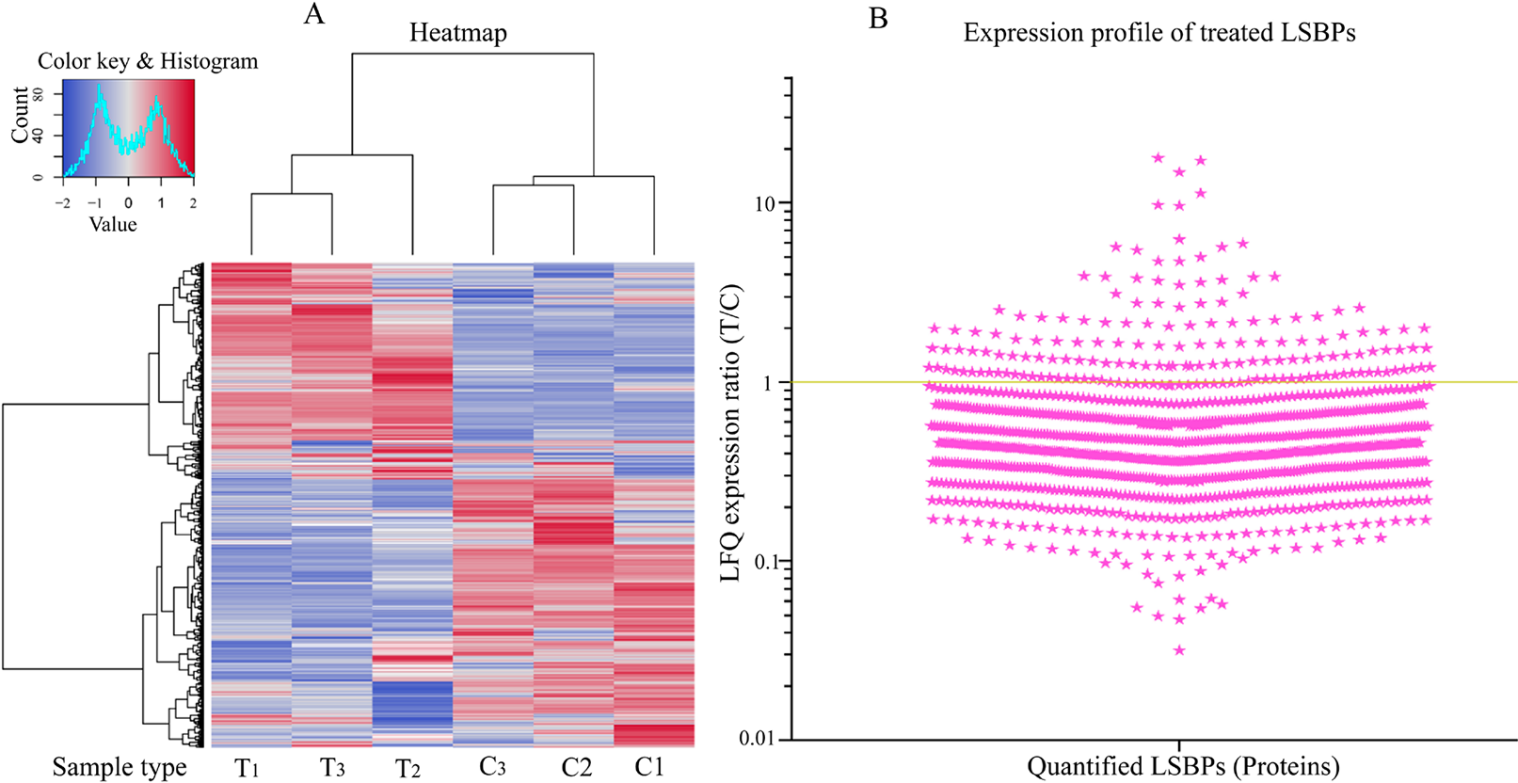
**MS analysis of LSBPs fractions isolated from khasianine-treated Suit2-007 cells.** A represents a heatmap of *Z*-scored LFQ intensities for treated (T1–T3) versus control (C1–C3) samples. The dot plot in B represents the MS-analyzed LSBPs, depicting more proteins significantly downregulated (LFQ ratio ≤0.5) than upregulated.

When comparing the number of sugar-sensitive LSBPs downregulated by khasianine at mRNA and protein levels, the following order was observed: Lac > Rha > Gal > Glc. On the other hand, when considering sugar-sensitive LSBPs upregulated by khasianine at both mRNA and protein levels, Lac-sensitive LSBPs emerged as the most upregulated while Rha- (mRNA level) and Glc-sensitive LSBPs (protein level) were the least upregulated. A heatmap and dot plot analysis for modulated LSBPs are shown in Figure 3.

### Expression of Sugar-Sensitive LSBP Subgroups in Data from the Rat Model and Patients

To determine the expression levels of sugar-sensitive LSBPs from in vivo samples, we used three chip array data sets from two rat models and patients. The first set was derived from Suit2-007 cells re-isolated from tumors growing in the liver environment of nude rats after intraportal implantation into this organ.^26^ The second set was chip array derived from ASML cells growing at different stages of liver colonization in the immunocompetent BDX rat.^27,28^ The third set was from pancreatic cancer patients (tumor versus normal tissue) downloaded from the GEO (ID: GSE71989) data base.^11^

**Figure 4:**
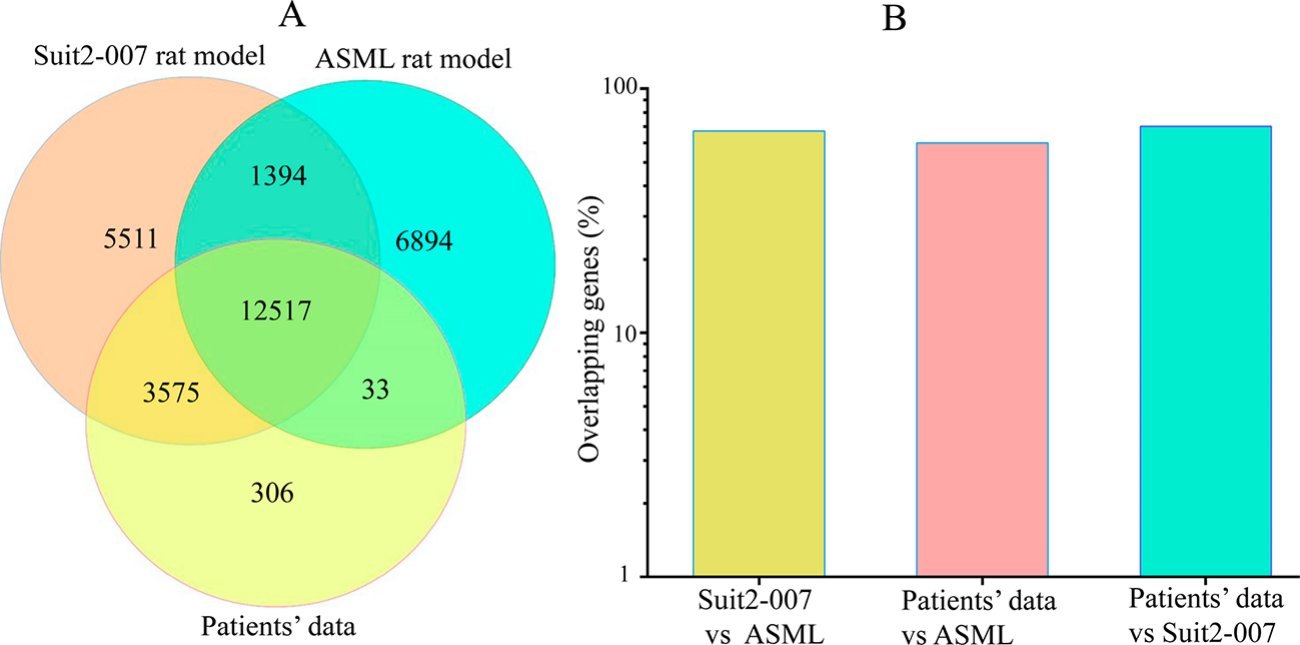
**Overlapping genes between data from the rat models and patients.** A shows the number of genes overlapping between data from patients and two PDAC cell lines (rat ASML and human Suit2-007). B shows the percentage gene overlap from the Venn diagram: 67% for Suit2-007 versus ASML, 60% for patients versus ASML and 70% for patients versus Suit2-007. Chip array data for ASML and Suit2-007 cell lines were derived from the liver of immunocompetent BDX and nude rats, respectively. Data for patients were downloaded from the GEO data base.

The total numbers of genes expressed in data from the rat models and in patients are depicted in Figure 4. More genes (70%) from the patients’ data were expressed in human Suit2-007 cells growing in nude rats than in ASML cells growing in rats (60%). When comparing the two rat models, only 67% of genes expressed in human Suit2-007 cells were detected in the rat ASML cells. The difference in the number of genes between patients and human Suit2-007 (30%) resulted from filtering and data processing from patients by the IPA. The numbers of processed genes from human Suit2-007, rat ASML and patients were *n* = 22997, *n* = 16431 and *n* = 20838, respectively.

From the three data sets we extracted gene IDs for sugar-sensitive LSBPs and their respective fold changes. Tables 3 and S7 show the number of significantly modulated LSBP genes in these data sets. Rha- and Lac-sensitive LSBPs were the most significantly upregulated subgroups in the Suit2-007 rat model and data from patients. The upregulated sugar-sensitive LSBP genes in Suit2-007 cells can be ranked in the following order: Rha > Lac > Glc > Gal. Similarly, the upregulated sugar-sensitive LSBP genes from patients’ data can be ranked in the following order: Rha > Lac > Gal > Glc. When considering the sugar-sensitive LSBPs downregulated in data from the rat and patients, Lac- and Glc-sensitive LSBPs were the most downregulated. For the Suit2-007 rat model alone, the ranking follows the order: Lac > Rha > Gal > Glc. For the patients’ data alone, the following order was observed: Glc > Gal > Lac > Rha.

**Table 3:** Sugar-Sensitive LSBPs Modulated in Data from Suit2-007 Rat Model and Patients

Sugar	No. of sugar-sensitive LSBPs	↓ Suit2-007 rat model, *n* (%)^a^	↑ Suit2-007 rat model, *n* (%)^a^	↓ PDAC patients, *n* (%)^b^	↑ PDAC patients, *n* (%)^b^
Gal	699	49 (7.0)	33 (4.7)	393 (56.2)	142 (20.3)
Glc	497	26 (5.2)	25 (5.0)	299 (60.5)	90 (18.1)
Lac	432	33 (7.6)	38 (8.8)	240 (55.6)	96 (22.2)
Rha	262	20 (7.6)	30 (11.5)	140 (53.4)	60 (22.9)

a Number (*n*) and respective percentage (%) of LSBP genes downregulated (↓) or upregulated (↑) in Suit2-007 re-isolated from the rat liver metastasis model.

b Number of LSBP genes downregulated (↓) or upregulated (↑) in data from patients.

### Signaling Pathways Associated with Sugar-Sensitive LSBPs

**Figure 5:**
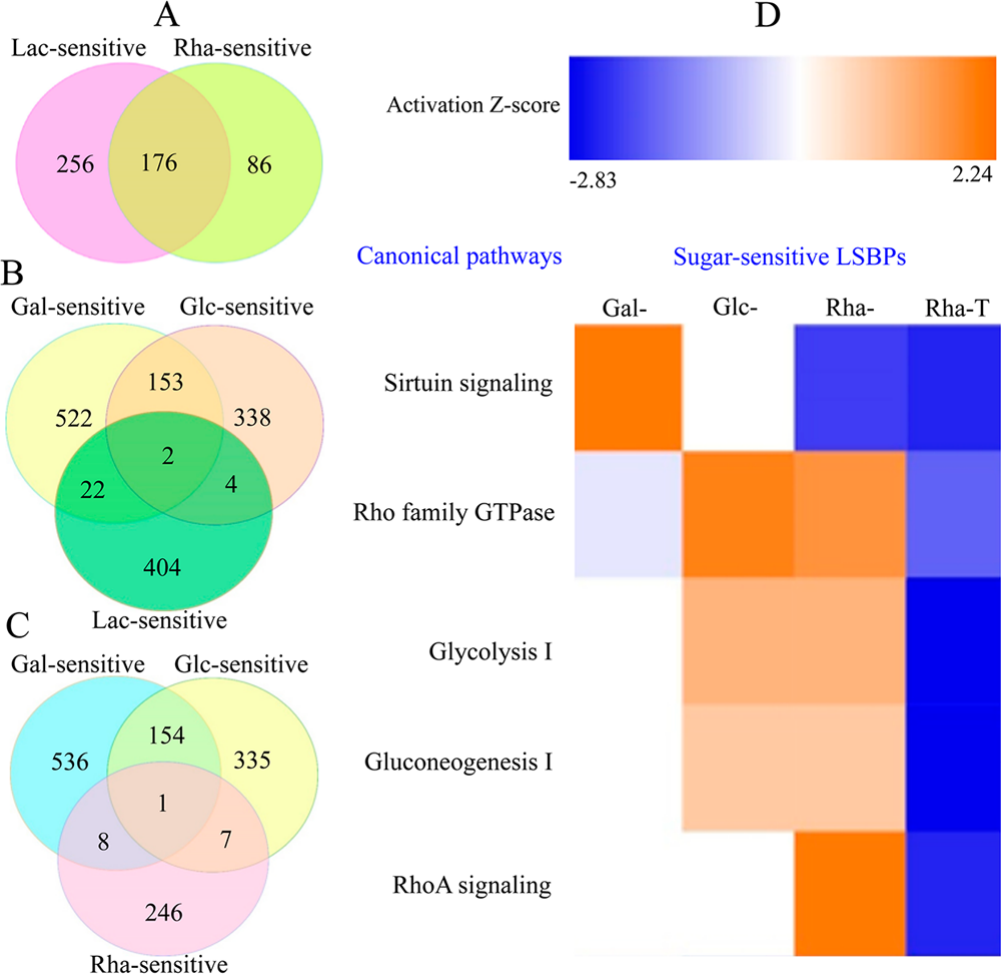
**Overlapping genes for sugar-sensitive LSBPs and significant signaling pathways.** Significant gene overlap was observed between Lac- and Rha-sensitive LSBPs (A) as well as between Gal- and Glc-sensitive LSBPs (B and C). Venn diagram analysis of Lac- or Rha-sensitive LSBPs with Gal- and Glc-sensitive LSBPs resulted in little gene overlap. D shows signaling pathways associated with sugar-sensitive subgroups. Key pathways with a *Z*-score >2 include Sirtuin, RhoGTPase and RhoA. The effect of khasianine treatment on these pathways is shown in the last column, Rha-T.

To identify key signaling pathways associated with sugar-sensitive LSBPs, we performed comparative IPA. First, we performed Venn analyses to determine the number of common genes between individual LSBP subgroups. Using the dendrogram depicted in Figure 1C as a basis for further analysis, we first compared Rha- versus Lac-sensitive LSBPs and then Gal- versus Glc-sensitive LSBPs with either Rha- or Lac-sensitive LSBPs. In the former, 67% of Rha-sensitive LSBPs overlapped compared to 41% of Lac-sensitive LSBPs. For Glc- versus Gal-sensitive LSBPs, 31% of Glc- overlapped with Gal-sensitive LSBPs, whereas only 23% of Gal- overlapped with Glc-sensitive LSBPs (Figure 5A–C).

Next, we used data from patients to determine the signaling pathways corresponding to individual sugar-sensitive LSBPs. We preferred these data since sugar-sensitive LSBPs were significantly altered and therefore amenable to analysis compared to the data from the rat model. From this analysis, we filtered 5 canonical pathways as shown in Figure 5D.

From these pathways, only three were significantly activated and were therefore of interest for further investigation. These include the Sirtuin pathway (specific for Gal-sensitive LSBPs), the RhoGTPase pathway (specific for Glc-sensitive LSBPs) and the RhoA pathway (specific for Rha-sensitive LSBPs). While the Sirtuin and RhoGTPase pathways are important with respect to sugar-sensitive LSBPs, we focused on the RhoA pathway for two reasons. Firstly, the biological activity of khasianine is attributed to the presence of Rha within its structure. Secondly, when compared to other subgroups, Rha-sensitive LSBPs were ranked higher with respect to the number of significantly upregulated LSBPs in data from the rat model and patients.

**Figure 6:**
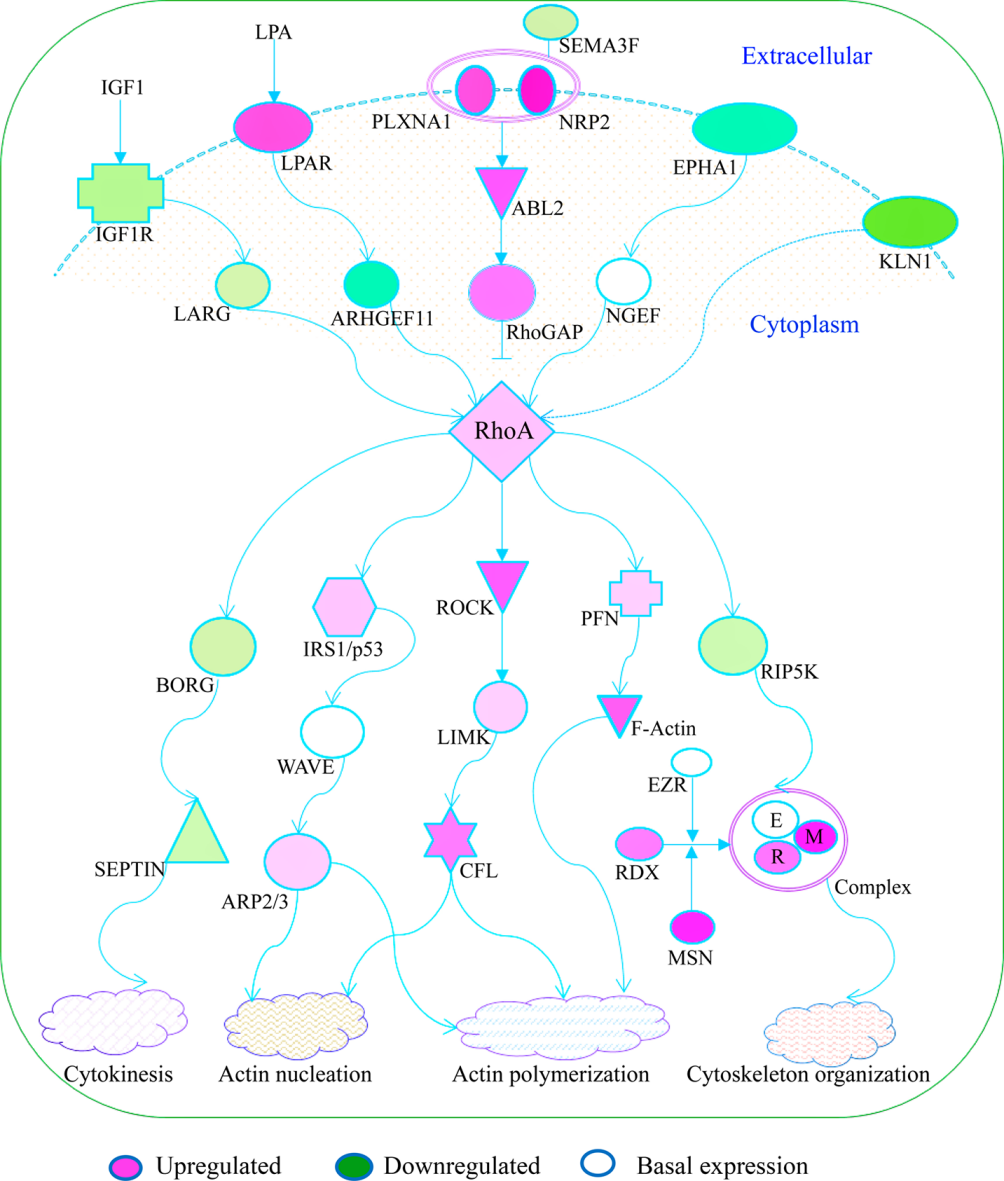
**The RhoA pathway and Rha-sensitive LSBPs.** The RhoA pathway was the most activated in Rha-sensitive LSBPs corresponding to genes expressed in data from patients. The expression fold change was obtained by comparing tumor versus non-tumor tissue. RhoA cycles between active GTP-bound (GTP-RhoA) and inactive GDP-bound (GDP-RhoA) states. The pathway is deemed activated based on a predicted activation state and significant *Z*-score (>2). RhoA receives signals from a lysophosphatidic acid receptor and, in turn, activates downstream elements, including insulin receptor substrate1/tumor protein P53, Rho-associated coiled coil-containing protein kinase and profilin. These proteins then trigger signals that regulate cell movement (actin polymerization) via LIM domain kinase, cofilin and F-actin. In the cytosol, ezrin, radixin and moesin interact to form a complex, which induces organization of the cytoskeleton.

By using the IPA-generated RhoA pathway as a guideline, we re-constructed the pathway using the expression fold changes for individual genes showing sensitivity to Rha (Figure 6). The RhoA pathway depicts significant modulation of genes, which are denoted by pink and green symbols for upregulation and downregulation, respectively. Though the overexpression of RhoGAP may imply an inactivation state of the RhoA pathway, its significant activation *Z*-score (>2) points to a pathway that is operational. When activated upstream, RhoA sends signals to immediate genes downstream, which in turn communicate to downstream elements that modulate various functions, including cytokinesis, actin nucleation, polymerization and organization of the cytoskeleton.

### Key Signaling Pathways of Suit2-007 Cells Downregulated by Khasianine Treatment

We also investigated how khasianine treatment altered the expression of 260 Rha-sensitive LSBPs in the identified signaling pathways. As depicted in the Rha-T column of Figure 5, khasianine-treated Suit2-007 cells resulted in the downregulation of the Sirtuin, RhoGTPase and RhoA pathways. By further analysis, we identified three functional annotations associated with the RhoA pathway. These annotations included cellular movement, cell signaling and interaction and cell cycle. Under these annotations were gene clusters with altered expression profiles, an indication of their role in metastasis. A summary of these analyses is given in Table 4 and the full list of altered genes in Table S6.

**Table 4:** Functional Annotations Associated with Rha-Sensitive LSBPs in the RhoA Pathway

Functional annotations^a^	Diseases or Functions^b^	Predicted activation^c^	Activation *Z*-score^d^	Selected molecules^e^
Cell movement	Cell movement	Decreased	–4.91	83/291
Cell signaling and interaction	Interaction of tumor cells	Decreased	–4.56	4/65
Cell cycle	Cell cycle progression	Decreased	–2.73	32/149

a Functional annotations represent broader functions in which various gene clusters are annotated.

b Functions represent the predicted roles of individual biological annotations.

c Predicted activation state represents the overall state of the annotations of the Rha-sensitive LSBPs.

d Activation *Z*-score is a parameter that shows whether a biological function is increased or decreased.

e Molecules represent the number of Rha-sensitive LSBPs identified within the RhoA pathway.

## Discussion

Prompted by the need to discover effective therapies for treating pancreatic cancer, we investigated the antiproliferative activity of khasianine and its overall effect on sugar-sensitive LSBPs. Our interest in this compound was piqued by the influence of rhamnose in the biological activity of solasodine glycosides. To date, only a few studies have investigated khasianine as a potential anticancer compound. Most studies have, however, focused on related compounds, including solamargine and solanine, which differ from khasianine not only in configuration but also by the number of sugars in the carbohydrate unit.^29^ We considered a study with pancreatic cancer cells to be particularly interesting because they express LSBPs that bind monosaccharides and are also modulated in data from the rat model and patients.^13,26^

We isolated khasianine from *S*. *incanum* using the method described by Ding et al. (with modification) and evaluated its activity in pancreatic cancer cells.^30^ The identity and purity (>90%) of the isolated compound were confirmed by LC-MS and NMR. In vitro, khasianine exhibited comparable efficacy in Suit2-007 and ASML cell lines, albeit with a slightly higher IC_50_ in the latter. This activity (believed to be influenced by rhamnose) was within the range reported in the literature but lower than that of glycoalkaloids containing three sugars.^23,31^

To evaluate the effect of khasianine on proteins showing sensitivity to sugars, we first isolated these proteins by competitive affinity chromatography, as reported elsewhere.^13^ We modified and optimized the isolation procedure by using the same concentration for the sugars (Lac, Gal, Glc and Rha), which were added to the mobile phase. The resulting protein fractions (sugar-sensitive proteins) that eluted in the mobile phase were identified and thus served as a basis for evaluating the effect of khasianine on sugar-sensitive LSBPs.

At protein level, khasianine impacted the expression of LSBPs from human Suit2-007 cells by downregulating more proteins than those upregulated. When considering the effect of khasianine on individual LSBP subgroups (at mRNA level), Lac-sensitive LSBPs were the most downregulated, followed by the Rha-sensitive subgroup. In essence, we observed a significant gene overlap (67%) between Lac- and Rha-sensitive subgroups. This was unexpected since Rha is not a component of Lac, which is composed of Gal and Glc. We could therefore anticipate an overlap between the Lac-sensitive subgroup with either Gal or Glc or both subgroups. With further analysis, we observed a significant amount of gene overlap between Gal- and Glc-sensitive LSBPs which was, however, less compared to that between Lac- and Rha-sensitive LSBPs. This was not unexpected considering that Gal and Glc differ only by the C-4 configuration. Moreover, both sugars have been shown to interact with the aromatic residues of proteins via H-3 and H-5.^32,33^

To establish whether the sugar-sensitive LSBPs modulated in vivo were affected by khasianine treatment under in vitro conditions, we used chip array data from the rat model and patients. First, we examined to what extent the gene expression from pancreatic cancer cells growing in rat liver overlapped with those from the chip array of pancreatic cancer patients. This evaluation revealed that 70% of genes were common between human Suit2-007 PDAC cells and patients’ data. The observed shortfall (30%) in the overlap resulted from the automatic exclusion of those genes in patients’ data which were not recognized by the IPA program.

The analysis of data with IPA revealed that Rha- and Lac-sensitive subgroups were the most upregulated LSBPs detected in the rat model (Suit2-007) and patients. On the other hand, Lac- and Glc-sensitive subgroups were the most downregulated LSBPs in these data sets. When examining the effect of khasianine on sugar-sensitive LSBPs, the Rha subgroup was most upregulated and downregulated by khasianine treatment. These findings point to a possible involvement of Rha-sensitive LSBPs in tumor progression, which can be targeted by khasianine.

The mechanism by which solasodine glycosides inhibit cell proliferation is not well understood. Previous studies have, however, reported the existence of EELs in tumor cells, which bind Rha. Presently, there is no sufficient data regarding the interaction of solasodine glycosides with the reported EELs. Nevertheless, it is believed that when solasodine glycosides (such as solamargine) are exposed to tumor cells, they interact with EELs forming a complex that gets internalized. In the cell, the ligand gets degraded (inducing apoptosis), setting free the EEL receptor, which is recycled to the cell surface.

Irrespective of the mechanism involved, the application of solasodine glycosides as a cancer therapy is widely documented.^34^ For instance, the treatment of squamous cell carcinoma with a standard mixture containing 33% solamargine, 33% solasonine and 34% di- and monoglycosides yielded promising results.^35^ Even with these reports, there could be concerns about the potential of khasianine as a drug because its molecular weight (720 kDa) exceeds the 500 kDa threshold according to the Lipinski’s rule of five (Ro5). Compounds that defy this rule, i.e. beyond the rule of five (bRo5), have poor metabolism and pharmacokinetic properties (such as low permeability, low solubility and high metabolic clearance)^36^ and could, therefore, pose challenges as a possible therapy. Despite these limitations, bRo5 compounds have demonstrated high affinity and ability to modulate difficult-to-treat drug targets compared to those that comply with the Ro5.^37^

In pancreatic cancer, proteins involved in signaling cascades hold promise as targets of therapy.^38^ We therefore sought to identify signaling pathways associated with sugar-sensitive LSBPs. From IPA, we identified Sirtuin, RhoGTPase and RhoA as the most activated (*Z*-score >2) pathways for Gal-, Glc- and Rha-sensitive LSBPs, respectively. Considering the role of Rha in the biological activity of solasodine glycosides, we singled out the Ras homolog family member A (RhoA) pathway as the most relevant for further investigation. By correlating literature information with gene expression profiles from patients’ data, we re-constructed this pathway and examined its role in tumor progression. RhoA is a multifunctional protein family of GTPases within the Ras homology proteins, which regulates cellular functions by cycling between the active (GTP-bound) and inactive (GDP-bound) states and is inhibited by RhoGAP.^39^ However, in some instances, constitutively GTP-bound RhoA remains activated and can be regulated by different mechanisms.^40^ Because of these switch-on/switch-off states, it performs cellular functions including regulation of actin polymerization, myosin contractility and the assembly of intermediate filament.^41^ In the context of metastasis, RhoA regulates cell migration, cell proliferation, cell signaling, oncogenic transformation and cell–matrix interactions.^42−45^ Here, we demonstrate that the RhoA pathway is activated (*Z*-score >2) in Rha-sensitive LSBPs from patients’ data. These LSBPs were significantly downregulated in human PDAC Suit2-007 cells treated by khasianine. Further analysis revealed that these proteins were involved in biological functions linked to metastasis, which include cell movement (28.5%), cell signaling and proliferation (6%) and cell cycle regulation (21.5%).

## Conclusion

We have demonstrated that khasianine inhibits the proliferation of pancreatic cancer cells. At mRNA and protein levels, khasianine modulated Lac-sensitive LSBPs, which significantly overlapped with the Rha-sensitive subgroup. The RhoA pathway was significantly modulated in the Rha-sensitive subgroup of patients’ data and significantly downregulated by khasianine treatment in vitro. As these findings underscore the potential of khasianine as a therapy for pancreatic cancer, further studies will be required to evaluate its activity in vivo.

## Experimental Section

### Extraction and Purification of Khasianine from *S. incanum*

Chemicals and reagents were purchased from either Sigma Aldrich or Fischer Scientific unless otherwise indicated.

Fruits from *S. incanum* were obtained from the Kasarani area in Nairobi and stored at Kenyatta University. Specimens were deposited in the University of Nairobi herbarium upon identification by an experienced taxonomist, and a voucher specimen (JCM/2013) was issued. Fruits were cut into small pieces and kept in the shade for 4 weeks. The dried fruits were then ground into a powder and transported to Germany after clearance by KEPHIS. The samples were stored in an airtight container at −20 °C until used.

The extraction of glycoalkaloids was performed according to the protocol of Ding et al. but with some modification.^30^ Dry fruit powder (80 g) was mixed with 70% aqueous ethanol (250 mL) and vortexed for 3–4 h at room temperature. The supernatant of the ethanol extract was centrifuged at 5000*g* for 30 min. The process was repeated by adding fresh ethanol to the sediment. The combined ethanol extracts were removed under reduced pressure at 45 °C, resulting in an oily residue (2.40 g). The extract was dissolved in 2% conc. HCl, vortexed and then mixed with 5% NaOH (VWR Chemicals, Prolabo Chemicals, Germany) to pH 11. Further extraction was performed by adding 50 mL of the extract with 50 mL water–*n*-butanol. After phase separation, the upper *n*-butanol phase was removed and the aqueous phase extracted with fresh *n*-butanol. The *n*-butanol phases were combined, and the solvent was removed under reduced pressure to give a greenish oily residue (1.2 g).

### Isolation of Khasianine by Silica Gel Column Chromatography

The *n*-butanol extract (1.2 g) was immobilized on coarse silica gel (5 g, pore size 60 Å). The loaded silica gel was layered on top of a column (38 × 4.5 cm) packed with silica gel 60 in hexane. Fractions were eluted with 250 mL aliquots of the following solvents: *n*-hexane, DCM, methanol in DCM (1, 2, 5, 10, 20, 30 and 50%) and then finally methanol. Solvents were removed under reduced pressure and the dried fractions re-dissolved in methanol (5 mL). For identification of the fractions containing the compound of interest, 25 μL aliquots were analyzed by semipreparative HPLC. A flowchart outlining this procedure is presented in Figure S1.

### Analysis of Fractions by Semipreparative HPLC

Semipreparative HPLC was conducted on an HP 1100 liquid chromatograph (Agilent Technologies, Waldbronn, Germany) fitted with a Zorbax Phenyl-Hexyl reverse-phase (9.4 × 250 mm) C18 column (Agilent Technologies, Waldbronn, Germany). A mobile phase (3 mL/min) consisting of 2% acetic acid in water (solvent A) and acetonitrile (solvent B) was used. A solvent gradient was applied over a total run time of 50 min: initially 100% A for 10 min, reducing to 90% A over 1 min, then reducing to 80% A over 9 min, then reducing to 60% A over 10 min, then reducing to 40% A over 10 min and then finally to 0% A over 10 min.

### Purification and Analysis of Pooled Fractions

The three fractions that were eluted with increasing concentrations of methanol (20, 30 and 50%) in DCM were pooled and subjected to column chromatography. Fractions were collected and evaluated by TLC and then further analyzed by LC-MS to identify those fractions containing only khasianine. For LC-MS analysis, an Agilent 6120 Infinity instrument equipped with ESI, quadrupole and a Kinetex 2.6 μm C18 100 Å column (50 × 2.1 mm) was used. The analysis was run using water with 0.01% formic acid (solvent A) and acetonitrile with 0.01% formic acid (solvent B) at flow rate of 0.6 mL/min with the temperature maintained at 40 °C. The gradient consisted of 99% A → 10% A over 6 min followed by 10% A → 1% A over 2 min.

### NMR Spectroscopic Analysis

NMR analysis was performed to characterize the isolated compound with respect to the commercial sample (Hölzel Diagnostika Handels GmbH, Köln, Germany). Spectra were acquired at 25 °C in *d*_5_-pyridine using a Bruker Avance NMR spectrometer. The instrument was equipped with a 5 mm inverse-configuration probe with triple-axis-gradient capability at a field strength of 14.1 T, operating at 600.1 and 150.9 MH*z* for ^1^H and ^13^C nuclei, respectively. The δ’s of ^1^H and ^13^C nuclei are reported relative to the downfield signal of *d*_5_-pyridine (δ_H_ = 8.74 ppm and δ_C_ = 149.79 ppm). The δ’s of ^1^H nuclei are reported to three decimal places when the multiplet was amenable to first-order analysis or to two decimal places when the multiplet was beyond such interpretation. For overlapped signals, δ’s were taken from 2D NMR spectra and are reported to three decimal places to distinguish them. General NMR experimental and acquisition details for 1D ^1^H, ^13^C and DEPT observation and standard gradient-selected 2D COSY, HSQC and HMBC spectra, and routine δ assignment using 2D NMR have been previously described.^46,47^ Pulse widths were calibrated following the described protocol.^48^

### Cell Proliferation Assay

For the antiproliferation assay, MTT (3-(4,5-dimethylthiazol-2-yl)-2,5-diphenyltetrazolium bromide) was used as described elsewhere.^26^ In brief, Suit2-007 cells were prepared in complete RPMI 1640 medium (Gibco, Fischer Scientific, Germany) and dispensed into 96-well plates (4000 cells/well). The plates were kept for 24 h in standard cell culture conditions to allow the cells to attach. The cells were treated with various concentrations (30, 40, 50, 60, 80 and 100 μg/mL) of khasianine in ethanol. Cells treated with media only as well as khasianine-free ethanol served as controls. The plates were further incubated for 48 h, after which 20 μL/well (from a stock 10 mg/mL solution in PBS) of MTT solution (SERVA Electrophoresis GmbH, Heidelberg, Germany) was added. Plates were again incubated for 3 h, followed by the addition of 100 μL of 2-propanol solution in 0.04% N HCl. The experiment was performed thrice and yielded similar results. The absorbance was measured in triplicate using an ELISA reader (Biotech Instruments, Germany) at 540 nm (excitation) and 690 nm (reference) wavelengths. The IC_50_ values for two PDAC cell lines, Suit2-007 and ASML, were determined from 8 replicates/well for each concentration.

### Gene Profiling by Chip Array

For chip array, Suit2-007 cells in complete RPMI 1640 medium were seeded (in triplicate) in 6-well plates (2.5 × 10^5^ cells/well) and kept for 24 h in standard culture conditions to allow the cells to attach.^26^ Cells were then treated with 50 μg/mL of khasianine and incubated for 48 h. Thereafter, cells were harvested, placed in Eppendorf tubes and frozen in liquid nitrogen prior to total RNA isolation. Total RNA was isolated as detailed in the Fast Gene RNA isolation kit (Nippon Genetics Co. Ltd.). The concentrations of total RNA in samples were determined by a Nanodrop spectrophotometer and chip array performed according to the modified Eberwine protocol.^11,49^

### Isolation of LSBPs from Cancer Cell Lines

LSBPs were isolated from cell lysates for respective cell lines as described elsewhere.^13^ In brief, cell pellets were disrupted by lysis buffer [850 μL RIPA buffer: 50 mM TRIS, 150 mM NaCl, 1.0% NP-40, 25× protease inhibitor (40 μL), 10× Phosphostop tablet (100 μL) and 100 mM of NaVO_3_ (10 μL)] (all from Roche Diagnostics, Germany). The lysate was centrifuged (16400 rpm, 30 min) to obtain a clear supernatant. The protein concentrations were determined by Roti Nanoquant solution (Carl Roth GmbH & Co. KG, Germany). In addition, LSBPs were isolated from Suit2-007 cells treated with khasianine. Suit2-007 cells in complete RPMI 1640 medium were seeded in 6-well plates (2.5 × 10^5^ cells/well) and kept for 24 h in standard culture conditions.^26^ Cells were then treated with 50 μg/mL of khasianine and incubated for 48 h. Lysates were prepared as described above for the isolation of LSBPs.

LSBPs were isolated by affinity chromatography as described elsewhere.^13^ Briefly, clean lysate (325 μg/mL) was loaded onto the column at a low velocity flow (0.5 mL/min) in a loading buffer (20 mM TRIS-HCl and 20 mM Arg-HCl). The Tricon column was packed with lactosyl-Sepharose gel (Pharmacia Biotech, Sweden). LSBPs were then eluted from the column with a buffer containing TRIS-HCl (20 mM), Arg-HCl (20 mM), CaCl_2_ (100 mM) and Gal (100 mM). Thereafter the column was regenerated by washing with 1 M CaCl_2_. The fractions from different runs for Suit2-007 were cleaned of the binding buffer and analyzed by LFQ-MS.

### Separation of Sugar-Sensitive LSBPs from Suit2-007 Cells by Affinity Chromatography

Sugar-sensitive LSBPs were isolated as described elsewhere but with some modification.^13^ First, a column binding experiment was performed using the isolated LSBPs, and 325 μg/mL was found optimal for competitive affinity isolation. Aliquots of LSBP fractions from Suit2-007 cells were prepared in triplicate using Gal, Glc, Rha and Lac (Sigma-Aldrich, Germany). Loading buffers (20 mM TRIS-HCl and 20 mM Arg-HCl) were prepared containing 100 μM of each sugar and one without sugar as a control. Before each run, the samples were incubated for 15 min at 37 °C with gentle vortexing. After 5 min of column equilibration, a sample was injected to the column in the loading buffer. The proteins that were prevented from binding to the column in the presence of the sugar were collected. Similarly, proteins that were not affected by the sugar were eluted from the column with an elution buffer (20 mM TRIS-HCl, 20 mM Arg-HCl and 100 mM CaCl_2_). Concentrations of cleaned fractions were determined and samples analyzed by LFQ-MS.

### Statistical Analysis

Analysis of protein MS data was performed using MaxQuant.^50,51^ Proteins were analyzed by a label-free quantification.^52^ A cutoff was set at 0.01 for identifying false discovery rates for both peptides and proteins. MTT experiments were analyzed by GraphPad Prism. Data obtained from chip array were analyzed with Chipster, R and IPA. Genes were filtered at ±1.5 fold change and *p* < 0.01. Genes and proteins were evaluated by Venn diagrams.

## Supporting Information Available

Khasianine extraction flowchart. LC-MS chromatograms for khasianine. NMR spectroscopy data for khasianine. Isolation chromatograms for LSBPs and sugar-sensitive-LSBPs. Sugar-sensitive LSBPs quantified by LFQ-MS (PDF)
