# Supplementary material for: Khasianine Affects the Expression of Sugar-Sensitive Proteins in Pancreatic Cancer Cells, Which Are Altered in Data from the Rat Model and Patients
Source: ACS Pharmacol Transl Sci. 2023 Apr 13;6(5):727–37. doi: 10.1021/acsptsci.3c00013 (PMC10186360; doi:10.1021/acsptsci.3c00013)
Supplement: Supplementary file 1 — pt3c00013_si_001.pdf [file pt3c00013_si_001.pdf]

SUPPORTING INFORMATION

Khasianine affects the expression of sugar-sensitive proteins in pancreatic cancer cells, which are altered in data from the rat model and patients

Micah N. Sagini[†*], Karel D. Klika[‡*], Robert W. Owen[§], Martin R. Berger[†]

†Toxicology and Chemotherapy Unit, German Cancer Research Center (DKFZ)

Im Neuenheimer Feld 580, 69120 Heidelberg, Germany

‡Molecular Structure Analysis, German Cancer Research Center (DKFZ),

Im Neuenheimer Feld 280, 69120 Heidelberg, Germany

§Biochemistry and Biomarkers Unit, German Cancer Research Center (DKFZ),

Im Neuenheimer Feld 580, 69120 Heidelberg, Germany.

Corresponding author: Micah N. Sagini[†*]

E-mail: sagini2000@gmail.com

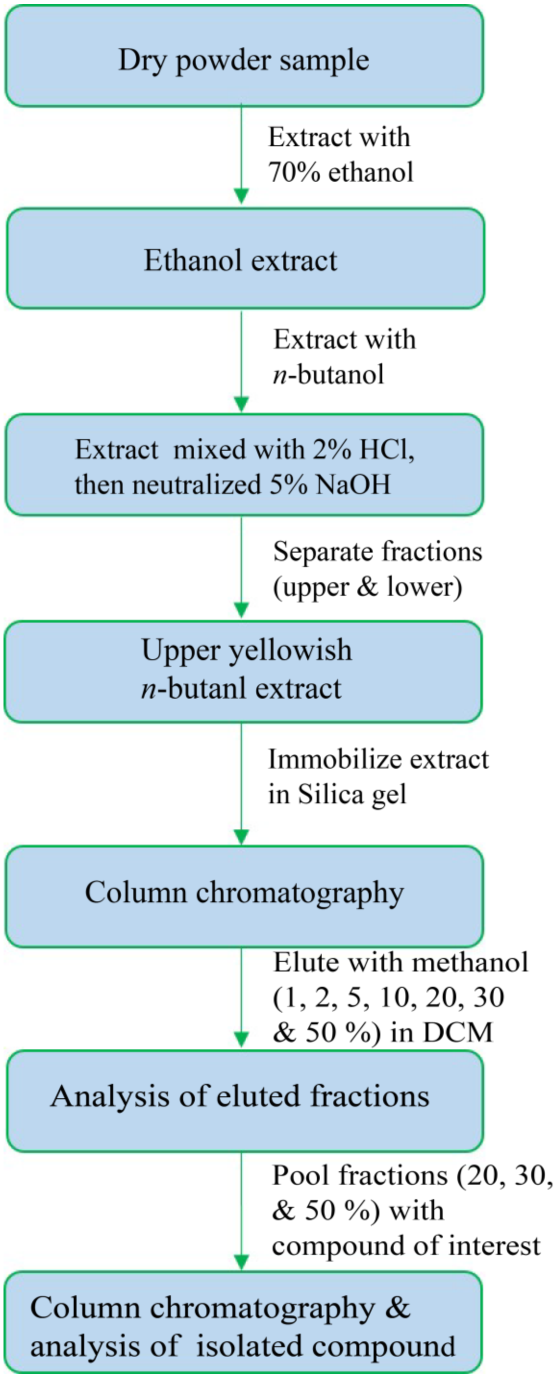

Figure S1. Extraction and isolation flowchart

The extraction and purification flowchart outlines the steps involved in the isolation of khasianine from *Solanum incanum* fruits.

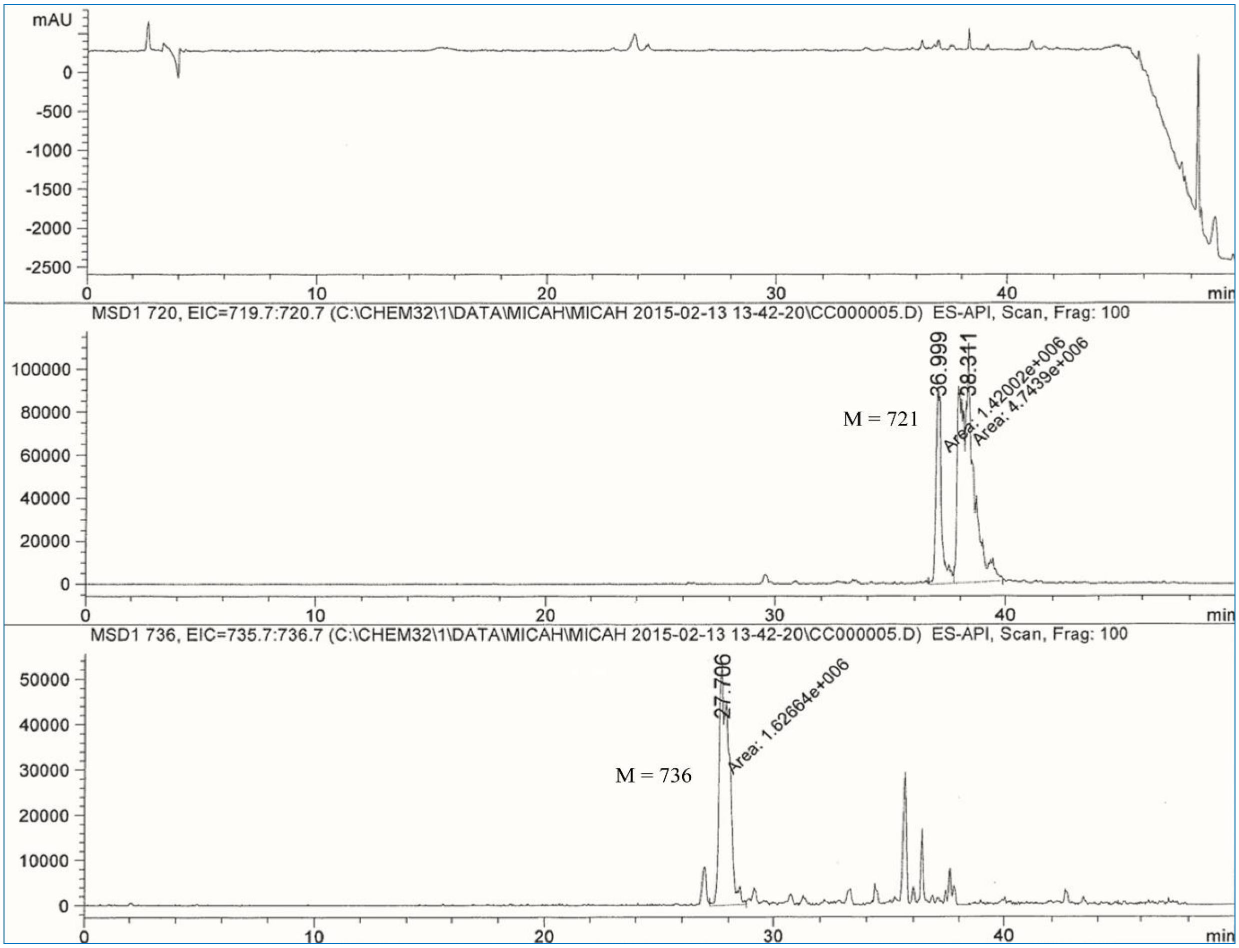

Figure S2A. LC-MS analysis of fraction eluted by silica gel column chromatography

UV (top) and SIMs (bottom two) chromatograms for the fraction eluted with 20% methanol in DCM over silica gel. The middle SIM chromatogram reveals the presence of two compounds of nominal mass 721 Da, the mass of khasianine. The bottom SIM chromatogram reveals the presence of an unknown compound of nominal mass 736 Da, possibly a diamino homologue of khasianine.

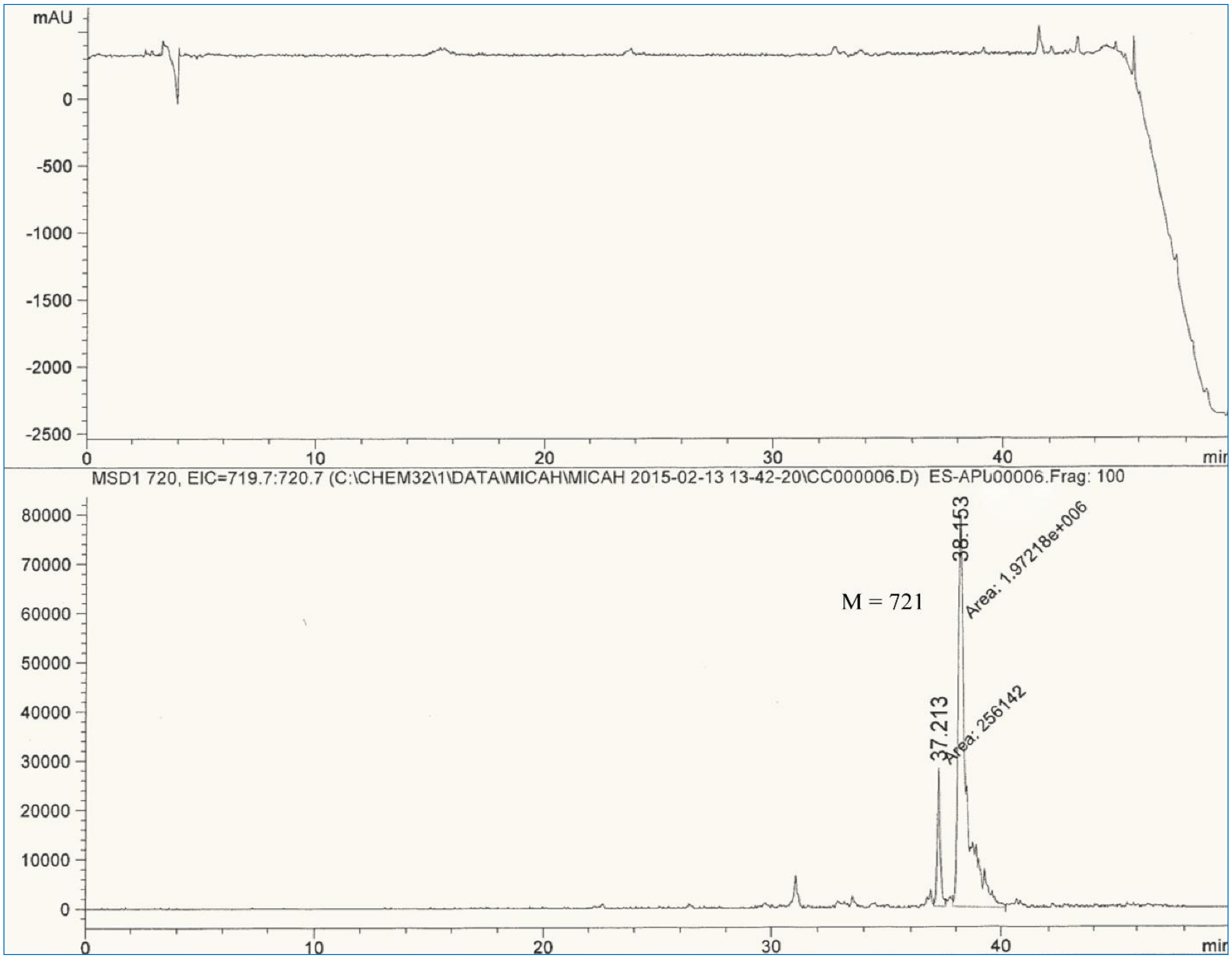

Figure S2B. LC-MS analysis of fraction eluted by silica gel column chromatography

UV (top) and SIM (bottom) chromatograms for the fraction eluted with 30% methanol in DCM over silica gel. The SIM chromatogram again reveals the presence of two compounds with nominal mass 721 Da but with the later eluting compound now in much greater relative abundance.

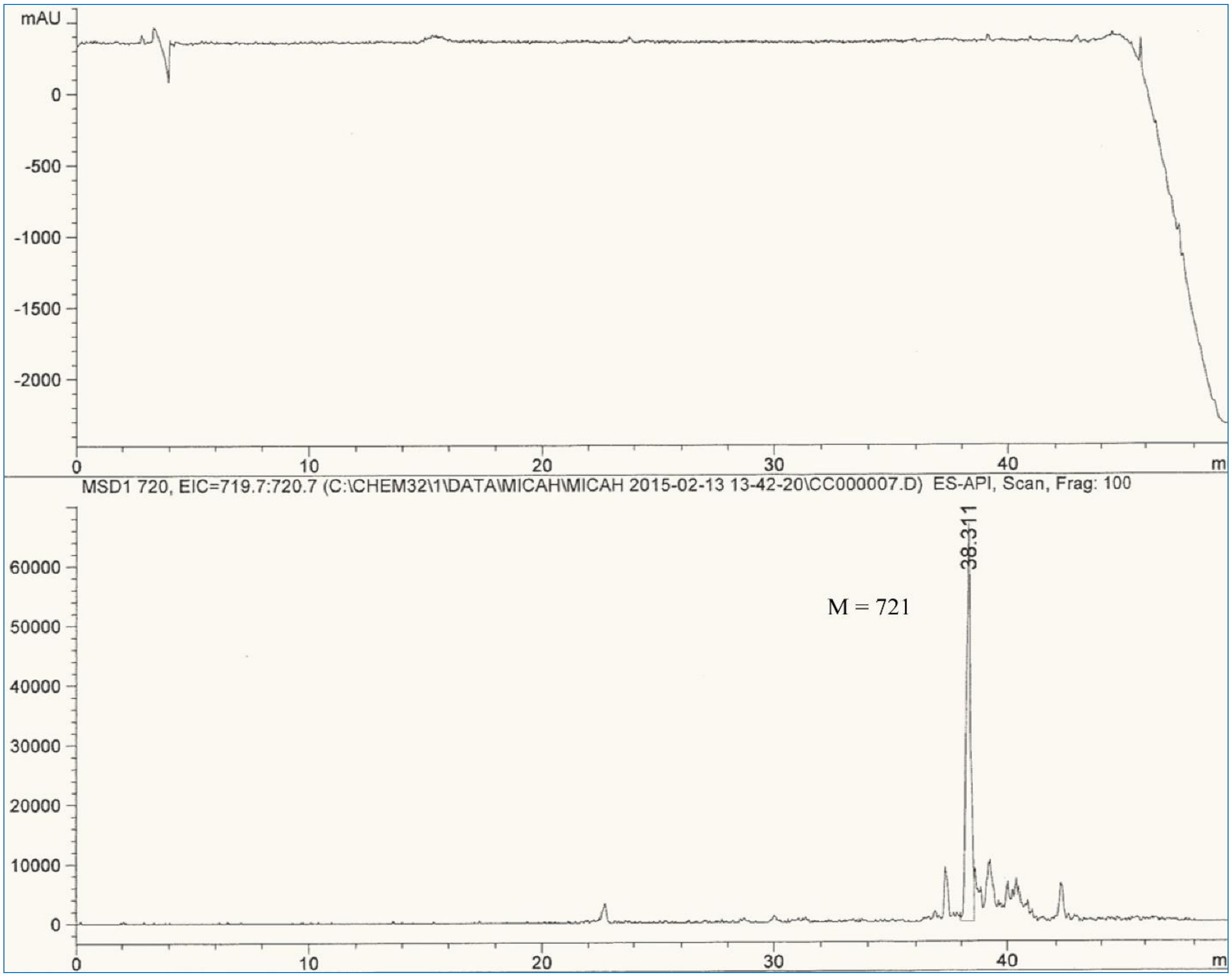

Figure S2C. LC-MS analysis of fraction eluted by silica gel column chromatography

UV (top) and SIM (bottom) chromatograms for the fraction eluted with 50% methanol in DCM over silica gel. The SIM chromatogram reveals the presence of practically only one compound of nominal mass 721 Da eluting at the same time as the major compound of mass 721 Da in Figure S2B.

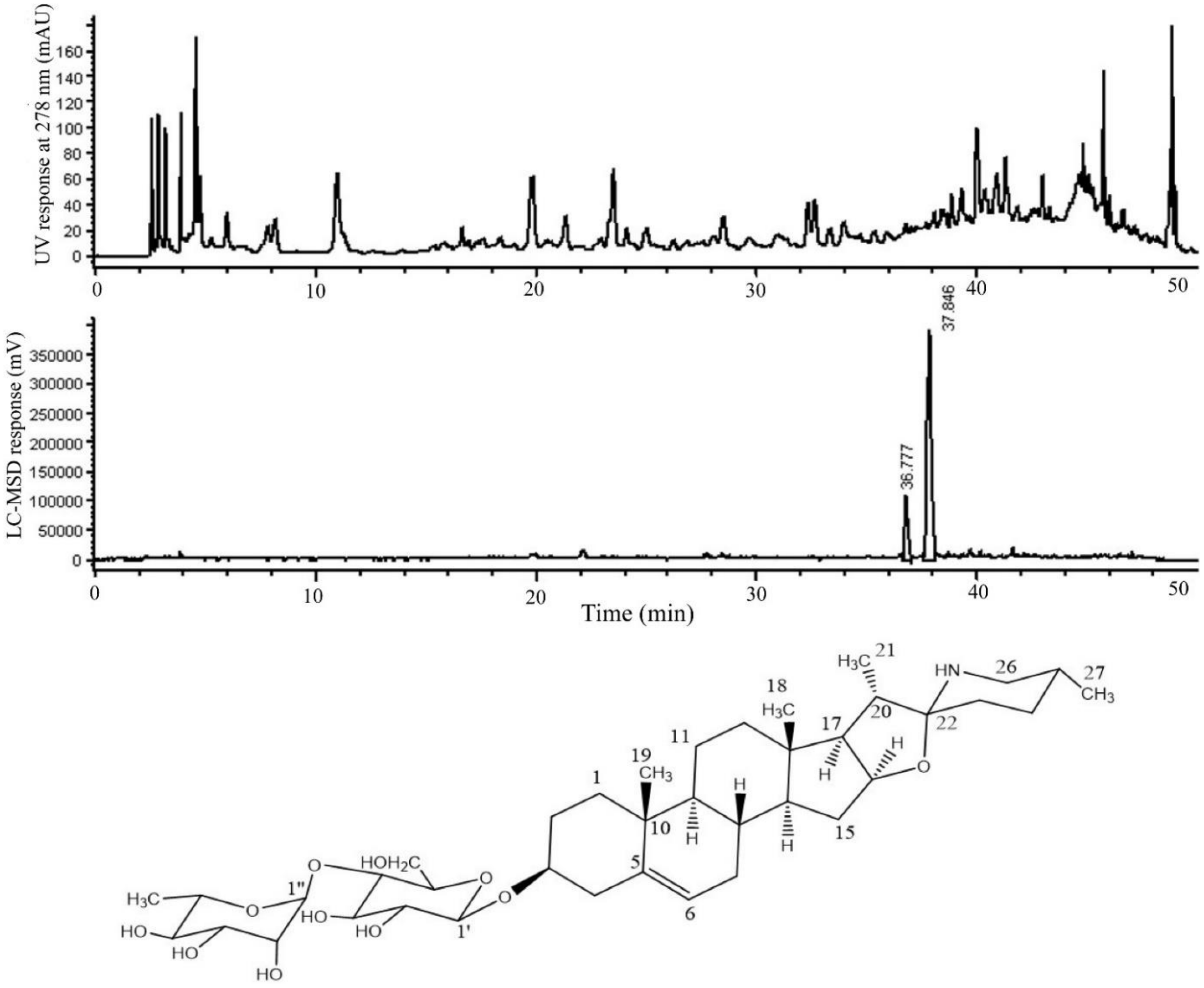

Figure S3. LC-MS analysis of fraction eluted by silica gel column chromatography

UV (top) and SIM (bottom) chromatograms of the extract. The SIM chromatogram reveals the presence of two compounds of nominal mass 721 Da, the mass of khasianine (structure given underneath the chromatograms).

Table S1: ^{13}C NMR δ data (ppm) for khasianine in d_5-pyridine

Atom	Literature[a]	Commercial sample	Commercial sample after addition of DCl	Isolated sample
1	37.4	37.33	37.32	37.36
2	30.2	30.10	30.08	30.11
3	78.3	78.10a	78.08a	78.15
4	39.3	39.19	39.21	39.22
5	140.7	140.73	140.70	140.76
6	121.7	121.67	121.58	121.63
7	32.3a	32.19	31.87	32.05
8	32.5a	31.53	31.54	31.56
9	50.3	50.20	49.89	50.05
10	37.0	36.95	36.89	36.94
11	21.2	21.05	20.88	20.98, br
12	40.1	39.99	38.97	39.46
13	40.6	40.51	40.86	40.75
14	56.7	56.57	56.14	56.35
15	31.6a	32.43	32.27	32.37
16	78.7	78.65	84.09	81.68, x br
17	63.5	63.41	62.22	62.71
18	16.5	16.45	16.05	16.24
19	19.4	19.29	19.28	19.31
20	41.6	41.50	42.32	41.97
21	15.6	15.60	15.73	15.60
22	98.3	98.27	98.38	98.49
23	34.6	34.54	32.82	33.60, br
24	31.1	30.97	28.10c	29.55, v br
25	31.6	31.53	28.46c	29.65, v br
26	48.0	47.96	45.39	46.67, br
27	19.7	19.67	18.46b	19.00
1'	102.7b	102.37	102.38	102.40
2'	75.5	75.47	75.38	75.48
3'	77.0d	76.62	76.52	76.64
4'	78.3	78.11a	78.12a	78.19
5'	76.6d	77.07	77.04	77.06
6'	61.6	61.40	61.29	61.43
1"	102.4b	102.62	102.62	102.63
2"	72.5c	72.57	72.46	72.56
3"	72.8c	72.76	72.63	72.76
4"	73.9	73.94	73.84	73.94
5"	70.3	70.27	70.27	70.30
6"	18.5	18.47	18.50b	18.49

Table S2: ^{1}H NMR data for khasianine in d_5-pyridine

Atom	Commercial sample: exp. δ's (ppm)[a]	Commercial sample + DCl: exp. δ's (ppm)[a]	Isolated sample: exp. δ's (ppm)	Isolated sample: multiplicity, $J_{H,H}$'s (Hz)
1	– ; 0.985b	– ; 0.973b	1.703eq; 0.980ax	ol m; td; $J_{H2ax} = J_{H1eq} = 14.2$, $J_{H2eq} = 3.8$
2	– ; –	– ; –	2.090eq; 1.717ax	ol m; ol m
3	3.900	3.892	3.896	tt; $J_{H2ax} = J_{H4ax} = 11.3$, $J_{H2eq} = J_{H4eq} = 4.6$
4	2.721eq; –	2.719eq; –	2.722eq; 2.464ax	ddd; $J_{H4ax'} = -13.3$, $J_{H3} = 4.5$, $J_{H2eq} = 2.0$; ~t~qt; $J_{H3} \approx J_{H4eq} \approx 12.3$, $J_{H6} \approx J_{H7a} \approx J_{H7b} \approx$ 2.7
6	5.328	5.324	5.329	m
7	– ; –	– ; –	1.839a; 1.465b	ol m; ol m
8	–	–	1.531	td; $J_{H9} = J_{H7ax} = 10.6$, $J_{H7eq} = 4.7$
9	–	–	0.871	ol m
11	– ; –	– ; –	1.430a; 1.375b	ol m; ol m
12	– ; –	– ; –	1.674a; 1.084b	ol m; ol m
14	–	–	1.066	ol m
15	– ; –	– ; –	2.066a; 1.458b	ol m; ol m
16	5.534	4.423	4.983	v br
17	–	–	2.101	ol m
18	0.888	0.820	0.850	3H s
19	0.925	0.907	0.917	3H s
20	–	–	2.179	~qn; $J_{H17} = J_{H21} = 6.9$
21	1.094	1.602	1.359	3H d; $J_{H20} = 7.1$
23	– ; –	– ; –	1.985a; 1.801b	ol m; ol m
24	–	–	1.616	2h ol m
25	–	–	1.957	ol m
26	ol ~2.78a; ol ~2.78b	3.416a, br dd; $J_{H26b} \sim -11$, J_{H25} ~3; 3.011b	3.149a; 2.919b	br d; $J_{H26b} \sim -10$; t; $J_{H26a} = J_{H25} = 11.7$
27	0.827	0.734	0.780	3H d; $J_{H25} = 6.6$
1'	4.983	4.989	4.984	d; $J_{H2'} = 7.7$
2'	4.023	4.017	4.016	dd; $J_{H3'} = 8.9$, $J_{H1'} = 7.9$
3'	ol	ol	4.260	ol m
4'	4.512	4.512	4.498	t; $J_{H5'} = J_{H3'} = 9.4$
5'	3.747	3.762	3.748	ddd; $J_{H4'} = 9.7$, $J_{H6'a} = 2.1$, $J_{H6'b} = 3.3$
6'	ol ; 4.167b	ol ; 4.171b	4.283a; 4.164b	ol m; dd; $J_{H6'a} = -12.2$, $J_{H5'} = 3.6$
1"	5.948	5.951	5.935	d; $J_{H2''} = 1.5$
2"	4.739	4.745	4.738	dd; $J_{H3''} = 3.3$, $J_{H1''} = 1.6$
3"	4.620	4.623	4.617	dd; $J_{H4''} = 9.2$, $J_{H2''} = 3.4$
4"	4.384	4.388	4.382	t; $J_{H5''} = J_{H3''} = 9.4$
5"	5.073	5.067	5.059	dqt; $J_{H4''} = 9.5$, $J_{H6''} = 6.2$
6"	1.761	1.757	1.751	3H d; $J_{H5''} = 6.2$

[a]Selected values only. Legend: br, broad; d, doublet; m, multiplet; ol, overlapped; qn, quintet; qt, quartet; s, singlet; t, triplet; v, very. Geminal couplings assumed to be negative.

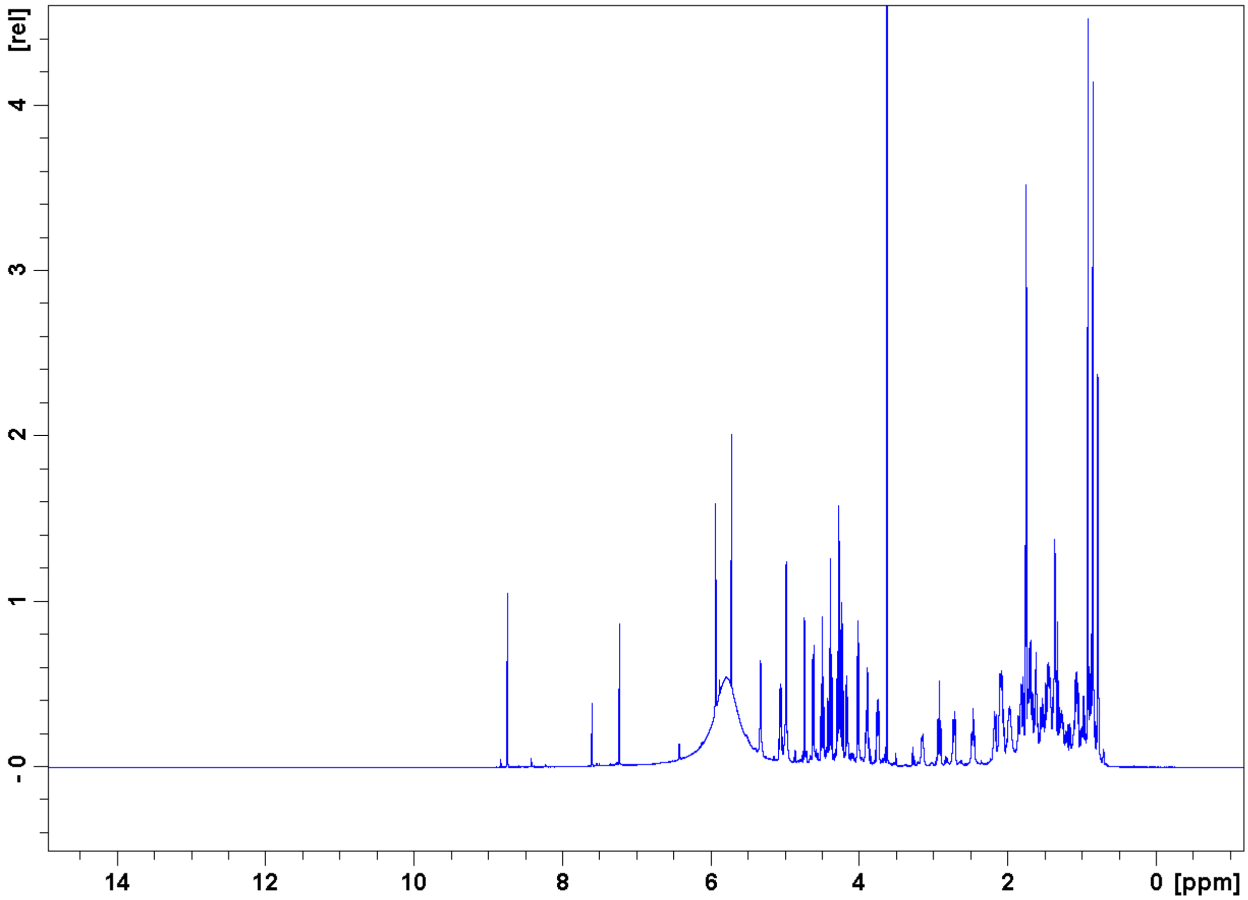

Figure S4. ^{1}H NMR spectrum of khasianine in pyridine-d_5 at 25 °C.

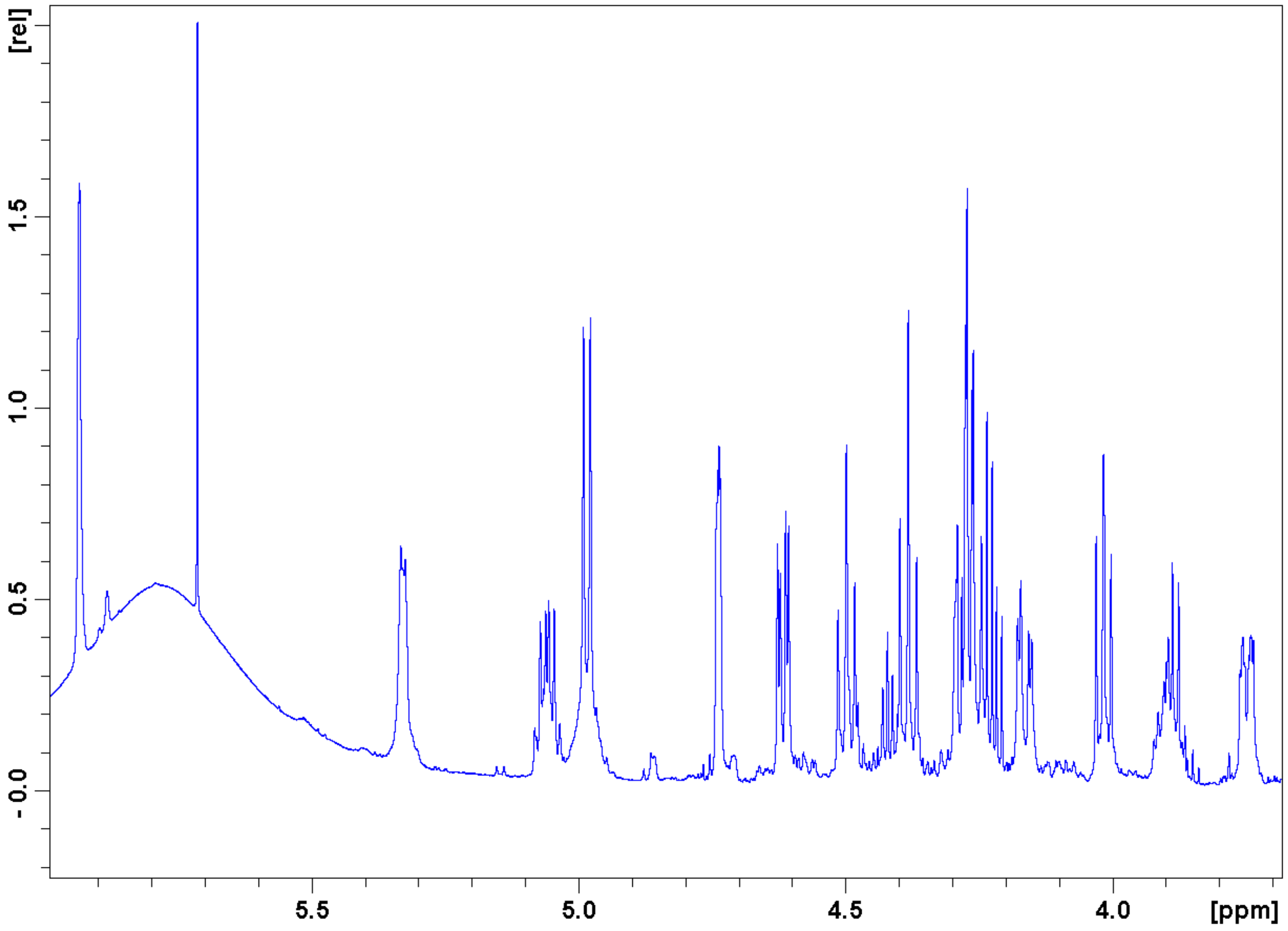

Figure S5. ¹H NMR spectrum of khasianine in pyridine-d_5 at 25 °C.

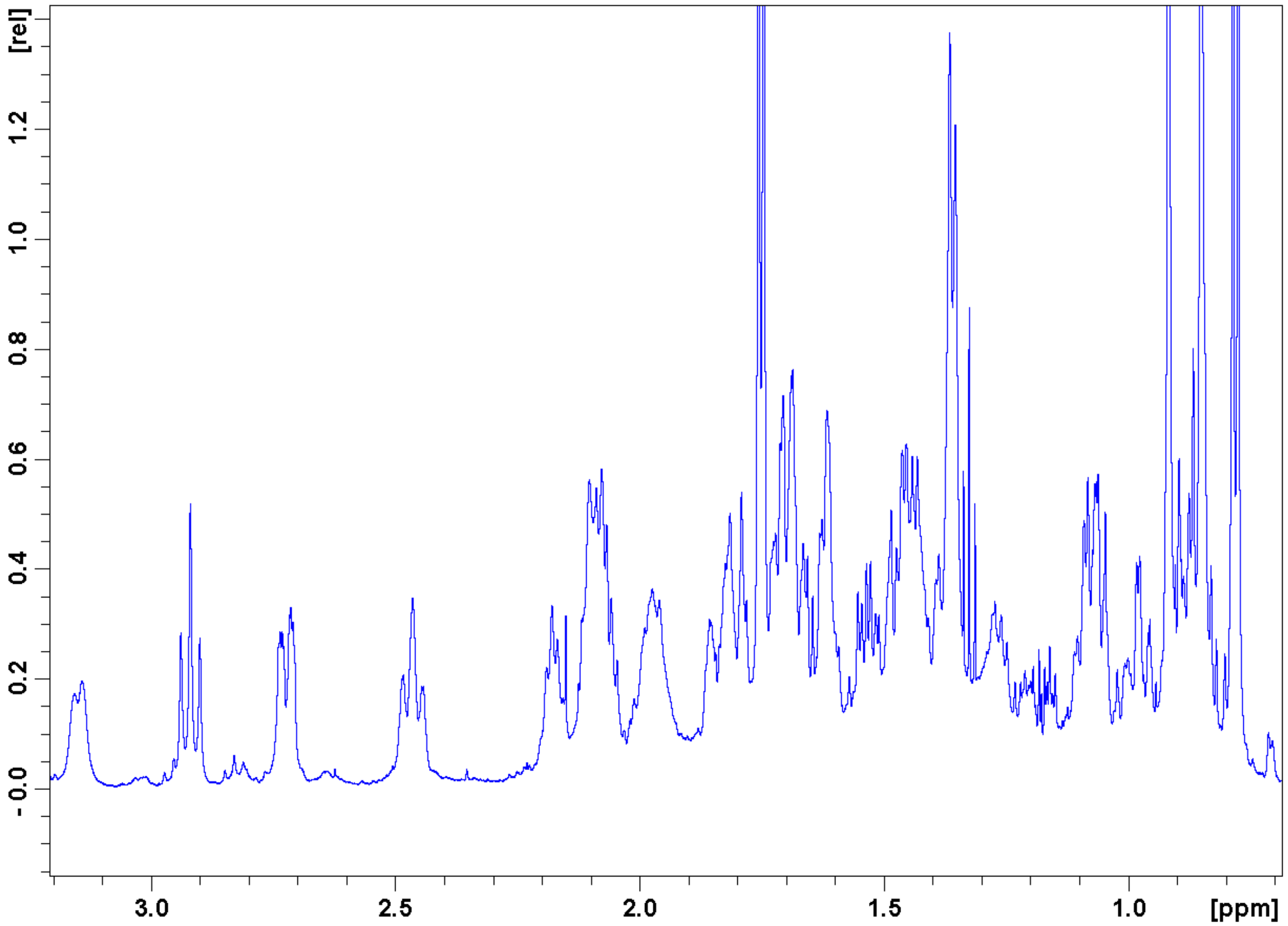

Figure S6. ^{1}H NMR spectrum of khasianine in pyridine-d_5 at 25 °C.

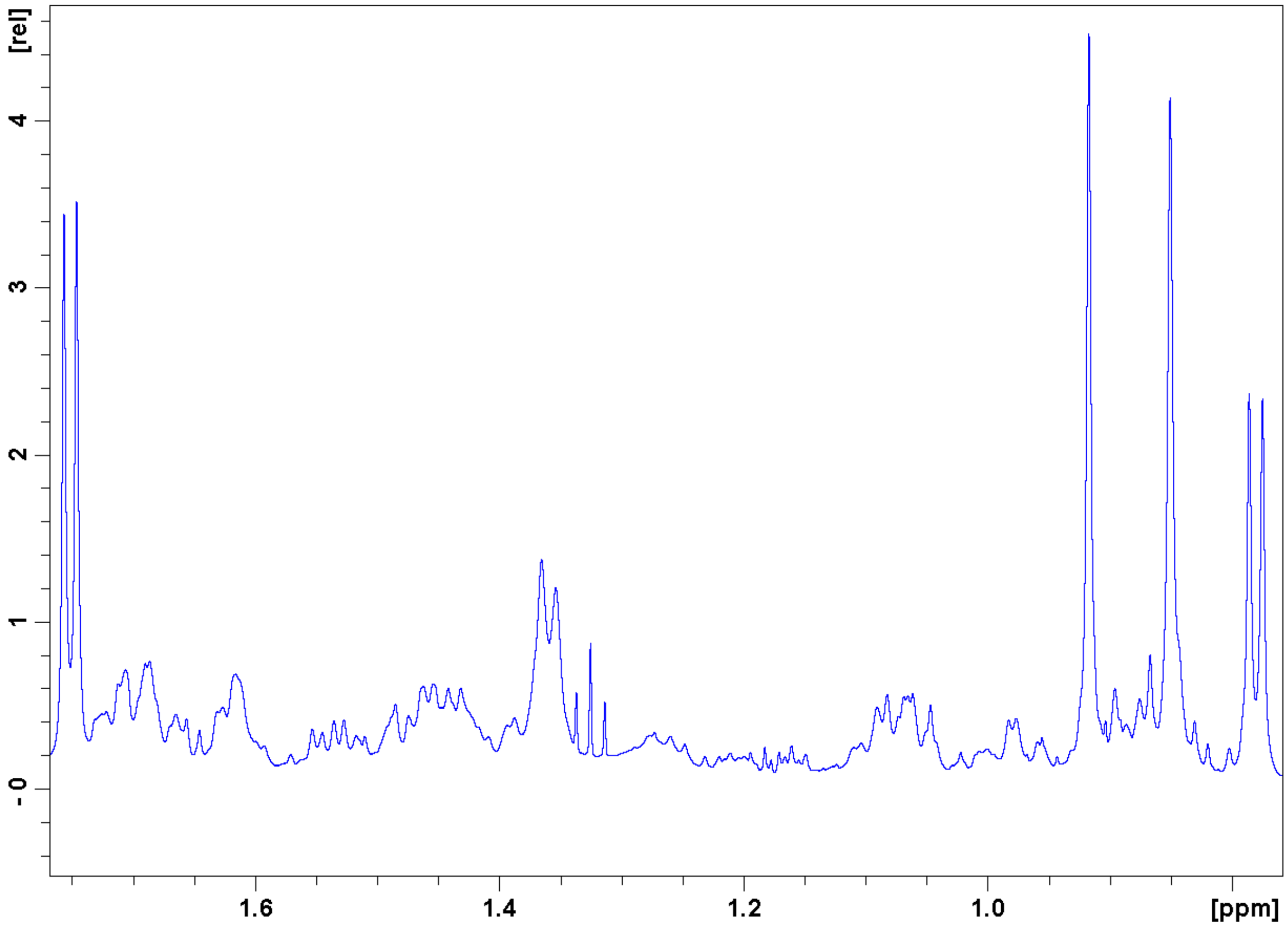

Figure S7. ^{1}H NMR spectrum of khasianine in pyridine-d_5 at 25 °C.

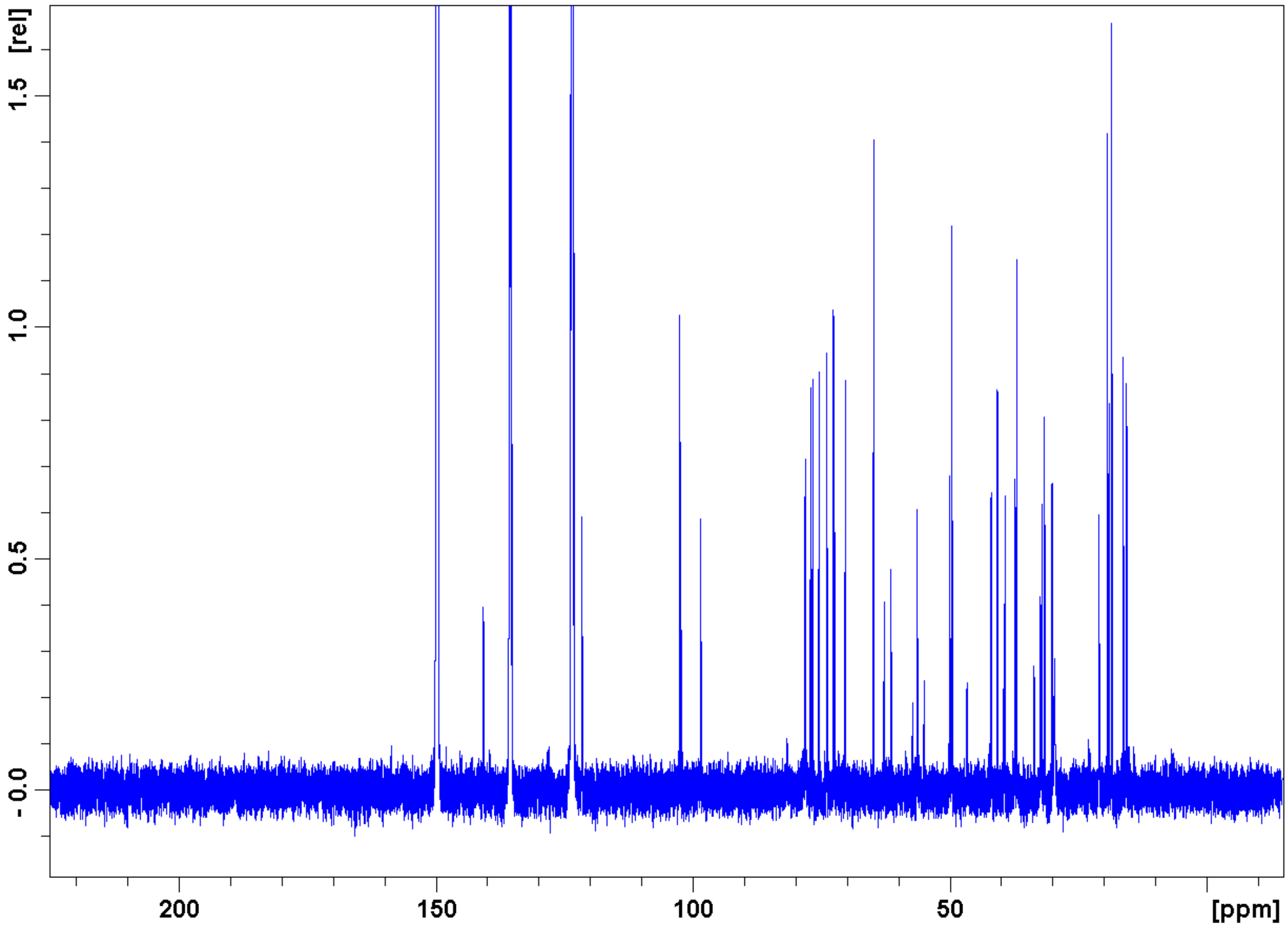

Figure S8. ^{13}C NMR spectrum of khasianine in pyridine-d_5 at 25 °C.

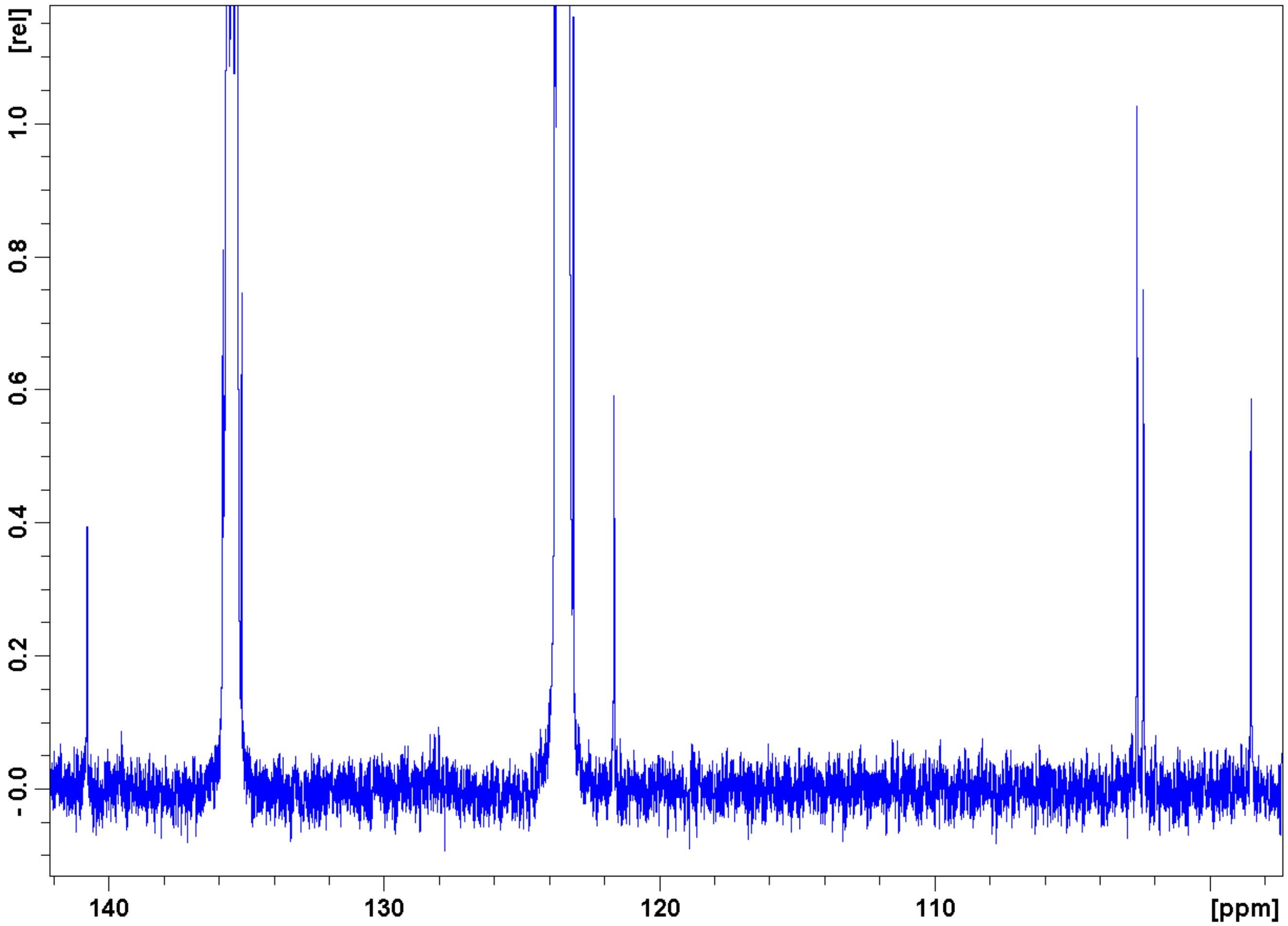

Figure S9. ^{13}C NMR spectrum of khasianine in pyridine-d_5 at 25 °C.

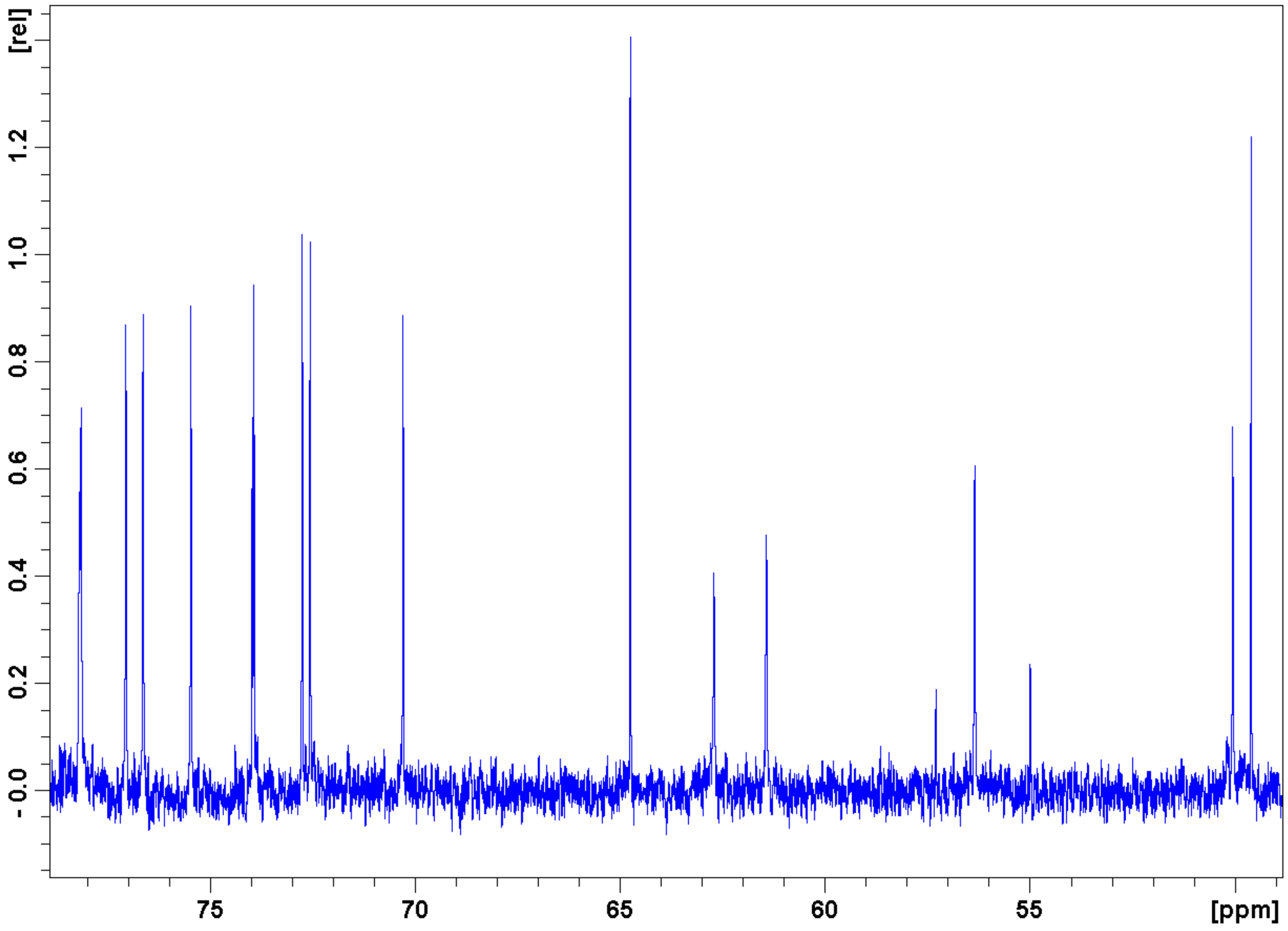

Figure S10. ^{13}C NMR spectrum of khasianine in pyridine-d_5 at 25 °C.

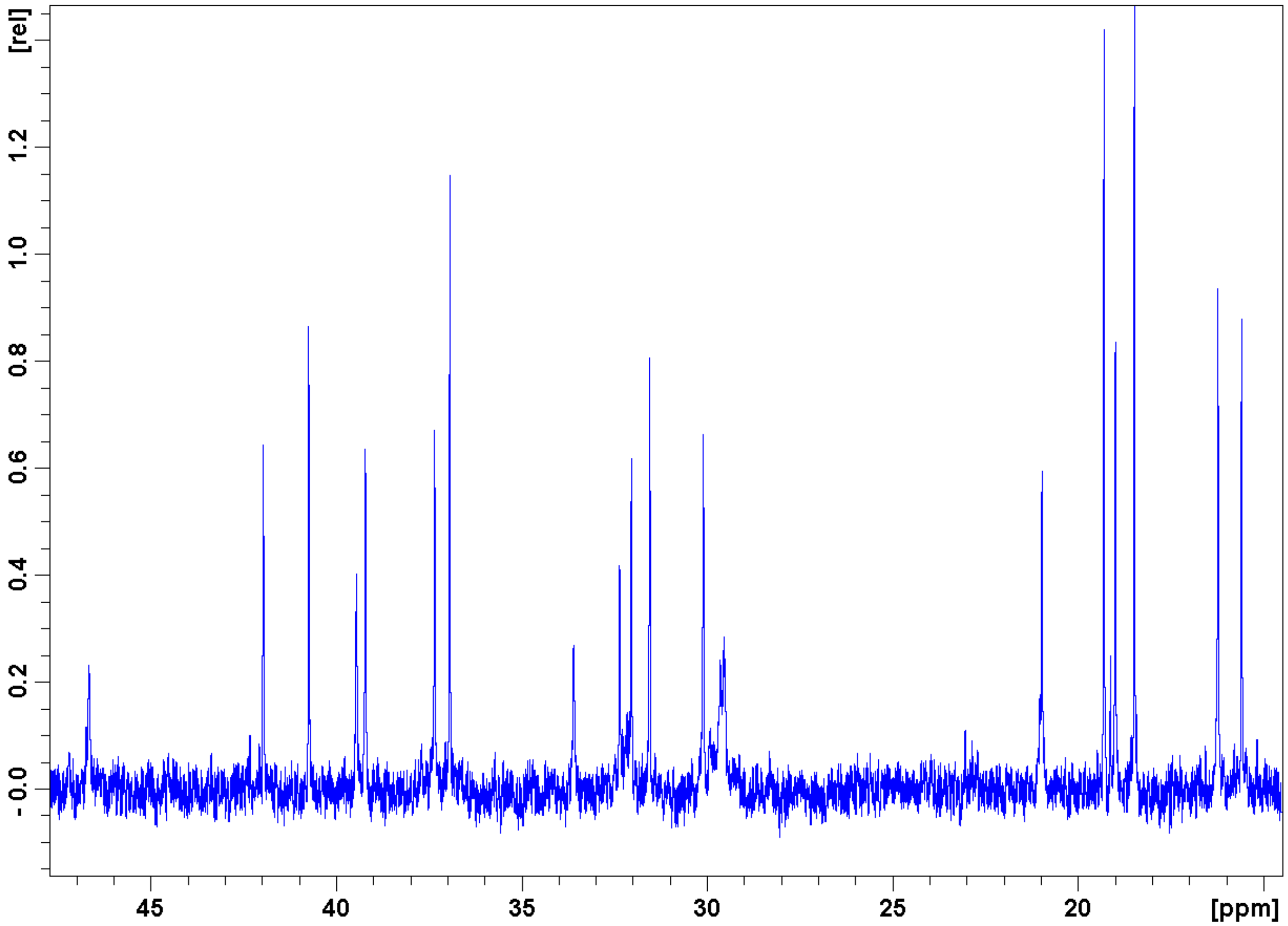

Figure S11. ^{13}C NMR spectrum of khasianine in pyridine-d_5 at 25 °C.

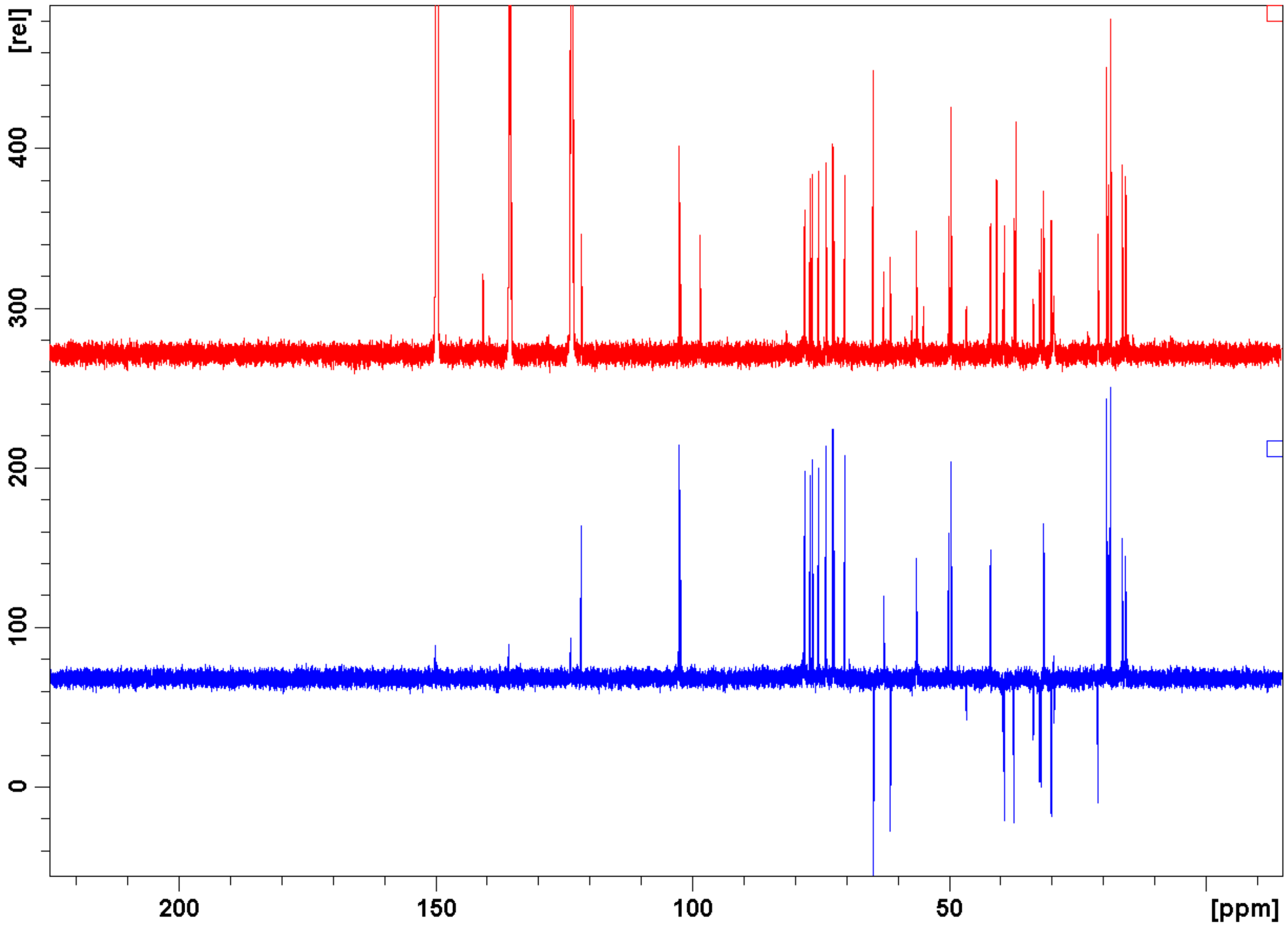

Figure S12. DEPT135 NMR spectrum of khasianine in pyridine-d_5 at 25 °C.

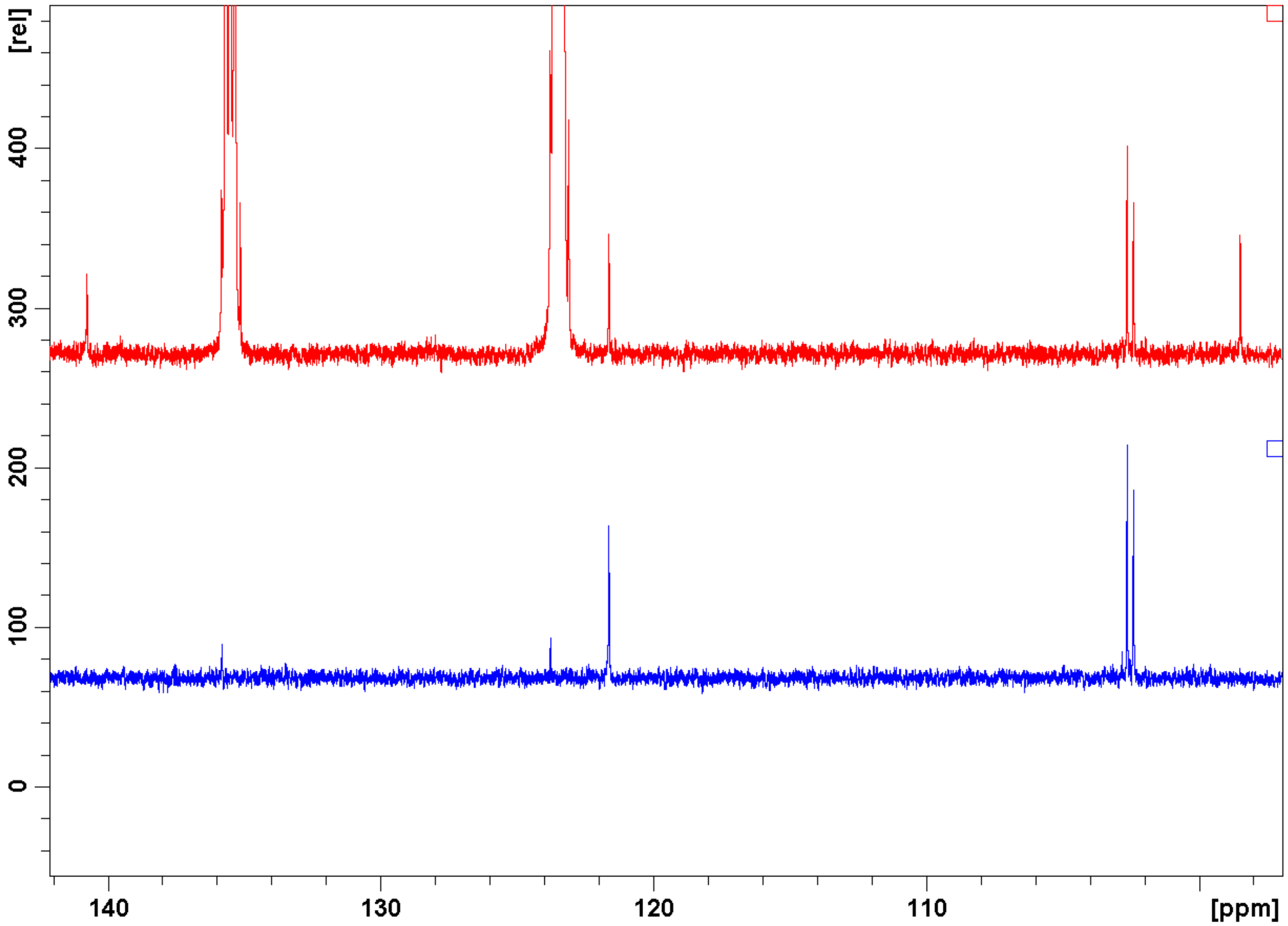

Figure S13. DEPT135 NMR spectrum of khasianine in pyridine-d_5 at 25 °C.

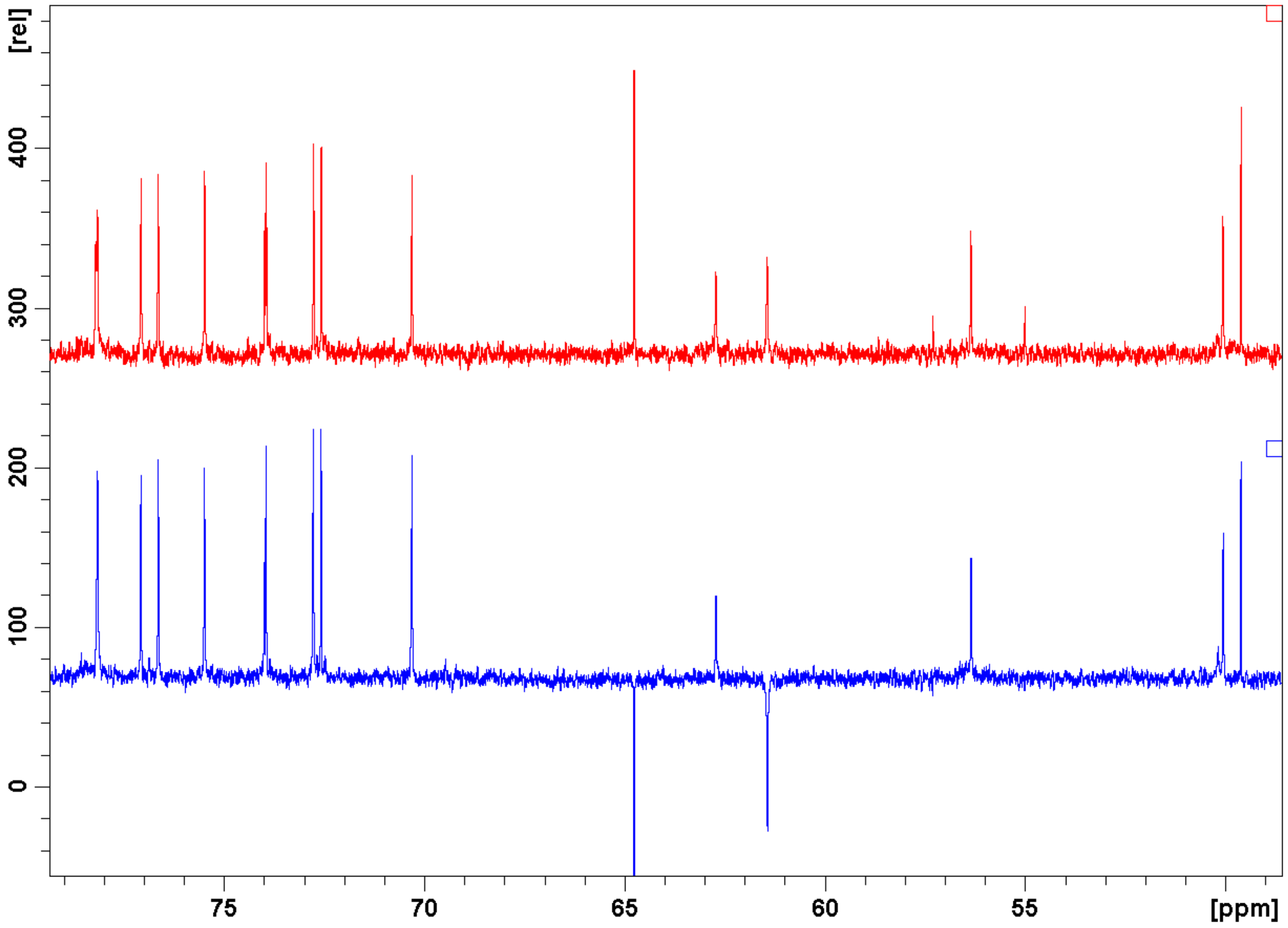

Figure S14. DEPT135 NMR spectrum of khasianine in pyridine-d_5 at 25 °C.

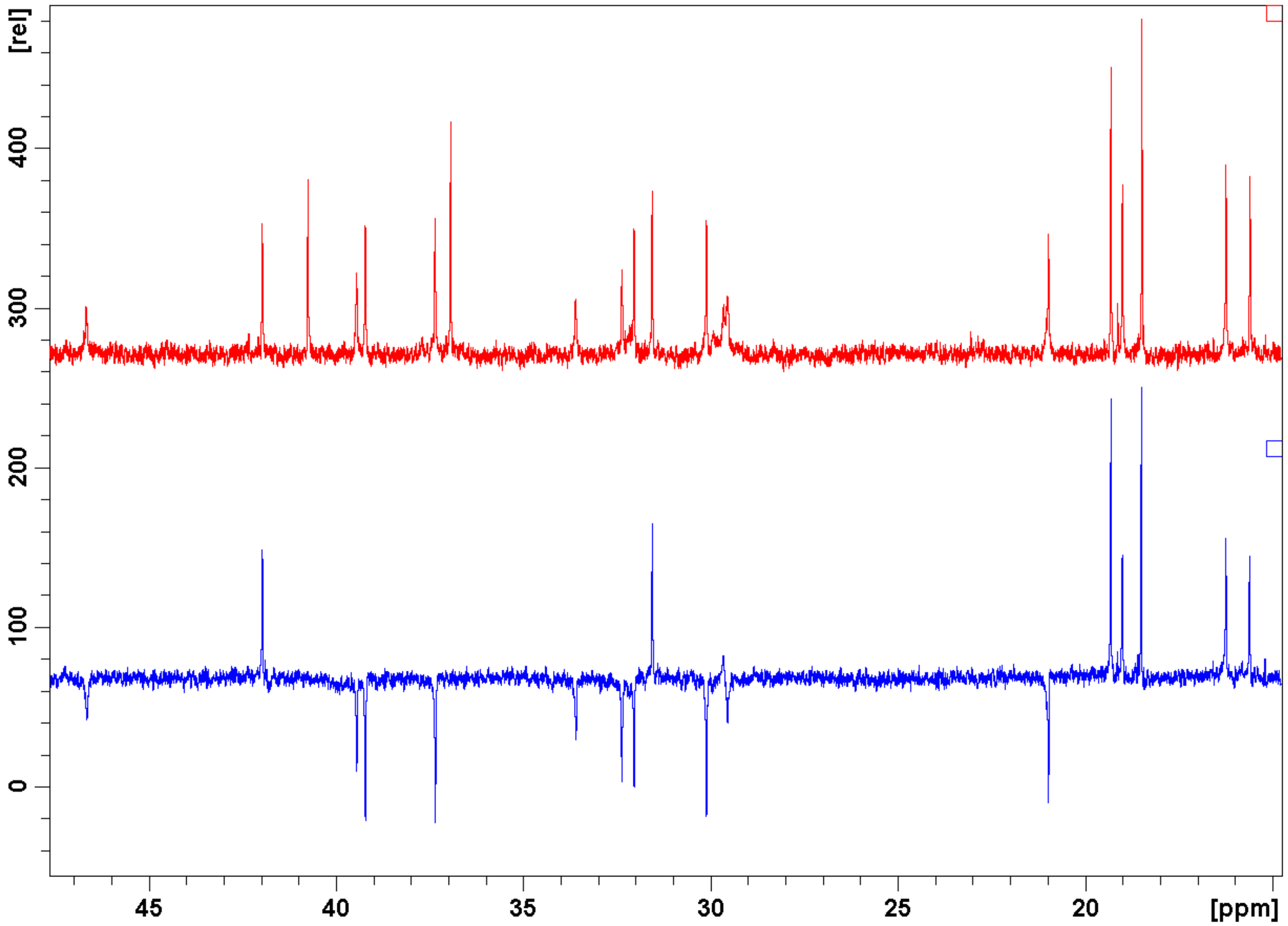

Figure S15. DEPT135 NMR spectrum of khasianine in pyridine-d_5 at 25 °C.

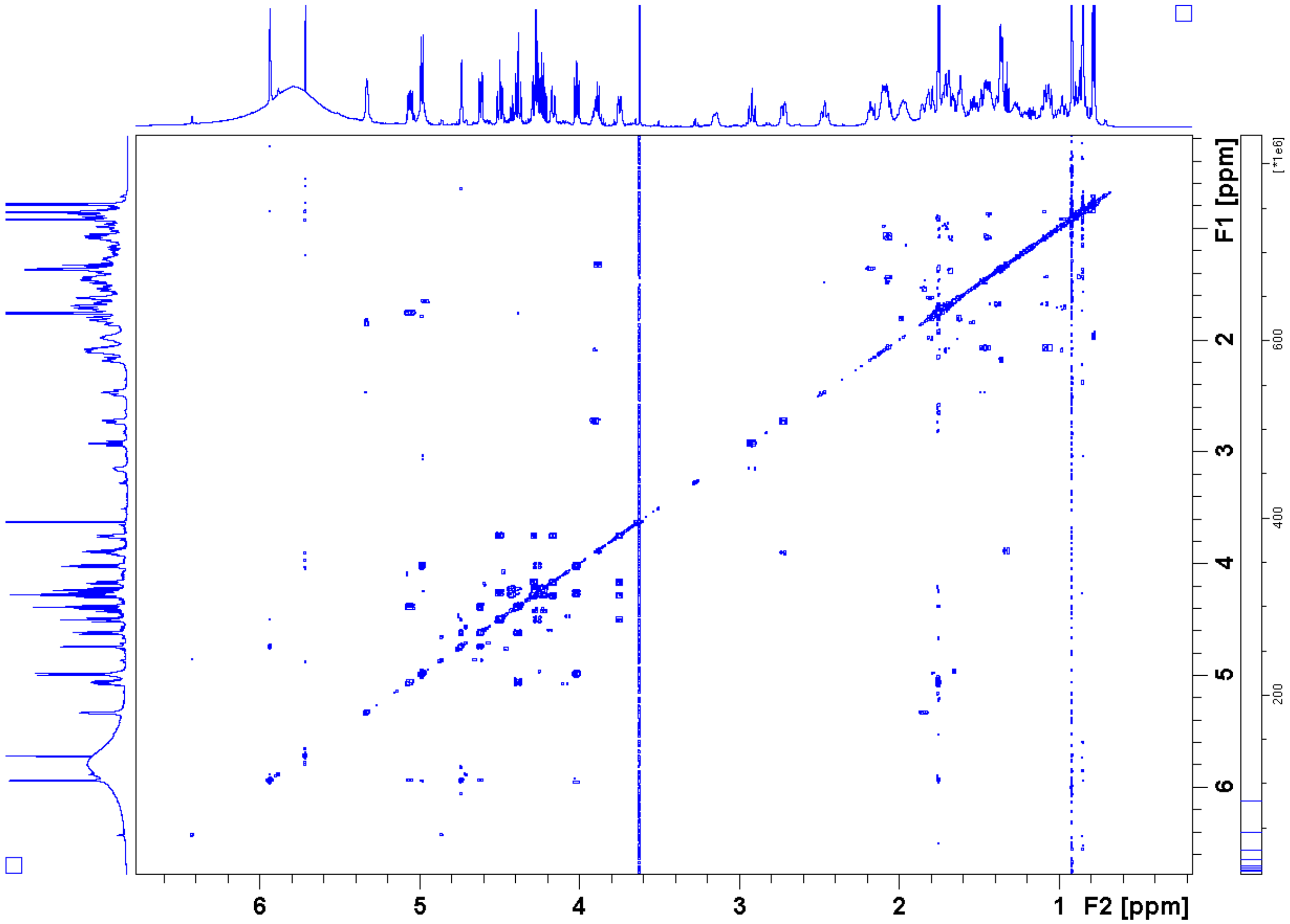

Figure S16. ^{1}H–^{1}H COSY NMR spectrum of khasianine in pyridine-d_5 at 25 °C.

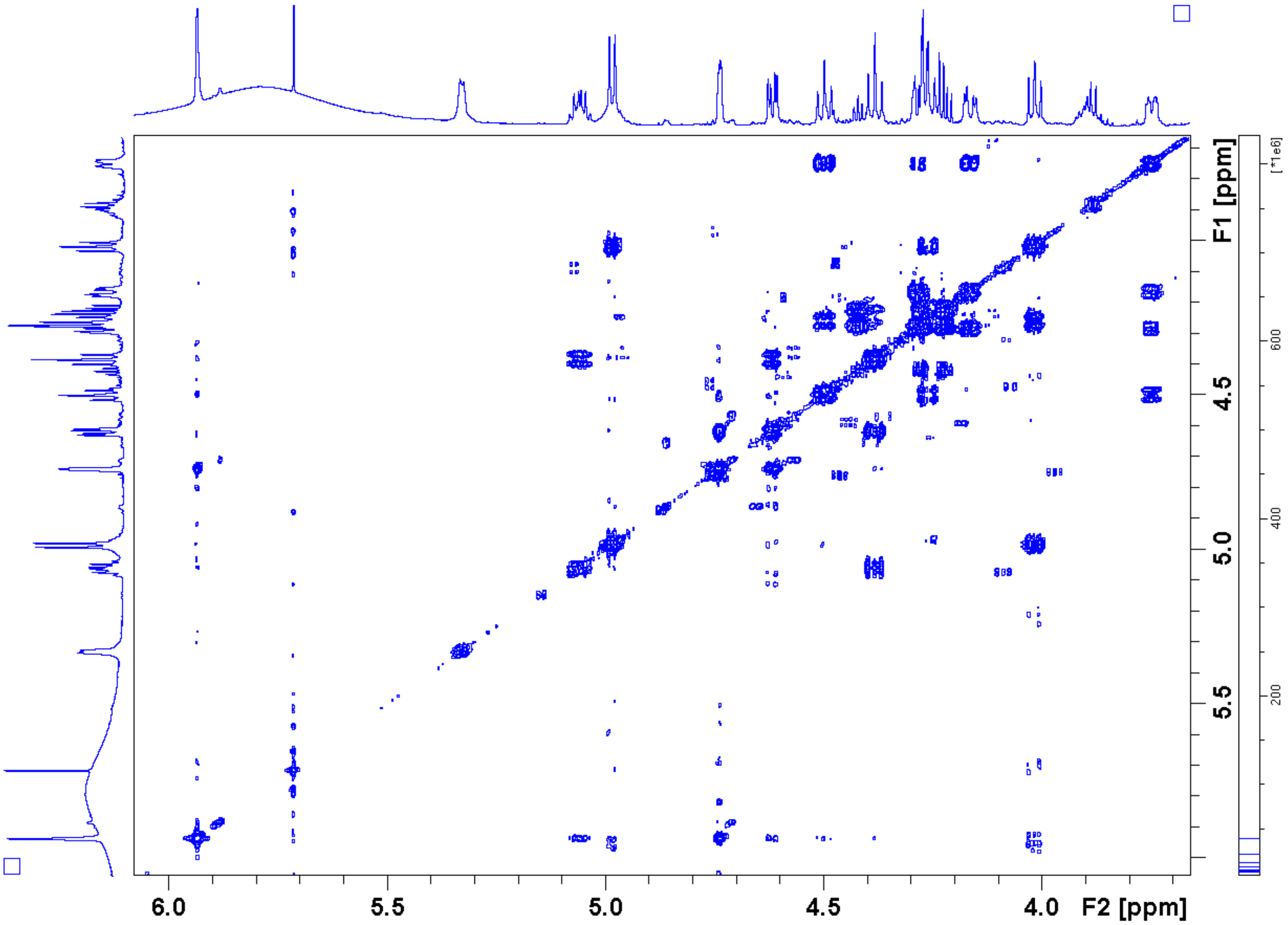

Figure S17. ^{1}H–^{1}H COSY NMR spectrum of khasianine in pyridine-d_5 at 25 °C.

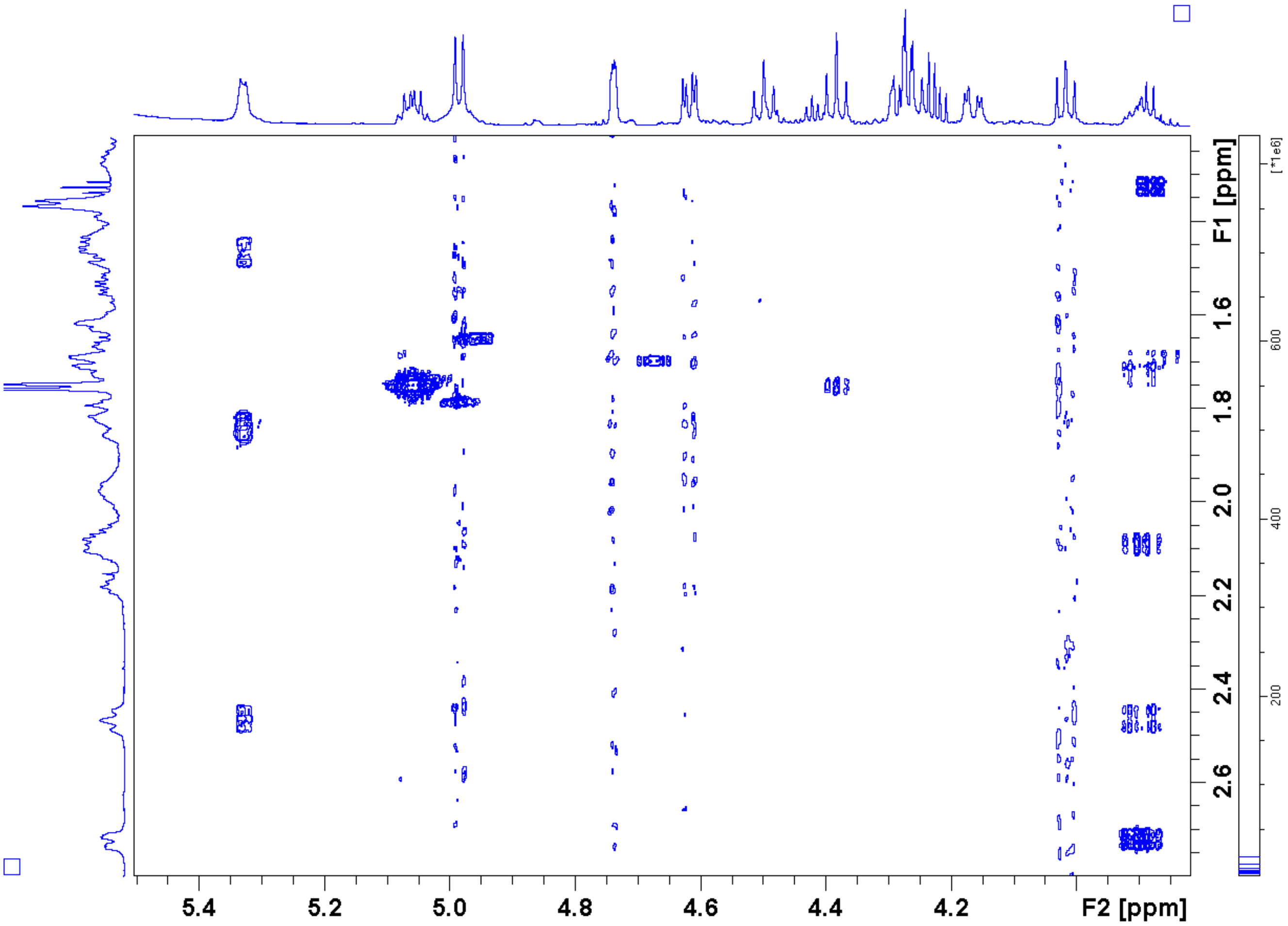

Figure S18. ^{1}H–^{1}H COSY NMR spectrum of khasianine in pyridine-d_5 at 25 °C.

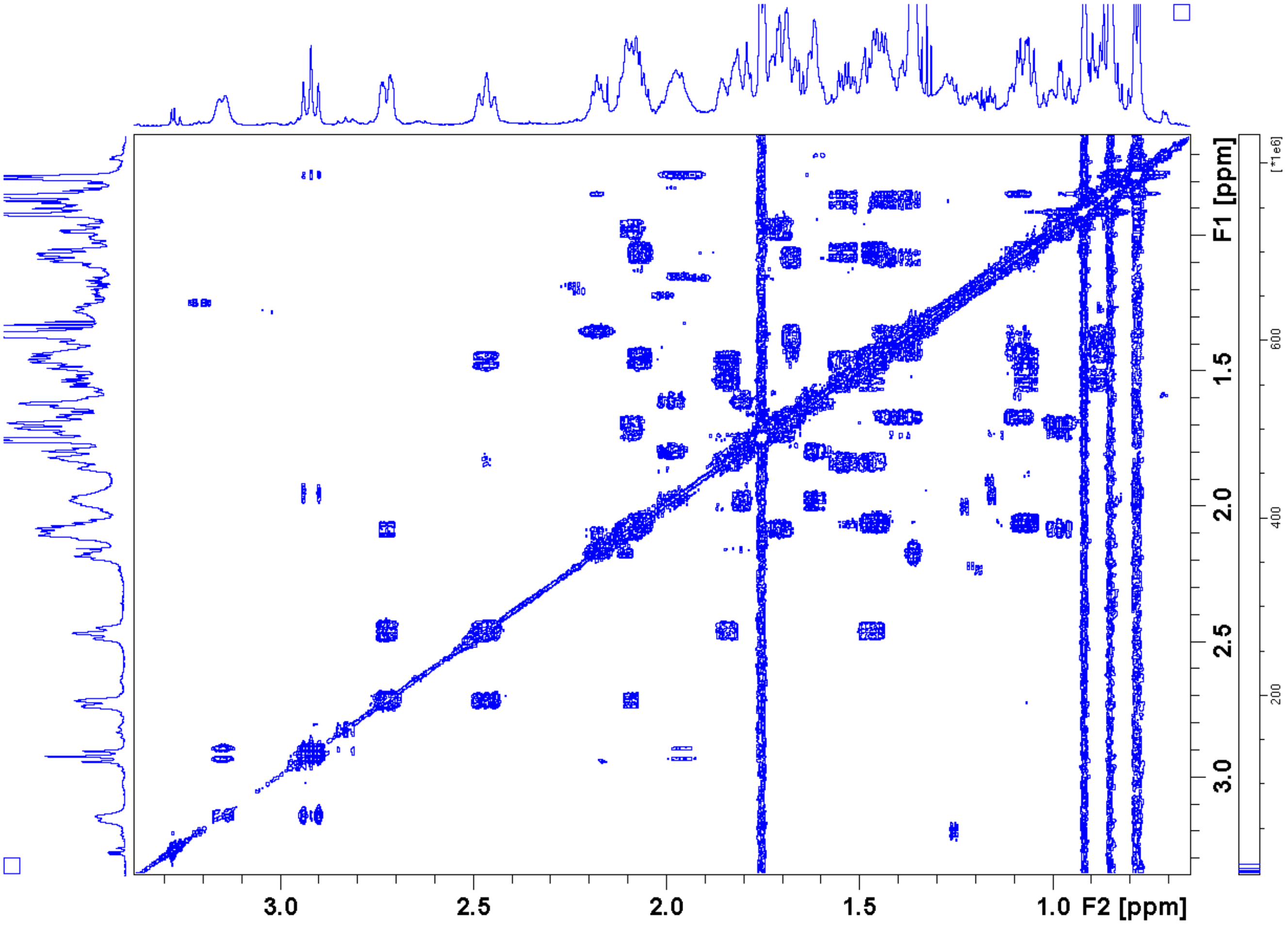

Figure S19. ^{1}H–^{1}H COSY NMR spectrum of khasianine in pyridine-d_5 at 25 °C.

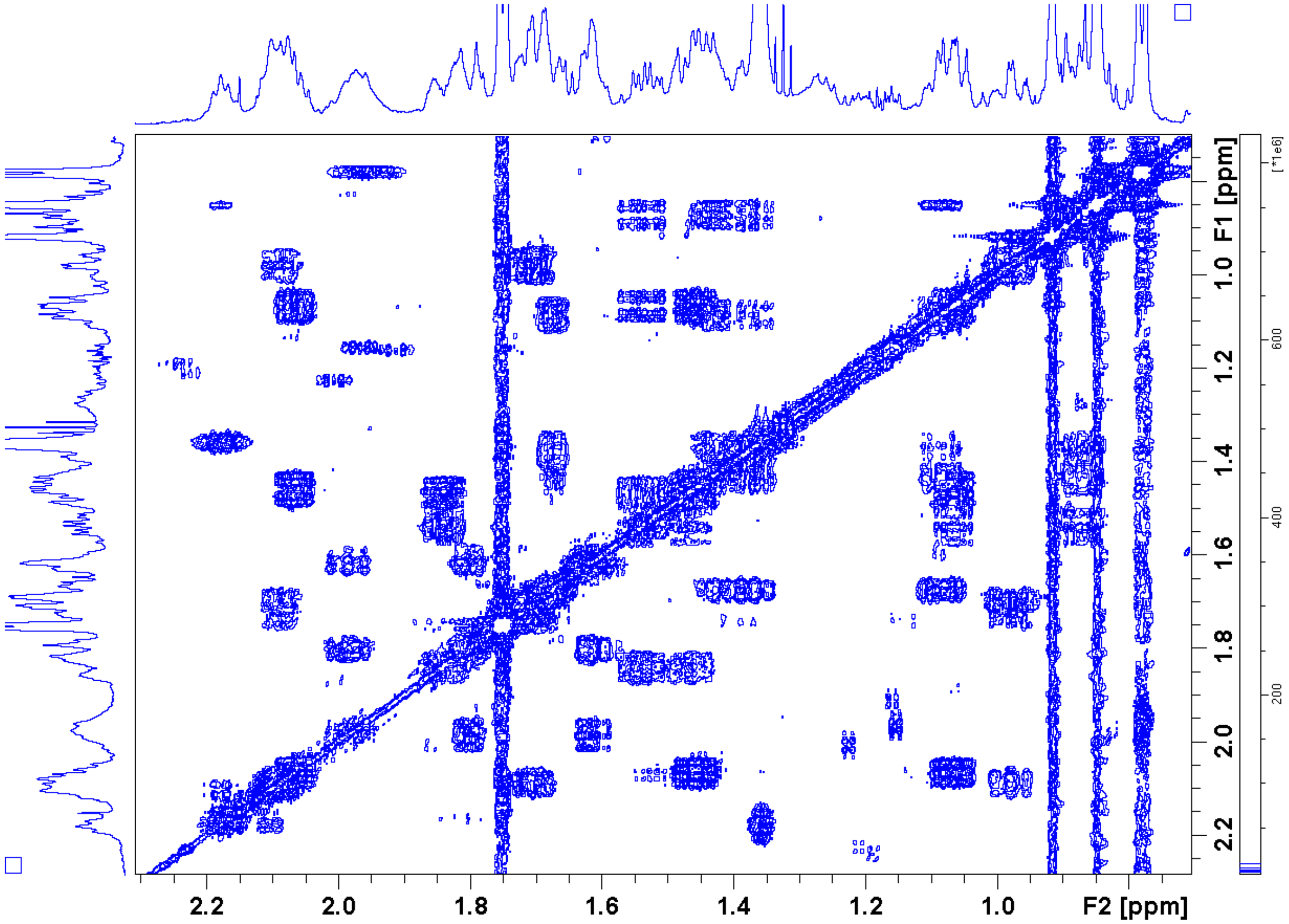

Figure S20. ^{1}H–^{1}H COSY NMR spectrum of khasianine in pyridine-d_5 at 25 °C.

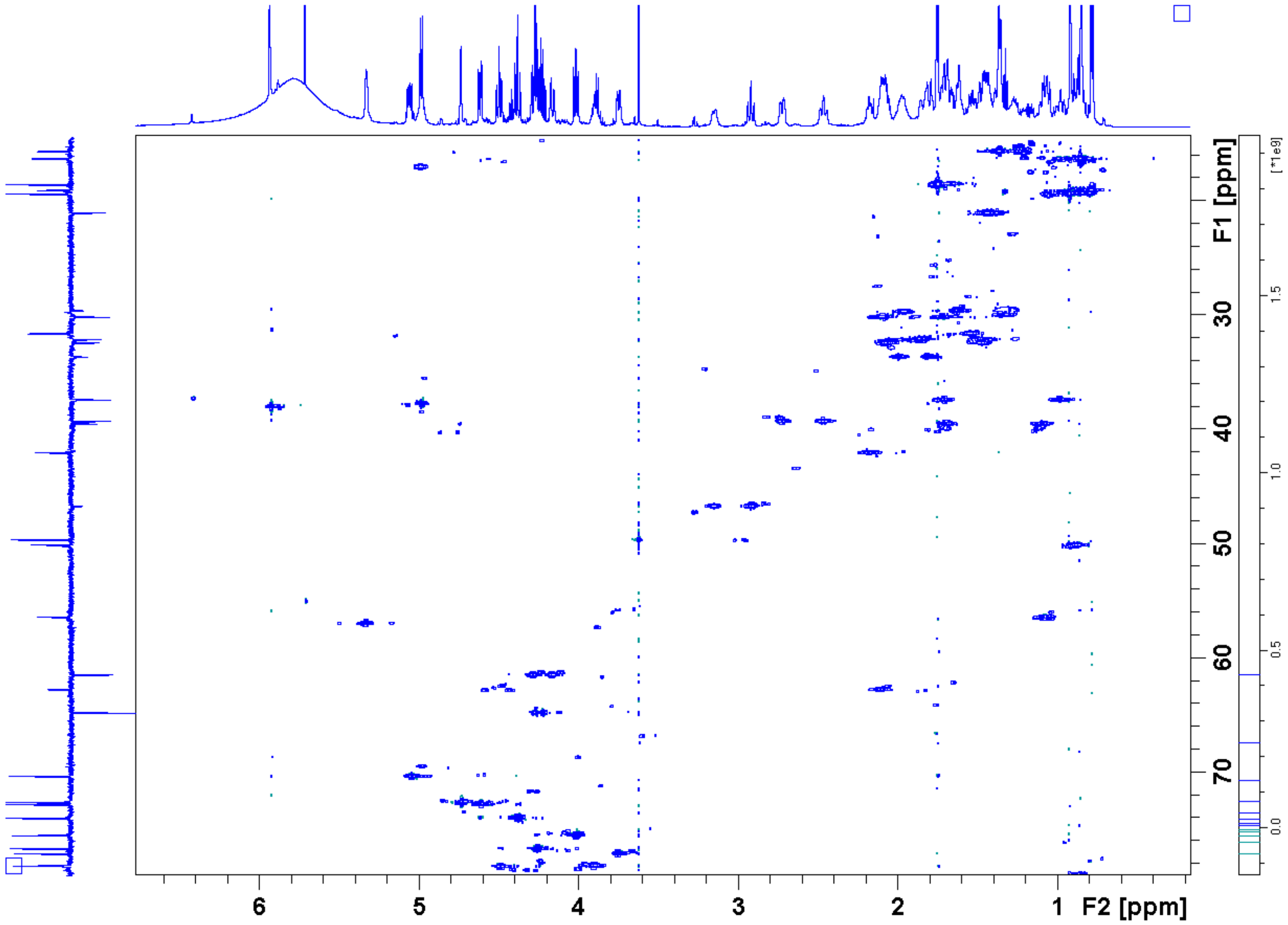

Figure S21. ^{1}H–^{13}C HSQC NMR spectrum of khasianine in pyridine-d_5 at 25 °C.

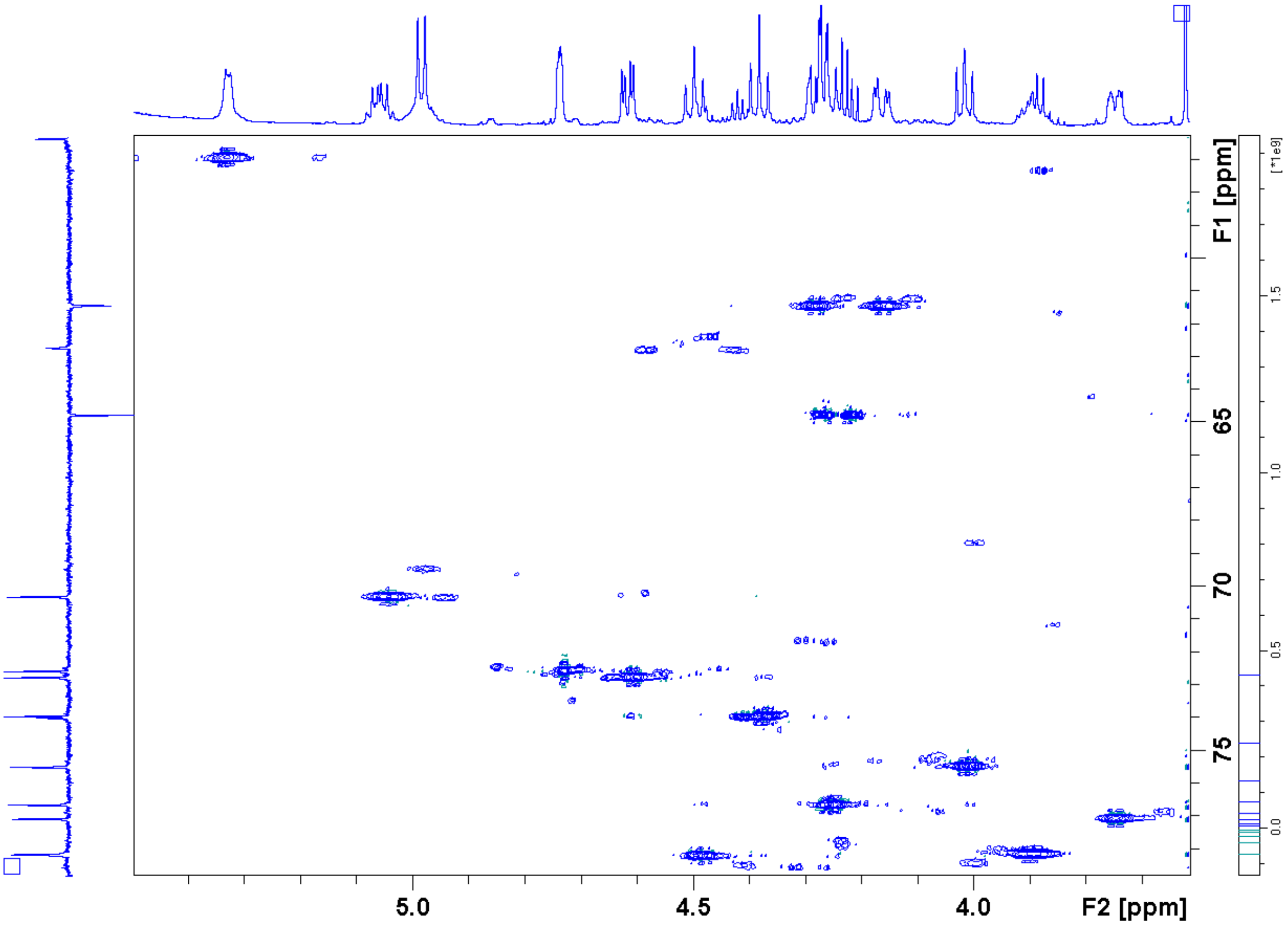

Figure S22. ^{1}H–^{13}C HSQC NMR spectrum of khasianine in pyridine-d_5 at 25 °C.

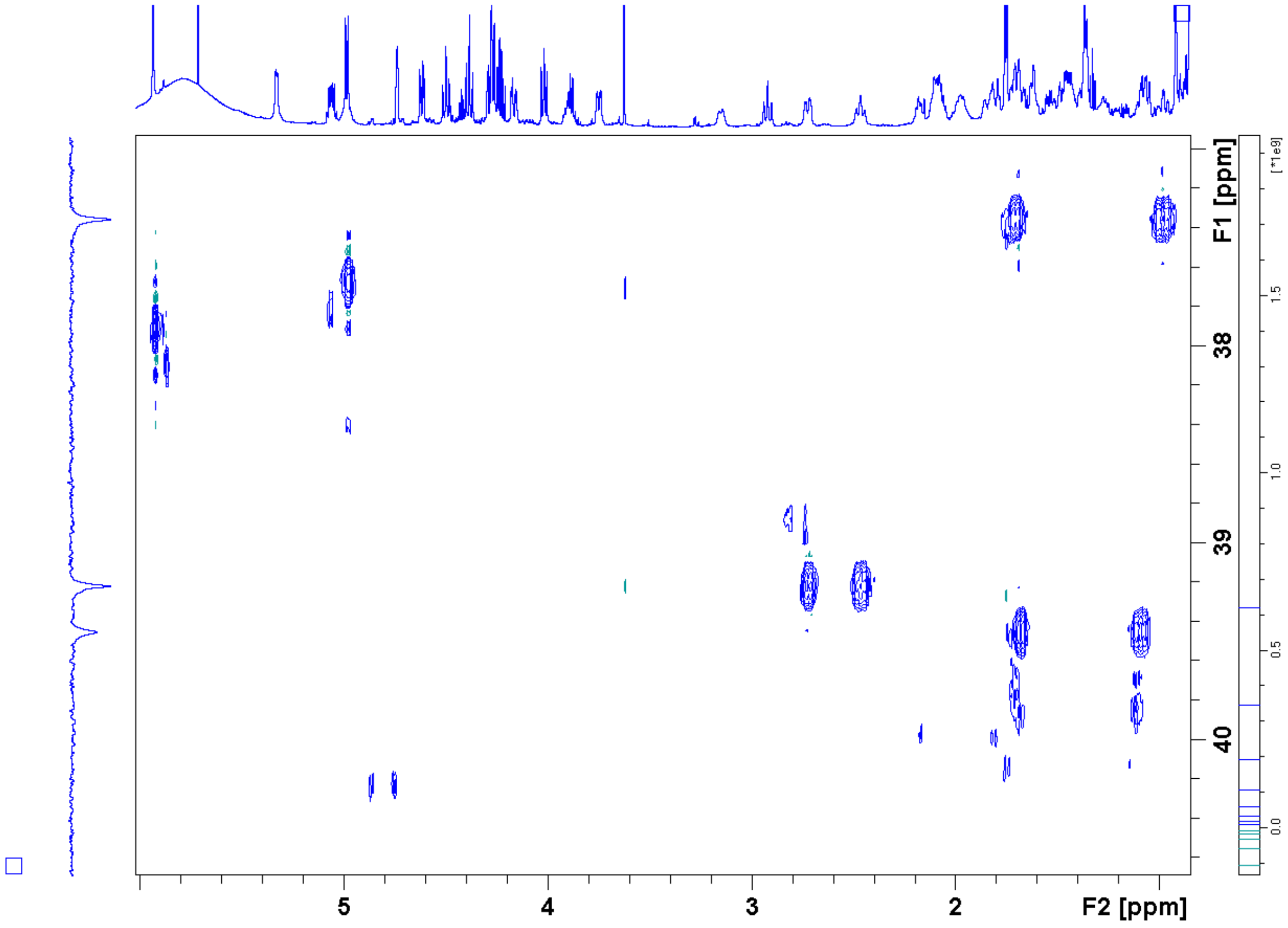

Figure S23. ^{1}H–^{13}C HSQC NMR spectrum of khasianine in pyridine-d_5 at 25 °C.

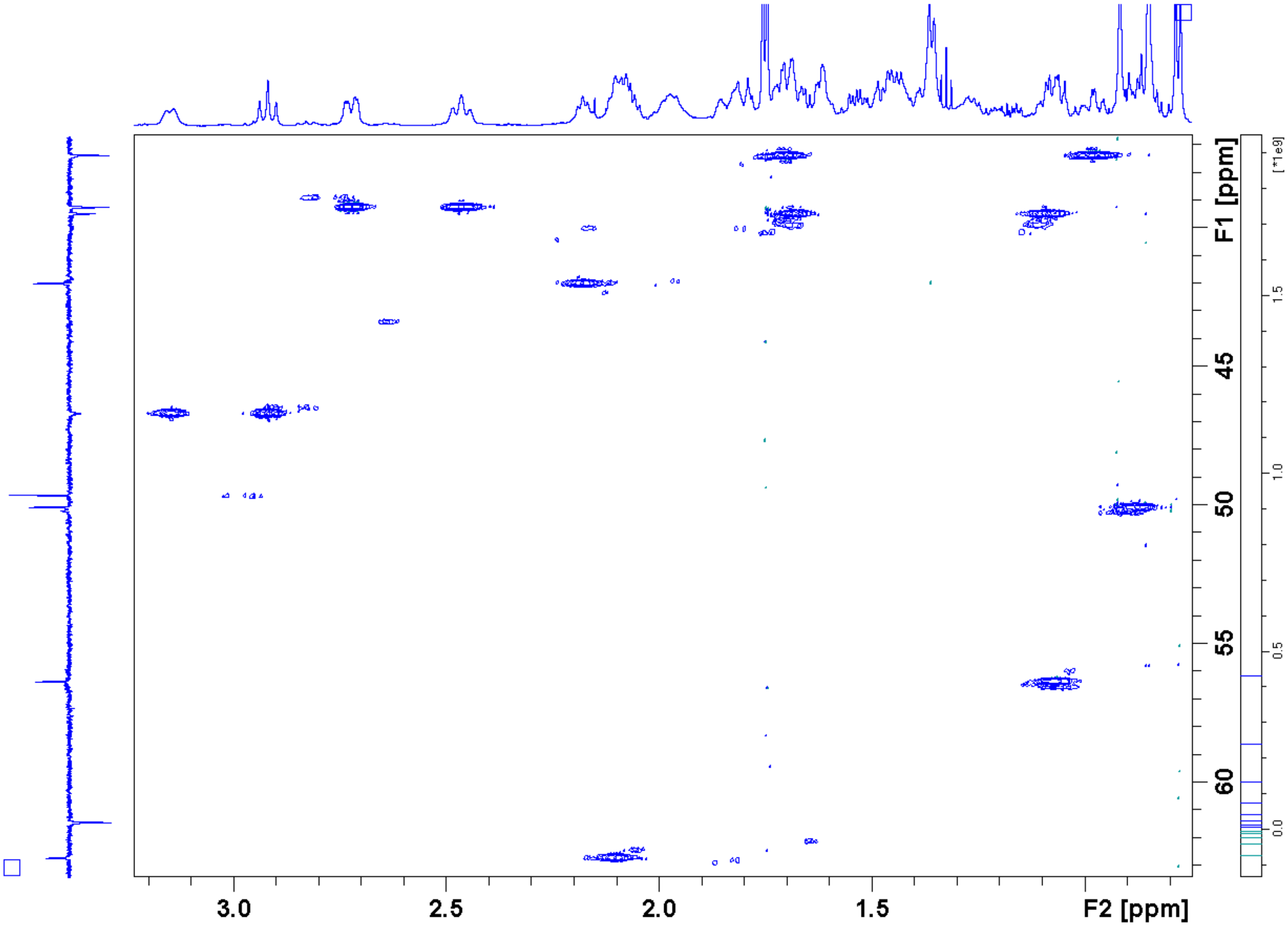

Figure S24. ^{1}H–^{13}C HSQC NMR spectrum of khasianine in pyridine-d_5 at 25 °C.

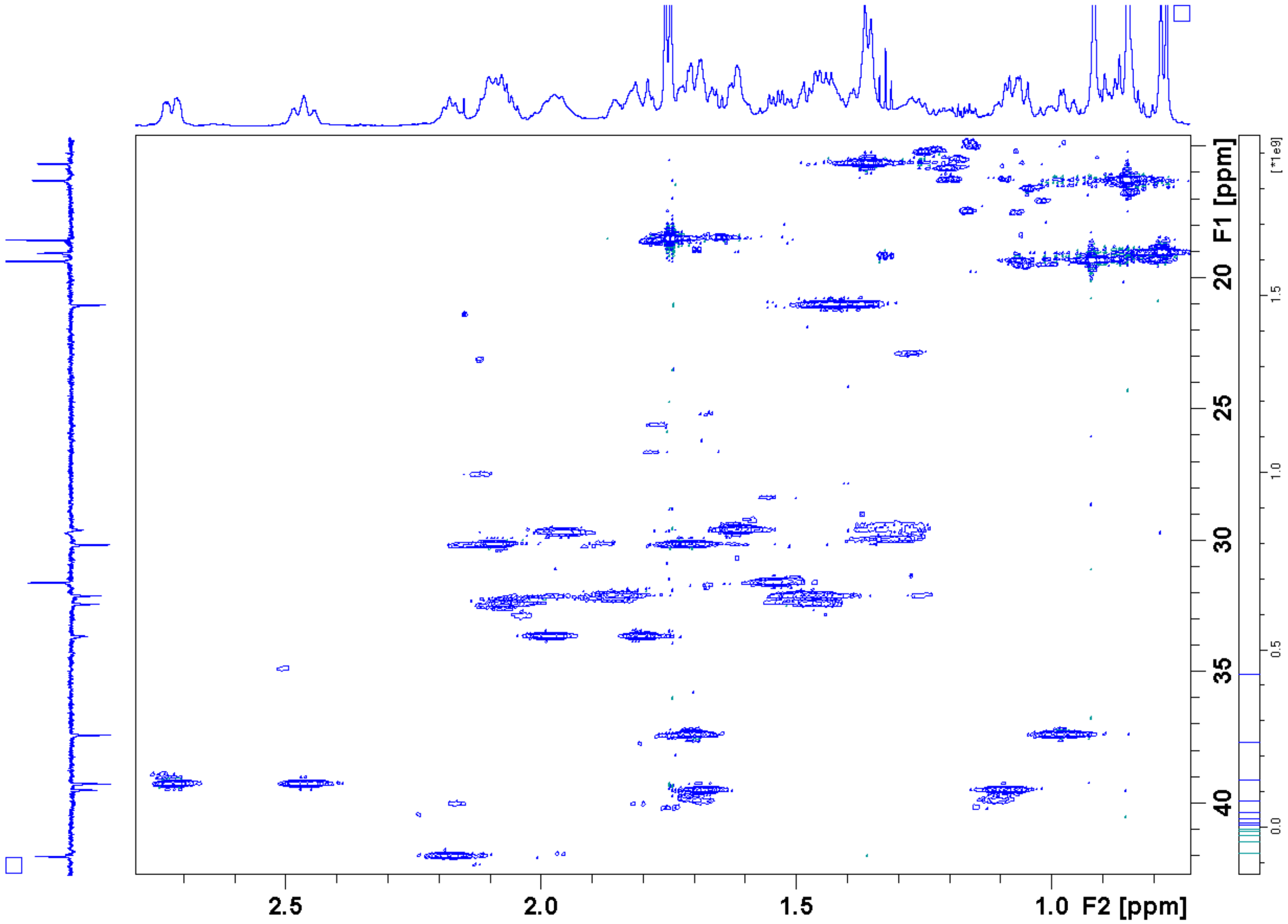

Figure S25. ^{1}H–^{13}C HSQC NMR spectrum of khasianine in pyridine-d_5 at 25 °C.

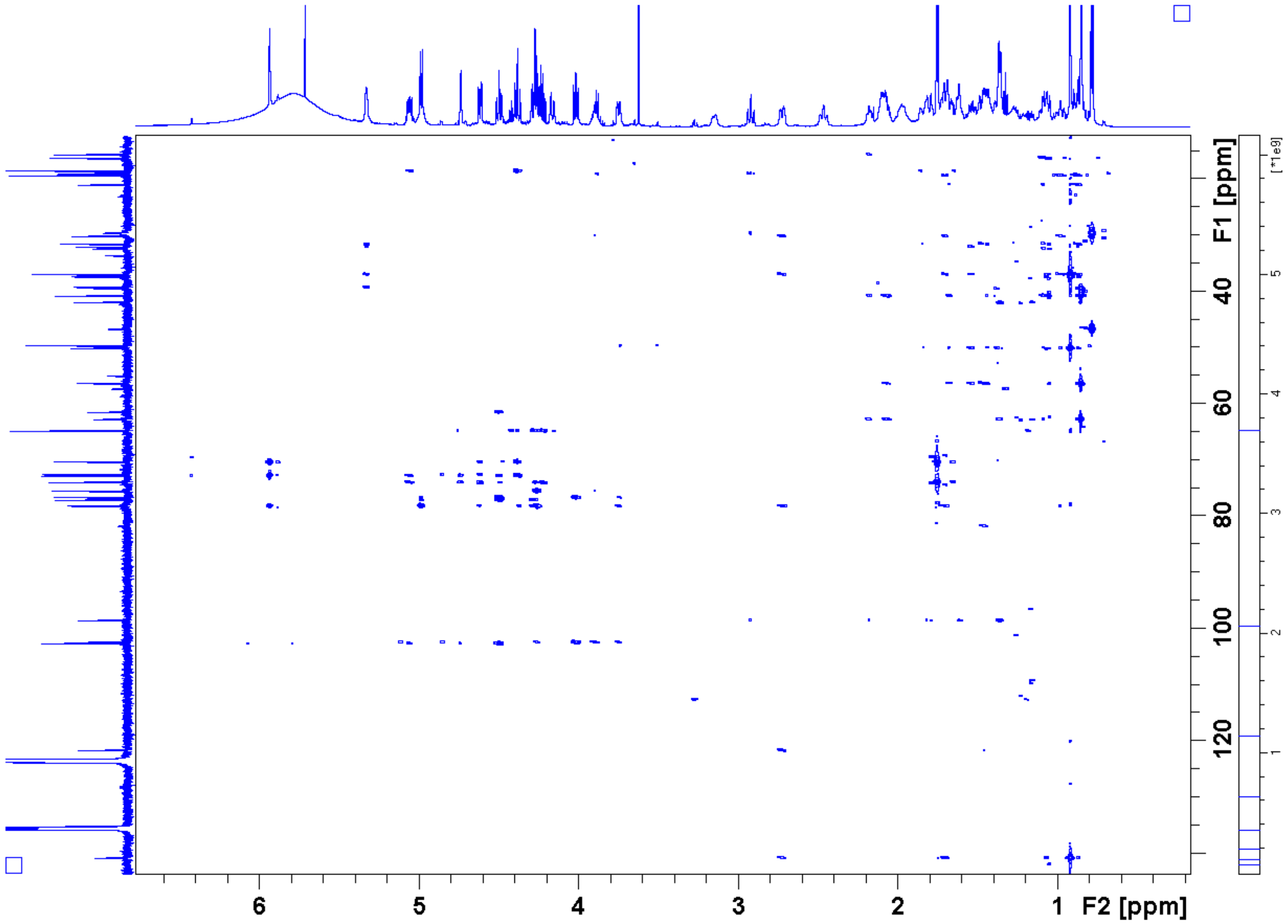

Figure S26. ^{1}H–^{13}C HMBC NMR spectrum of khasianine in pyridine-d_5 at 25 °C.

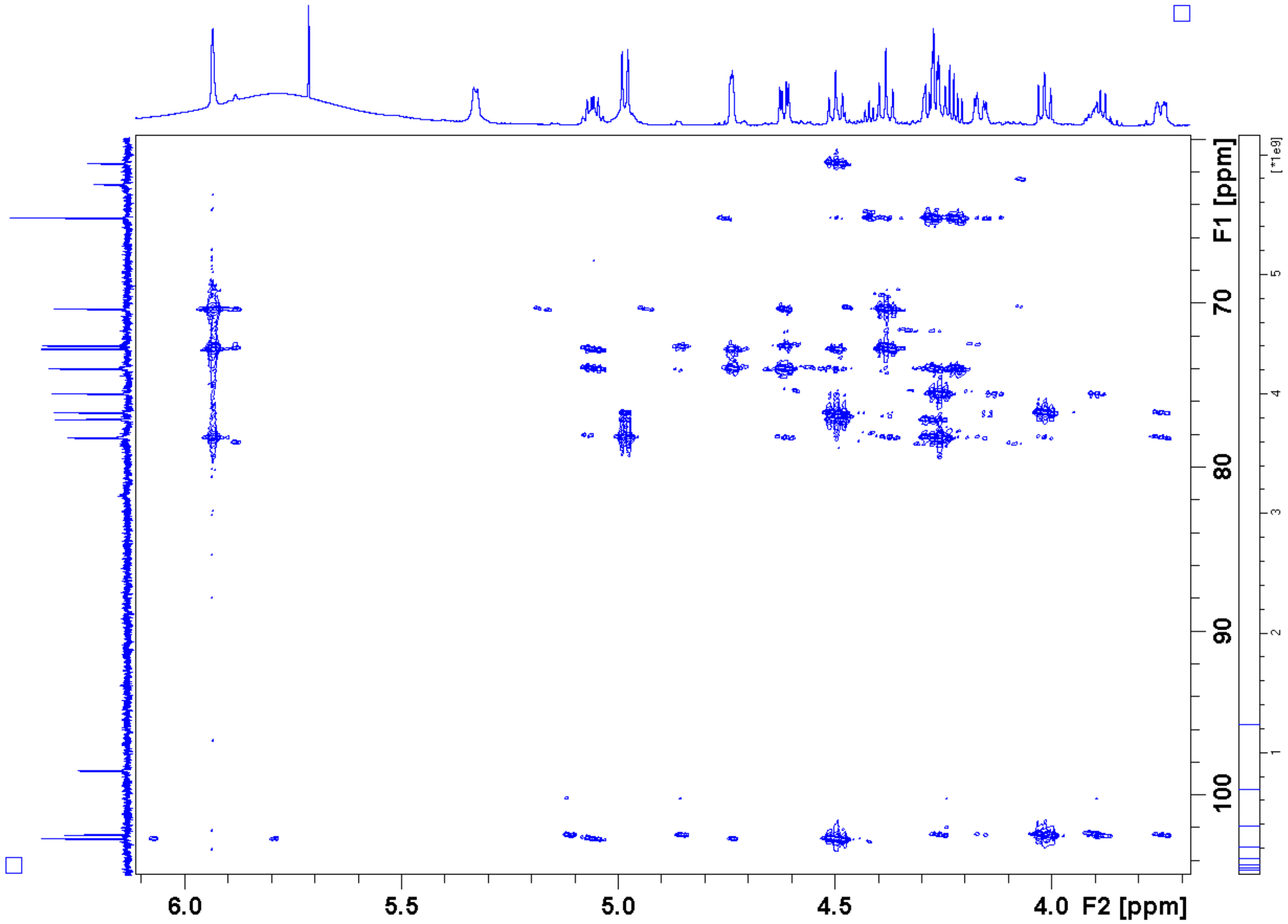

Figure S27. ^{1}H–^{13}C HMBC NMR spectrum of khasianine in pyridine-d_5 at 25 °C.

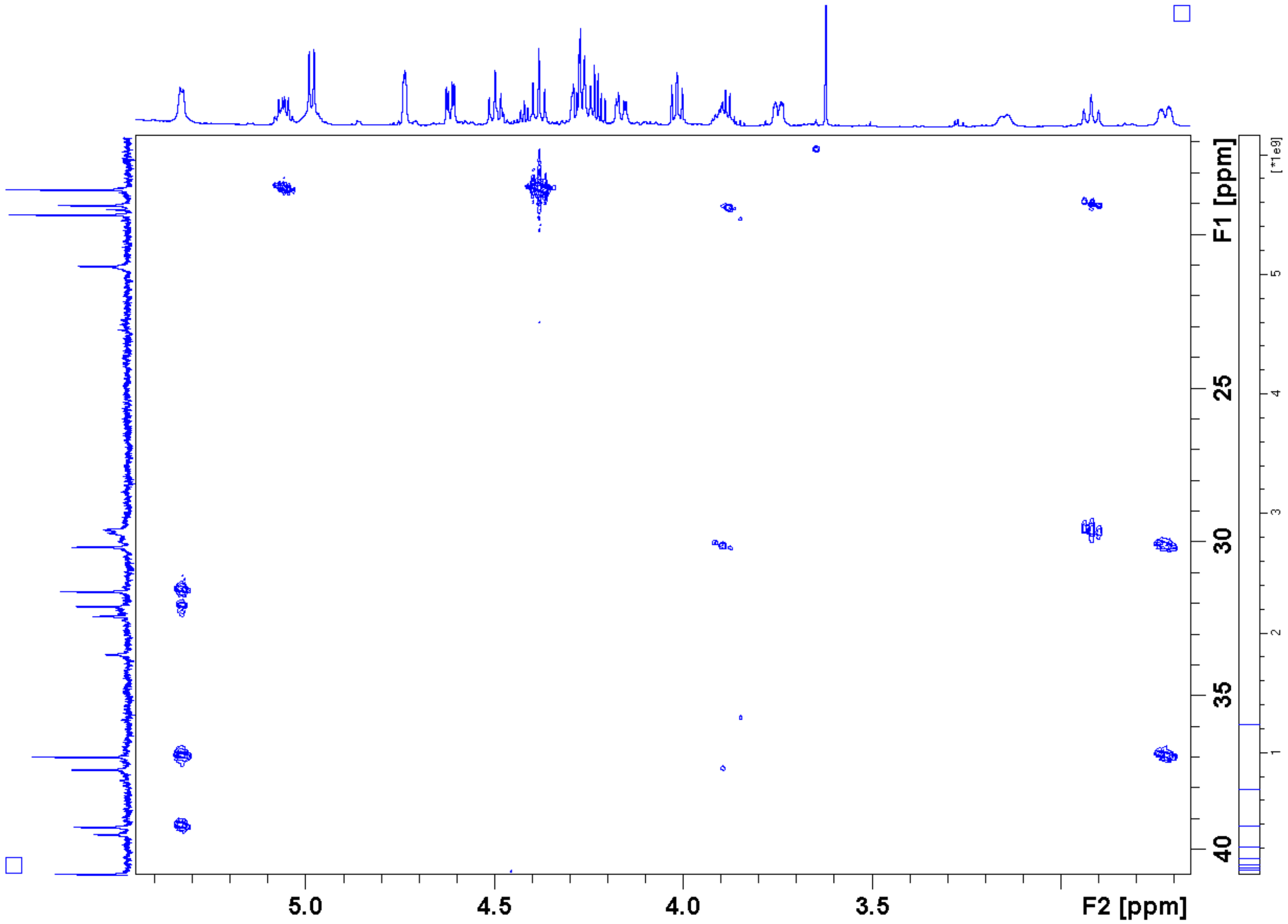

Figure S28. ^{1}H–^{13}C HMBC NMR spectrum of khasianine in pyridine-d_5 at 25 °C.

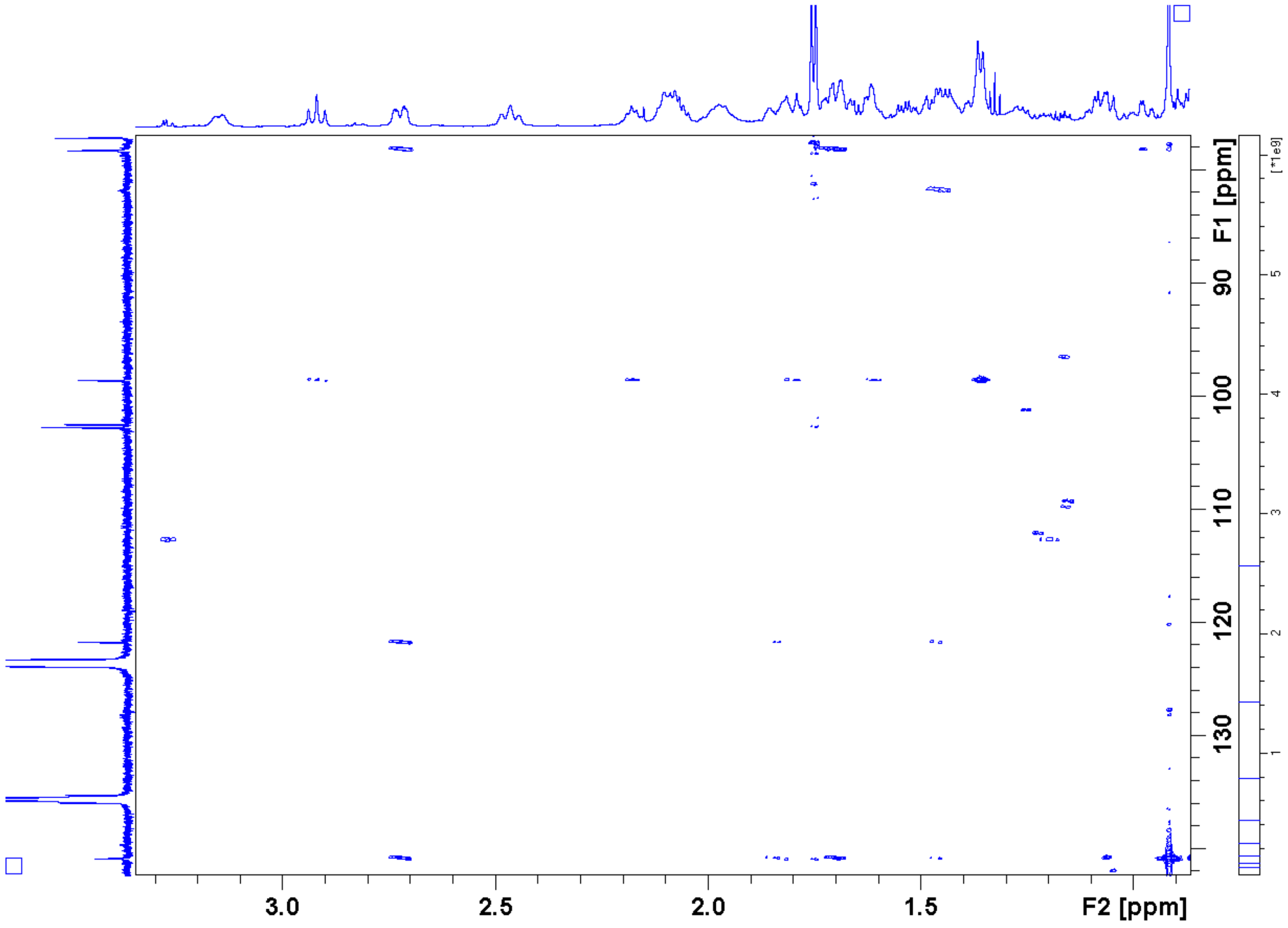

Figure S29. ^{1}H–^{13}C HMBC NMR spectrum of khasianine in pyridine-d_5 at 25 °C.

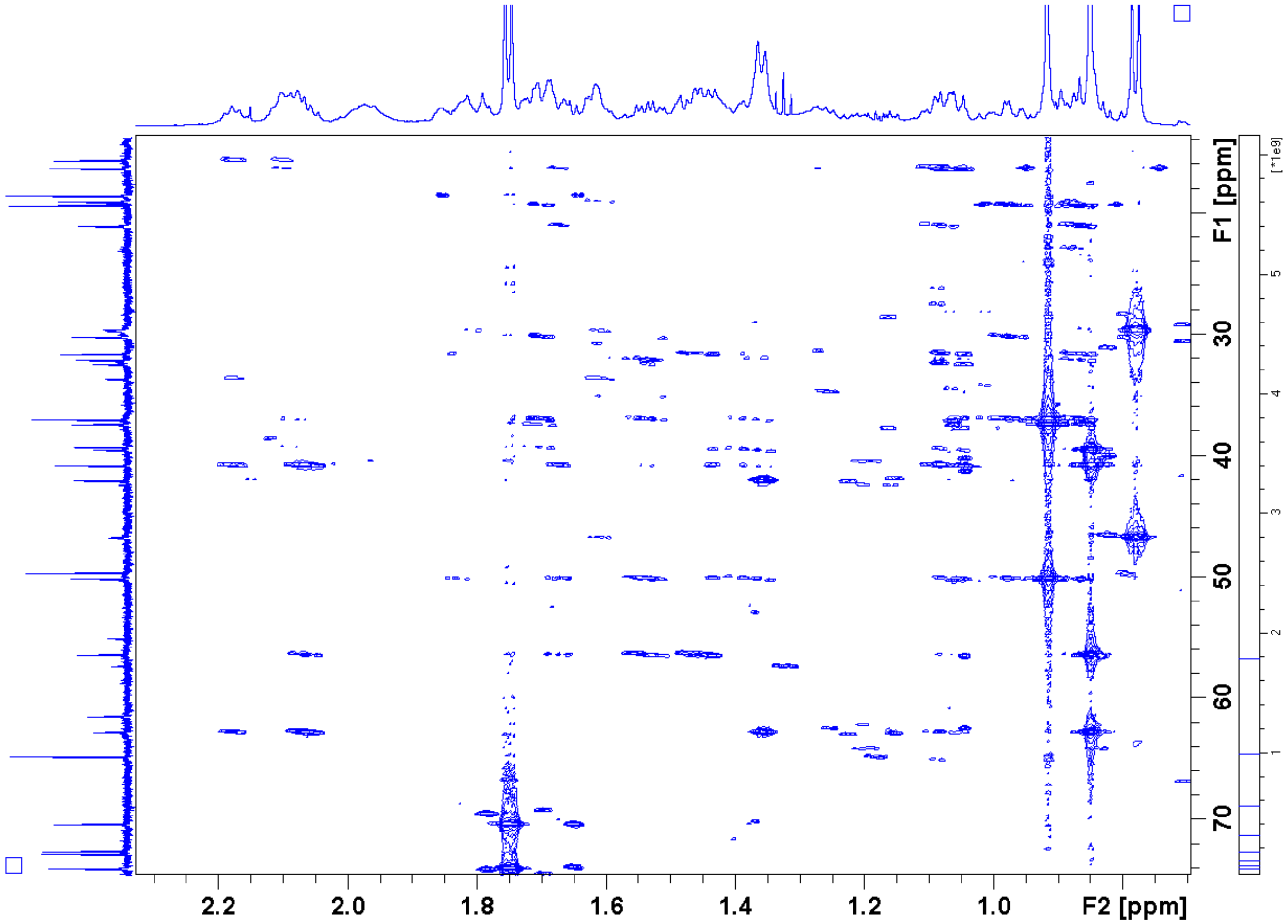

Figure S30. ^{1}H–^{13}C HMBC NMR spectrum of khasianine in pyridine-d_5 at 25 °C.

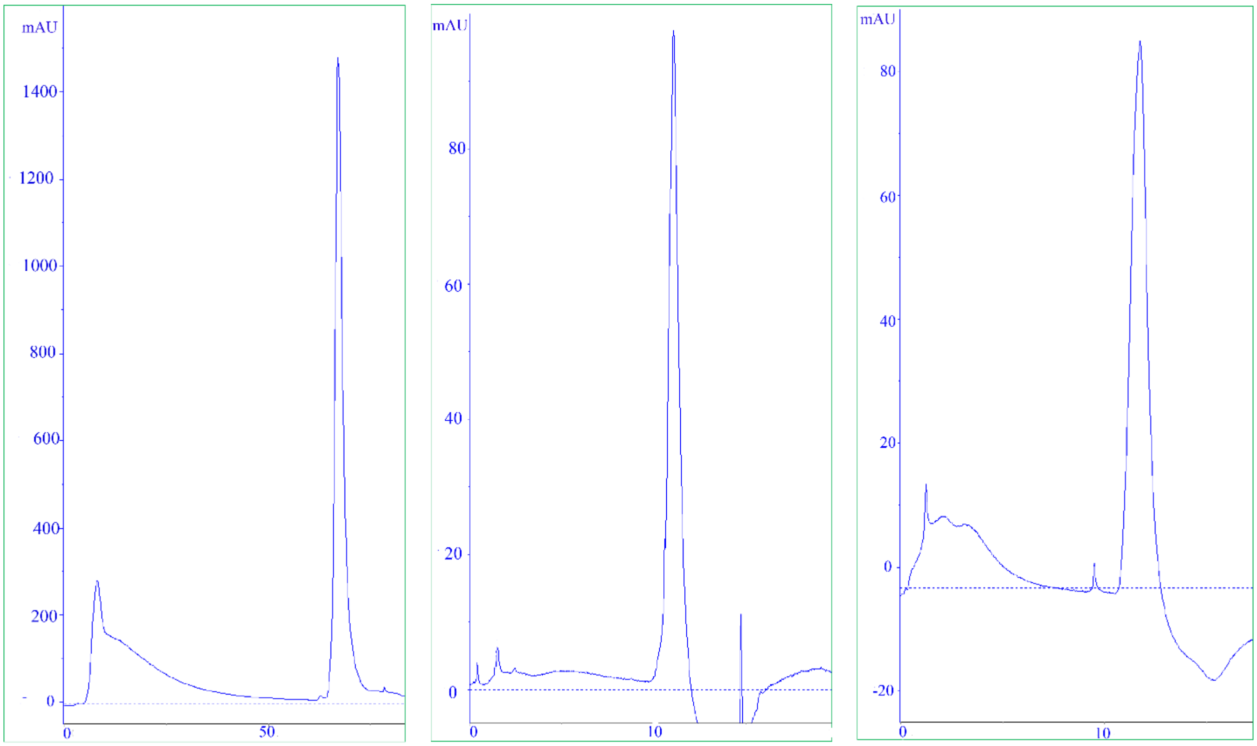

A. (Suit2-007 lysate) B. (LSBPs only) C. (LSBPs + Galactose)

Figure S31A–C. Protein isolation chromatograms

A) LSBPs were isolated from Suit2-007 cell lysates by affinity chromatography. B) Re-isolation of LSBPs by affinity chromatography with loading buffer not containing sugar. Bound LSBPs were desorbed with the elution buffer. C) Re-isolation of LSBPs by affinity chromatography with the addition of Gal to the mobile phase to compete with the LSBPs in binding to the lactosyl resin.

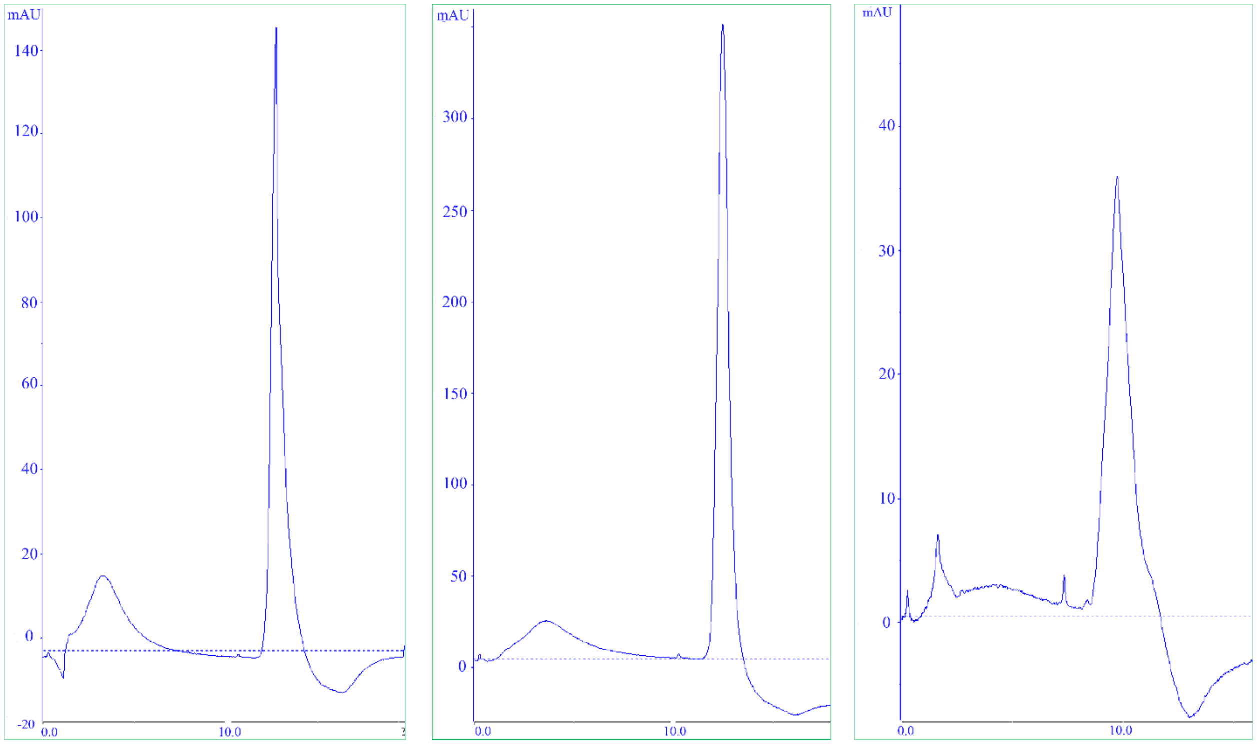

D. (LSBPs + Glucose) E. (LSBPs + Rhamnose) F. (LSBPs + Lactose)

Figure S31D–F. Protein isolation chromatograms

Re-isolation of LSBPs by affinity chromatography with the addition of the indicated sugars to the mobile phase to compete with the LSBPs in binding to the lactosyl resin.

Table S3: Expression of selected Gal-sensitive LSBPs after khasianine treatment

Gene-IDa	Corresponding name for Gal-sensitive LSBP genes[b]	LFQ ratio (protein)[c]	Expression FC (mRNA)[d]
ACAT2	Acetyl-CoA acetyltransferase 2	0.35	0.44
ACOX1	Acyl-CoA oxidase 1	0.87	1.14
AIP	Aryl hydrocarbon receptor interacting protein	0.68	1.12
AK2	Adenylate kinase 2	0.76	0.65
ARPC1B	Actin related protein 2/3 complex subunit 1B	0.88	1.29
ATXN2L	Ataxin 2 like	0.95	1.41
BAIAP2	BAR/IMD domain containing adaptor protein 2	0.42	0.89
BANF1	BAF nuclear assembly factor 1	0.59	0.50
CASK	Calcium/calmodulin dependent serine protein kinase	0.91	0.95

CAVIN1/SDPR	Caveolae associated protein 1	0.00	0.42
CD2AP	CD2 associated protein	0.93	1.03
CELF1/ CUGBP1	CUGBP Elav-like family member 1	0.41	1.10
CIAPIN1	Cytokine induced apoptosis inhibitor 1	0.87	0.70
CLTA	Clathrin light chain A	1.22	1.12
COLGALT1	Collagen beta(1-O)galactosyltransferase 1	1.04	0.98
CRYZL1	Crystallin zeta like 1	0.54	1.16
CSTF1	Cleavage stimulation factor subunit 1	1.31	0.78
CSTF3	Cleavage stimulation factor subunit 3	0.75	0.46
CTBP1	C-terminal binding protein 1	0.60	0.61
CTSA	Cathepsin A	0.44	1.70
CTSC	Cathepsin C	0.35	0.71
CYB5B	Cytochrome b5 type B	1.31	0.86
DAZAP1	DAZ associated protein 1	0.23	0.77
DBR1	Debranching RNA lariats 1	1.32	0.64
DCPS	Decapping enzyme, scavenger	1.01	0.64
DCTN3	Dynactin subunit 3	0.41	1.03
DDAH1	Dimethylarginine dimethylaminohydrolase 1	0.80	0.68
DFFA	DNA fragmentation factor subunit alpha	0.71	0.67
DHPS	Deoxyhypusine synthase	0.96	1.33
DIABLO	Diablo IAP-binding mitochondrial protein	0.64	1.20
DLG1	Discs large MAGUK scaffold protein 1	1.28	1.03
DNAJB1	DnaJ heat shock protein family (Hsp40) member B1	0.81	0.77
DRG1	Developmentally regulated GTP binding protein 1	0.73	0.82
ECH1	Enoyl-CoA hydratase 1	1.04	0.98
EIF3J	Eukaryotic translation initiation factor 3 subunit J	0.84	1.06
EIF3M	Eukaryotic translation initiation factor 3 subunit M	1.21	1.23
EIF4E	Eukaryotic translation initiation factor 4E	0.91	0.91
FKBP5	FKBP prolyl isomerase 5	1.00	0.66
FTO	FTO alpha-ketoglutarate dependent dioxygenase	0.82	0.89
HEXA	Hexosaminidase subunit alpha	0.54	1.12
IDH3A	Isocitrate dehydrogenase (NAD(+)3 catalytic subunit alpha	0.75	0.78
IFI16	Interferon gamma inducible protein 16	0.73	2.76
ISYNA1	Inositol-3-phosphate synthase 1	0.64	0.76
ITGAV	Integrin subunit alpha V	0.85	1.60

LPP	LIM domain containing preferred translocation partner in lipoma	0.67	1.85
LRPPRC	Leucine rich pentatricopeptide repeat containing	5.35	0.91
LSM3	LSM3 homolog, U6 small nuclear RNA and mRNA degradation associated	0.28	0.53
LYAR	Ly1 antibody reactive	0.58	0.46
MAN2A1	Mannosidase alpha class 2A member 1	1.02	1.58
MAT2B	Methionine adenosyltransferase 2B	0.86	0.72
MCTS1	MCTS1 re-initiation and release factor	0.38	1.02
ME1	Malic enzyme 1	0.72	1.49
METAP2	Methionyl aminopeptidase 2	1.05	0.88
MRPL48	Mitochondrial ribosomal protein L48	0.68	0.93
MRPS23	Mitochondrial ribosomal protein S23	0.51	0.68
MRRF	Mitochondrial ribosome recycling factor	0.13	1.91
NAGA	Alpha-N-acetylgalactosaminidase	0.58	0.98
NARS1	Asparaginyl-tRNA synthetase 1	1.07	1.48
NIPSNAP3A	Nipsnap homolog 3A	0.73	1.12
NIT1	Nitrilase 1	0.67	1.29
NLE1	Notchless homolog 1	0.68	0.87
NOLC1	Nucleolar and coiled-body phosphoprotein 1	2.96	0.51
NPLOC4	NPL4 homolog, ubiquitin recognition factor	1.22	1.18
NPM3	Nucleophosmin/nucleoplasmin 3	1.00	0.64
NT5C2	5'-nucleotidase, cytosolic II	1.33	1.37
NUDCD1	NudC domain containing 1	0.60	0.97
NUP214	Nucleoporin 214	1.03	1.07
NUP37	Nucleoporin 37	0.59	0.80
NUP43	Nucleoporin 43	0.41	1.04
NUP50	Nucleoporin 50	0.58	1.37
NUP98	Nucleoporin 98 and 96 precursor	0.34	0.84
OAT	Ornithine aminotransferase	0.84	0.76
OGFOD1	2-oxoglutarate and iron dependent oxygenase domain containing 1	0.39	0.92
OLA1	Obg like ATPase 1	0.77	0.95
OSBP	Oxysterol binding protein	0.00	2.13
PACSIN2	Protein kinase C and casein kinase substrate in neurons 2	1.08	1.20
PAPSS1	3'-phosphoadenosine 5'-phosphosulfate synthase 1	0.79	0.97
PBK	PDZ binding kinase	0.98	0.50

PCBD1	Pterin-4 alpha-carbinolamine dehydratase 1	0.50	0.65
PCNP	PEST proteolytic signal containing nuclear protein	0.75	1.17
PDXK	Pyridoxal kinase	0.68	0.58
PFDN5	Prefoldin subunit 5	0.31	0.86
PLAU	Plasminogen activator, urokinase	0.59	0.77
PLIN3	Perilipin 3	0.79	1.00
PPAT	Phosphoribosyl pyrophosphate amidotransferase	0.79	0.63
PPM1B	Protein phosphatase, Mg2+/Mn2+ dependent 1B	1.17	0.83
PPP1CB	Protein phosphatase 1 catalytic subunit beta	0.52	0.93
PPP1R10	Protein phosphatase 1 regulatory subunit 10	0.00	1.39
PPP2CB	Potein phosphatase 2 catalytic subunit beta	0.42	1.34
PPP4C	Protein phosphatase 4 catalytic subunit	0.17	0.88
PRDX2	Peroxiredoxin 2	0.56	0.51
PRPS2	Phosphoribosyl pyrophosphate synthetase 2	0.81	0.57
PSMB10	Proteasome 20S subunit beta 10	0.45	0.47
PSMC3	Proteasome 26S subunit, ATPase 3	1.10	0.73
PSME3	Proteasome activator subunit 3	0.72	0.43
PYCARD	PYD and CARD domain containing	0.13	0.69
RAB2A	RAB2A, member RAS oncogene family	1.25	1.17
RAVER1	Ribonucleoprotein, PTB binding 1	0.89	0.69
RBM26	RNA binding motif protein 26	0.76	0.94
RPIA	Ribose 5-phosphate isomerase A	0.90	0.59
RPL18	Ribosomal protein L18	4.31	1.00
RPL27A	Ribosomal protein L27a	3.71	1.12
RPL38	Ribosomal protein L38	1.10	0.96
RPL8	Ribosomal protein L8	2.69	0.58
RPRD1B	Regulation of nuclear pre-mRNA domain containing 1B	0.36	1.03
RPS21	Ribosomal protein S21	0.37	0.69
RPS24	Ribosomal protein S24	0.16	0.55
RPS6	Ribosomal protein S6	0.00	1.16
RPS8	Ribosomal protein S8	3.33	0.98
RRP9	Ribosomal RNA processing 9, U3 small nucleolar RNA binding protein	1.20	0.60
S100A13	S100 calcium binding protein A13	0.15	1.76
SAE1	SUMO1 activating enzyme subunit 1	0.67	0.78
SETD3	SET domain containing 3, actin histidine	0.80	0.76

	methyltransferase		
SLC4A1AP	Solute carrier family 4 member 1 adaptor protein	1.32	1.21
SMS	Spermine synthase	0.78	1.28
SRM	Spermidine synthase	1.35	0.44
SRRM2	Serine/arginine repetitive matrix 2	3.32	0.90
SRSF11	Serine and arginine rich splicing factor 11	2.98	1.03
SRSF7	Serine and arginine rich splicing factor 7	0.00	0.63
SUPT16H	SPT16 homolog, facilitates chromatin remodeling subunit	3.51	0.59
SUSD2	Sushi domain containing 2	0.61	0.84
SYAP1	Synapse associated protein 1	0.88	1.17
THUMPD1	THUMP domain containing 1	0.69	1.10
TP53I3	Tumor protein p53 inducible protein 3	0.70	1.38
TPD52L2	TPD52 like 2	0.66	1.66
TRA2B	Transformer 2 beta homolog	5.36	0.62
TRIM28	Tripartite motif containing 28	1.31	0.77
TSG101	Tumor susceptibility 101	0.89	1.34
TTLL12	Tubulin tyrosine ligase like 12	1.43	0.51
TXNDC12	Thioredoxin domain containing 12	0.40	1.82
TYMS	Thymidylate synthetase	0.00	0.35
UBE2K	Ubiquitin conjugating enzyme E2 K	1.05	1.25
UBFD1	Ubiquitin family domain containing 1	0.46	1.24
WARS1	Tryptophanyl-tRNA synthetase 1	0.00	5.24
WDR12	WD repeat domain 12	0.60	0.58
WDR4	WD repeat domain 4	0.67	0.46
XRCC4	X-ray repair cross complementing 4	1.00	1.02
YARS1	Tyrosyl-tRNA synthetase 1	0.00	1.47

[a,b]Gene ID and corresponding names for selected Gal -sensitive LSBP genes.
[c]Label free quantification ratio of Gal-sensitive LSBPs comparing treated versus control samples.
[d]Fold expression fold change of Gal -sensitive LSBP genes comparing treated versus control samples.

Table S4: Expression of selected Glc-sensitive LSBPs after khasianine treatment

Gene-ID[a]	Corresponding name for Glc-sensitive LSBP genes[b]	LFQ ratio (protein)[c]	Expression FC (mRNA)[d]
ACO2	Aconitase 2	1.17	1.27
AIP	Aryl hydrocarbon receptor interacting protein	0.68	1.12
AKR1A1	Aldo-keto reductase family 1 member A1	0.53	2.03
ARL3	ADP ribosylation factor like GTPase 3	0.00	0.97
BUD23	BUD23 rRNA methyltransferase and ribosome maturation factor	0.00	0.55
CHMP2A	Charged multivesicular body protein 2A	0.50	1.39
CLIP2	CAP-Gly domain containing linker protein 2	0.99	1.12
COLGALT1	Collagen beta(1-O)galactosyltransferase 1	1.04	0.98
COPS4	COP9 signalosome subunit 4	1.26	0.83
COPS6	COP9 signalosome subunit 6	0.61	0.52
COPS7A	COP9 signalosome subunit 7A	0.75	0.98
CTSA	Cathepsin A	0.44	1.70
DHX9	DExH-box helicase 9	13.13	0.45
DNAJB11	DnaJ heat shock protein family (Hsp40) member B11	1.22	1.30
DSTN	Destrin, actin depolymerizing factor	0.96	1.15
EMG1	EMG1 N1-specific pseudouridine methyltransferase	0.48	1.12
ESD	Esterase D	1.13	0.87
FHL2	Four and a half LIM domains 2	0.79	1.94
GATD1	Glutamine amidotransferase like class 1 domain containing 1	0.00	0.75
HGS	Hepatocyte growth factor-regulated tyrosine kinase substrate	0.80	1.46
HIF1AN	Hypoxia inducible factor 1 subunit alpha inhibitor	0.76	1.34
IFI35	Interferon induced protein 35	0.52	1.14
KHSRP	KH-type splicing regulatory protein	1.09	0.72
LACTB2	Lactamase beta 2	0.44	1.13
LAMTOR3	Late endosomal/lysosomal adaptor, MAPK and MTOR Activator 3	0.00	1.73
LEO1	LEO1 homolog, Paf1/RNA polymerase II complex component	0.00	0.87
LGMN	Legumain	0.31	0.91
LPP	LIM domain containing preferred translocation partner in lipoma	0.67	1.85
LRPPRC	Leucine rich pentatricopeptide repeat containing	5.35	0.91
MAPK1	Mitogen-activated protein kinase 1	1.32	1.35

MCCC2	Methylcrotonoyl-CoA carboxylase 2	1.21	0.56
MINPP1	Multiple inositol-polyphosphate phosphatase 1	0.67	1.01
MRPL48	Mitochondrial ribosomal protein L48	0.68	0.93
MRRF	Mitochondrial ribosome recycling factor	0.13	1.91
MTHFD2	Methylenetetrahydrofolate dehydrogenase (NADP+ dependent) 2, methenyltetrahydrofolate cyclohydrolase	0.85	2.52
NANS	N-acetylneuraminate synthase	0.75	1.15
NAPA	NSF attachment protein alpha	0.93	0.97
NARS1	Asparaginyl-tRNA synthetase 1	0.00	1.48
NDUFS1	NADH:ubiquinone oxidoreductase core subunit S1	0.66	1.00
NDUFV2	NADH:ubiquinone oxidoreductase core subunit V2	0.52	0.84
NIT1	Nitrilase 1	0.67	1.29
NPLOC4	NPL4 homolog, ubiquitin recognition factor	1.22	1.18
NT5C2	5'-nucleotidase, cytosolic II	1.33	1.37
OGFOD1	2-oxoglutarate and iron dependent oxygenase domain containing 1	0.39	0.92
PAPSS1	3'-phosphoadenosine 5'-phosphosulfate synthase 1	0.79	0.97
PARK7	Parkinsonism associated deglycase	0.51	0.89
PDCD10	Programmed cell death 10	0.36	1.89
PEBP1	Phosphatidylethanolamine binding protein 1	0.82	0.57
PHB	Prohibitin	3.16	0.30
PITRM1	Pitrilysin metallopeptidase 1	0.83	1.40
PLIN3	Perilipin 3	0.79	1.00
PLS1	Plastin 1	1.09	0.73
PPP1R10	Protein phosphatase 1 regulatory subunit 10	0.00	1.39
PPP1R7	Protein phosphatase 1 regulatory subunit 7	1.02	0.82
PPP2CB	Protein phosphatase 2 catalytic subunit beta	0.42	1.34
PPP4C	Protein phosphatase 4 catalytic subunit	0.17	0.88
PRCP	Prolylcarboxypeptidase	0.40	0.89
PRMT5	Protein arginine methyltransferase 5	1.00	0.61
PRPS2	Phosphoribosyl pyrophosphate synthetase 2	0.81	0.57
PSMB10	Proteasome 20S subunit beta 10	0.46	0.47
PSMB9	Proteasome 20S subunit beta 9	0.77	0.51
PSMC3	Proteasome 26S subunit, ATPase 3	1.10	0.73
PSMG2	Proteasome assembly chaperone 2	0.71	1.14
PTPN11	Protein tyrosine phosphatase non-receptor type 11	0.89	1.00
RAB2A	RAB2A, member RAS oncogene family	1.25	1.17

RAVER1	Ribonucleoprotein, PTB binding 1	0.89	0.69
RBM22	RNA binding motif protein 22	0.38	1.33
RCC1	Regulator of chromosome condensation 1	1.13	0.50
RELA	RELA proto-oncogene, NF-kB subunit	0.89	2.05
RPA2	Replication protein A2	0.51	0.70
RPL27	Ribosomal protein L27	3.98	1.03
RPL38	Ribosomal protein L38	1.09	0.96
RPL8	Ribosomal protein L8	2.69	0.58
RUVBL2	RuvB like AAA ATPase 2	1.29	0.49
SDHA	Succinate dehydrogenase complex flavoprotein subunit A	0.90	0.76
SH3GL1	SH3 domain containing GRB2 like 1, endophilin A2	0.45	0.85
SH3KBP1	SH3 domain containing kinase binding protein 1	0.00	1.17
SMU1	SMU1 DNA replication regulator and spliceosomal factor	0.57	0.93
SNRNP70	Small nuclear ribonucleoprotein U1 subunit 70	6.83	0.43
SNX5	Sorting nexin 5	0.45	0.73
TIPRL	TOR signaling pathway regulator	0.58	1.30
TMOD3	Tropomodulin 3	0.69	1.15
TPD52	Tumor protein D52	0.66	0.75
UMPS	Uridine monophosphate synthetase	1.00	0.77
UROD	Uroporphyrinogen decarboxylase	0.42	0.66
WDR4	WD repeat domain 4	0.67	0.46
WDR77	WD repeat domain 77	1.22	0.42
YARS1	tyrosyl-tRNA synthetase 1	0.00	1.47

[a,b]Gene ID and corresponding names for selected Glc-sensitive LSBP genes.
[c]Label free quantification ratio of Glc-sensitive LSBPs comparing treated versus control samples.
[d]Fold expression fold change of Glc-sensitive LSBP genes comparing treated versus control samples.

Table S5: Expression of selected Lac-sensitive LSBPs after khasianine treatment

Gene-ID[a]	Corresponding name for Lac-sensitive LSBP genes[b]	LFQ ratio (protein)[c]	Expression FC(mRNA)[d]
AARS1	Alanyl-tRNA synthetase 1	0.00	2.10
ACAT2	Acetyl-CoA acetyltransferase 2	0.35	0.44
AHNAK	AHNAK nucleoprotein	1.15	1.90
ALDOC	Aldolase, fructose-bisphosphate C	0.68	1.79
APEX1	Apurinic/apyrimidinic endodeoxyribonuclease 1	0.45	0.60
APLP2	Amyloid beta precursor like protein 2	0.00	2.08
ARHGDIB	Rho GDP dissociation inhibitor beta	0.41	0.36
ARRB1	Arrestin beta 1	0.98	0.32
BANF1	BAF nuclear assembly factor 1	0.59	0.50
C1QBP	Complement C1q binding protein	0.29	0.62
CACYBP	Calcyclin binding protein	0.42	0.63
CAPG	Capping actin protein, gelsolin like	0.41	1.59
CAT	Catalase	0.75	0.68
CCT2	Chaperonin containing TCP1 subunit 2	0.91	0.61
CLIP1	CAP-Gly domain containing linker protein 1	0.96	1.62
CSTB	Cystatin B	0.67	1.53
CTSD	Cathepsin D	0.55	1.66
DCTN2	Dynactin subunit 2	0.41	1.50
DCTPP1	dCTP pyrophosphatase 1	0.00	0.27
DDB1	Damage specific DNA binding protein 1	3.45	0.68
DFFA	DNA fragmentation factor subunit alpha	0.71	0.67
DLAT	Dihydrolipoamide S-acetyltransferase	0.76	0.66
DUT	Deoxyuridine triphosphatase	0.38	0.31
EFHD2	EF-hand domain family member D2	0.50	0.64
ERO1A	Endoplasmic reticulum oxidoreductase 1 alpha	0.00	1.72
FKBP4	FKBP prolyl isomerase 4	0.72	0.60
FTH1	Ferritin heavy chain 1	0.70	4.33
G3BP1	G3BP stress granule assembly factor 1	1.13	0.48
GGCT	Gamma-glutamylcyclotransferase	0.35	0.63
GGH	Gamma-glutamyl hydrolase	0.74	0.58
GNPDA1	Glucosamine-6-phosphate deaminase 1	0.66	1.76
GNS	Glucosamine (N-acetyl)-6-sulfatase	0.87	1.53
HDGF	Heparin binding growth factor	0.79	0.58

HMGA1	High mobility group AT-hook 1	20.40	2.48
HMGB3	High mobility group box 3	0.76	0.68
HNRNPAB	Heterogeneous nuclear ribonucleoprotein A/B	0.58	0.37
HNRNPD	Heterogeneous nuclear ribonucleoprotein D	0.62	0.64
HNRNPDL	Heterogeneous nuclear ribonucleoprotein D like	1.10	1.73
HNRNPM	Heterogeneous nuclear ribonucleoprotein M	0.96	0.63
HNRNPR	Heterogeneous nuclear ribonucleoprotein R	1.00	0.61
HSPA5	Heat shock protein family A (Hsp70) member 5	0.90	4.02
HSPB1	Heat shock protein family B (small) member 1	0.48	0.63
HSPD1	Heat shock protein family D (Hsp60) member 1	1.34	0.58
HSPE1	Heat shock protein family E (Hsp10) member 1	0.40	0.50
HSPH1	Heat shock protein family H (Hsp110) member 1	1.20	0.64
HYOU1	Hypoxia up-regulated 1	1.08	2.97
IDH1	Isocitrate dehydrogenase (NADP(+)) 1	0.79	1.92
IGFBP7	Insulin like growth factor binding protein 7	0.43	0.53
ILF3	Interleukin enhancer binding factor 3	0.88	0.36
KRT18	Keratin 18	0.79	0.42
LGALS1	Galectin 1	0.49	1.45
LMNA	Lamin A/C	0.32	0.52
LMNB1	Lamin B1	0.97	0.26
LMNB2	Lamin B2	0.91	0.44
LSM3	LSM3 homolog, U6 small nuclear RNA and mRNA degradation associated	0.28	0.53
MANF	Mesencephalic astrocyte derived neurotrophic factor	0.30	1.70
MARCKS	Myristoylated alanine rich protein kinase C substrate	0.39	1.45
MATR3	Matrin 3	3.41	0.66
MRPL12	Mitochondrial ribosomal protein L12	0.42	0.66
MSN	Moesin	0.53	1.58
NCL	Nucleolin	1.13	0.59
NUDC	Nuclear distribution C, dynein complex regulator	0.66	0.48
P4HB	Prolyl 4-hydroxylase subunit beta	1.00	1.74
PA2G4	Proliferation-associated 2G4	0.58	0.56
PAICS	Phosphoribosylaminoimidazole carboxylase and phosphoribosylaminoimidazolesuccinocarboxamide synthase	0.64	0.55
PCNA	Proliferating cell nuclear antigen	0.95	0.32
PDXK	Pyridoxal kinase	0.68	0.58

PFDN2	Prefoldin subunit 2	0.53	1.99
PGAM1	Phosphoglycerate mutase 1	0.70	0.42
PGLS	6-phosphogluconolactonase	0.94	2.01
PLRG1	Pleiotropic regulator 1	0.40	1.64
PPIA	Peptidylprolyl isomerase A	0.78	0.57
PRDX2	Peroxiredoxin 2	0.56	0.51
PRDX3	Peroxiredoxin 3	0.43	0.60
PRDX4	Peroxiredoxin 4	1.00	0.63
PRMT1	Protein arginine methyltransferase 1	0.78	0.63
PSAP	Prosaposin	0.71	2.12
PSAT1	Phosphoserine aminotransferase 1	0.59	1.97
PSMA2	Proteasome 20S subunit alpha 2	0.94	0.62
PSMA3	Proteasome 20S subunit alpha 3	0.88	0.68
PSMB3	Proteasome 20S subunit beta 3	0.72	0.57
PTBP1	Polypyrimidine tract binding protein 1	0.42	0.63
PUF60	Poly(U) binding splicing factor 60	0.86	0.60
RANBP1	RAN binding protein 1	0.67	0.37
RPL6	Ribosomal protein L6	8.93	0.54
RPS7	Ribosomal protein S7	1.17	0.68
RUVBL1	RuvB like AAA ATPase 1	0.73	0.49
SERBP1	SERPINE1 mRNA binding protein 1	1.05	0.67
SERPINB1	Serpin family B member 1	0.81	1.77
SERPINB8	Serpin family B member 8	0.52	5.54
SFN	Stratifin	0.23	2.51
SLC3A2	Solute carrier family 3 member 2	1.11	2.29
SNRPD1	Small nuclear ribonucleoprotein D1 polypeptide	1.11	0.63
SNRPD3	Small nuclear ribonucleoprotein D3 polypeptide	0.77	0.48
SOD1	Superoxide dismutase 1	0.44	0.68
SPTAN1	Spectrin alpha, non-erythrocytic 1	0.88	1.72
SRSF1	Serine and arginine rich splicing factor 1	2.98	0.68
STMN1	Stathmin 1	0.63	0.40
SYNCRIP	Synaptotagmin binding cytoplasmic RNA interacting protein	0.82	0.40
TCEA1	Transcription elongation factor A1	0.53	2.02
TCERG1	Transcription elongation regulator 1	0.75	0.61
TFG	Trafficking from ER to golgi regulator	0.61	1.83
TPI1	Triosephosphate isomerase 1	0.74	0.66

TPM1	Tropomyosin 1	0.58	0.46
TPM4	Tropomyosin 4	0.70	1.69
TPP1	Tripeptidyl peptidase 1	1.08	3.27
TXNDC12	Thioredoxin domain containing 12	0.40	1.82
TXNRD1	Thioredoxin reductase 1	0.77	2.89
VCL	Vinculin	0.93	0.68
WARS1	Tryptophanyl-tRNA synthetase 1	0.00	5.24
YWHAE	Tyrosine 3-monooxygenase/tryptophan 5-monooxygenase activation protein epsilon	0.78	0.60

[a,b]Gene ID and corresponding names for selected Lac-sensitive LSBP genes.
[c]Label free quantification ratio of Lac-sensitive LSBPs comparing treated versus control samples.
[d]Fold expression fold change of Lac-sensitive LSBP genes comparing treated versus control samples.

Table S6: Expression of Rha-sensitive LSBPs associated with three functional annotations

**Gene-ID[a]**	**Rha-sensitive LSBPs associated with cell movement[b]**	**LFQ ratio (protein)[c]**	**Expression FC (mRNA)[d]**
ACTB	Actin beta	0.99	0.94
ACTN4	Actinin alpha 4	0.89	0.72
AHNAK	AHNAK nucleoprotein	1.15	1.90
AKR1B1	Aldo-keto reductase family 1 member B	0.32	0.93
ALDH2	Aldehyde dehydrogenase 2 family member	1.20	1.14
ALDOA	Aldolase, fructose-bisphosphate A	0.90	1.10
ANXA1	Annexin A1	0.60	1.28
ARHGDIA	Rho GDP dissociation inhibitor alpha	0.86	1.41
ARHGDIB	Rho GDP dissociation inhibitor beta	0.40	0.36
ATP5F1A	ATP synthase F1 subunit alpha	0.00	0.74
ATP5F1B	ATP synthase F1 subunit beta	0.00	0.84
C1QBP	Complement C1q binding protein	0.29	0.62
CALR	Calreticulin	0.91	1.03
CAP1	Cyclase associated actin cytoskeleton regulatory protein 1	0.64	1.40
CAT	Catalase	0.75	0.68
CFL1	Cofilin 1	0.59	0.75
CLTC	Clathrin heavy chain	1.94	1.05
DPYSL2	Eihydropyrimidinase like 2	0.87	0.76
ELAVL1	ELAV like RNA binding protein 1	0.67	0.92
ENO1	Enolase 1	1.37	0.97

EZR	Ezrin	0.52	0.78
FLNA	Filamin A	1.77	1.66
FLNB	Filamin B	2.02	3.27
FTH1	Ferritin heavy chain 1	0.70	4.33
GAPDH	Glyceraldehyde-3-phosphate dehydrogenase	0.76	1.24
GDI1	GDP dissociation inhibitor 1	0.00	2.4
GTF2I	General transcription factor IIi	0.99	0.59
HDGF	Heparin binding growth factor	0.79	0.58
HMGA1	High mobility group AT-hook 1	20.40	2.48
HMGB2	High mobility group box 2	1.00	1.08
HNRNPA2B1	Heterogeneous nuclear ribonucleoprotein A2/B1	0.27	1.41
HNRNPAB	Heterogeneous nuclear ribonucleoprotein A/B	0.58	0.37
HNRNPK	Heterogeneous nuclear ribonucleoprotein K	1.71	0.93
HSP90AA1	Heat shock protein 90 alpha family class A member 1	1.62	0.41
HSP90AB1	Heat shock protein 90 alpha family class B member 1	2.16	0.71
HSP90B1	Heat shock protein 90 beta family member 1	2.17	1.20
HSPA5	Heat shock protein family A (Hsp70) member 5	0.90	4.02
HSPA9	Heat shock protein family A (Hsp70) member 9	0.79	1.4
HSPB1	Heat shock protein family B (small) member 1	0.48	0.63
HSPD1	Heat shock protein family D (Hsp60) member 1	1.34	0.58
IDH1	Isocitrate dehydrogenase (NADP(+)) 1	0.79	1.92
KRT18	Keratin 18	0.79	0.42
LDHA	Lactate dehydrogenase A	5.16	0.81
LMNA	Lamin A/C	0.32	0.52
LMNB1	Lamin B1	0.97	0.26
MCM3	Minichromosome maintenance complex component 3	1.82	0.24
MSN	Moesin	0.53	1.58
MYH9	Mosin heavy chain 9	1.88	1.45
NACA	Nascent polypeptide associated complex subunit alpha	1.73	0.98
NCL	Nucleolin	1.13	0.59
NPM1	Nucleophosmin 1	1.04	1.04
P4HB	Prolyl 4-hydroxylase subunit beta	1.00	1.74
PARP1	Poly(ADP-ribose) polymerase 1	1.38	0.46
PDIA3	Protein disulfide isomerase family A member 3	1.07	0.97
PEBP1	Phosphatidylethanolamine binding protein 1	0.82	0.57
PKM	Pyruvate kinase M1/2	1.11	0.76

PLEC	Plectin	1.56	1.75
PPIA	Peptidylprolyl isomerase A	0.78	0.57
PPIB	Peptidylprolyl isomerase B	0.31	1.10
PRDX1	Peroxiredoxin 1	0.74	1.09
PRDX2	Peroxiredoxin 2	0.56	0.51
PRDX6	Peroxiredoxin 6	0.66	0.83
RUVBL1	RuvB like AAA ATPase 1	0.73	0.49
S100A8	S100 calcium binding protein A8	0.00	0.89
SFN	Stratifin	0.23	2.51
SOD1	Superoxide dismutase 1	0.44	0.68
STMN1	Stathmin 1	0.63	0.40
TKT	Transketolase	1.35	1.06
TPI1	Triosephosphate isomerase 1	0.74	0.65
TPM1	Tropomyosin 1	0.58	0.46
TXN	Thioredoxin	0.65	1.08
TXNRD1	Thioredoxin reductase 1	0.77	2.89
VCL	Vinculin	0.93	0.68
VCP	Valosin containing protein	2.67	0.81
VDAC1	Voltage dependent anion channel 1	1.38	0.70
VIM	Vimentin	1.20	1.22
YBX1	Y-box binding protein 1	0.93	0.93
YWHAE	Tyrosine 3-monooxygenase/tryptophan 5-monooxygenase activation protein epsilon	0.78	0.60
YWHAZ	Tyrosine 3-monooxygenase/tryptophan 5-monooxygenase activation protein zeta	0.88	1.18
**Gene-IDs**	**Rha-sensitive LSBPs associated with cell-cycle**		
ACTB	Actin beta	0.99	0.94
AHNAK	AHNAK nucleoprotein	1.15	1.90
C1QBP	Complement C1q binding protein	0.29	0.62
CALR	Calreticulin	0.91	1.03
CBX3	Chromobox 3	1.11	0.71
CCT4	Chaperonin containing TCP1 subunit 4	0.76	0.85
CLTC	Clathrin heavy chain	1.94	1.05
CSTB	Cystatin B	0.67	1.53
EIF6	Eukaryotic translation initiation factor 6	0.72	0.81
EZR	Ezrin	0.52	0.78
FLNA	Filamin A	1.77	1.66

HNRNPU	Heterogeneous nuclear ribonucleoprotein U	4.31	0.56
HSPA9	Heat shock protein family A (Hsp70) member 9	0.79	1.40
HSPB1	Heat shock protein family B (small) member 1	0.48	0.63
KRT18	Keratin 18	0.79	0.42
LMNA	Lamin A/C	0.32	0.52
NPM1	Nucleophosmin 1	1.40	1.04
PARP1	Poly(ADP-ribose) polymerase 1	1.38	0.46
PEBP1	Phosphatidylethanolamine binding protein 1	0.82	0.57
PRDX1	Peroxiredoxin 1	0.74	1.09
RBBP4	RB binding protein 4, chromatin remodeling factor	0.62	0.51
RPL11	Ribosomal protein L11	1.85	1.18
RPS3	Ribosomal protein S3	1.66	1.02
RUVBL1	RuvB like AAA ATPase 1	0.73	0.49
SFN	Stratifin	0.00	2.51
STMN1	Stathmin 1	0.63	0.40
TXN	Thioredoxin	0.52	1.08
VCP	Valosin containing protein	2.67	0.81
YWHAB	Tyrosine 3-monooxygenase/tryptophan 5-monooxygenase activation protein beta	0.70	0.83
YWHAE	Tyrosine 3-monooxygenase/tryptophan 5-monooxygenase activation protein epsilon	0.78	0.60
**Gene-IDs**	**Rha-sensitive LSBPs associated with cell signaling**		
ACTN4	Actinin alpha 4	0.89	0.72
ANXA1	Annexin A1	1.60	1.28
C1QBP	Complement C1q binding protein	0.29	0.62
ERP29	Endoplasmic reticulum protein 29	0.60	1.26
EZR	Ezrin	0.52	0.78
FLNA	Filamin A	1.77	1.66
HSP90B1	Heat shock protein 90 beta family member 1	2.17	1.20
HSPA5	Heat shock protein family A (Hsp70) member 5	0.90	4.02
MYH9	Myosin heavy chain 9	1.88	1.45
NCL	Nucleolin	1.13	0.59
PKM	Pyruvate kinase M1/2	1.11	0.76
RPSA	Ribosomal protein SA	1.10	0.95

[a,b]Gene ID and corresponding names for Rha-sensitive LSBP genes for three functional annotations.
[c]Label free quantification ratio of Rha-sensitive LSBPs comparing treated versus control samples.
[d]Fold expression fold change of Rha-sensitive LSBP genes comparing treated versus control samples.

Table S7: Sugar-sensitive LSBPs modulated in BDX-ASML rat model of liver colonization

Sugar sensitive LSBPs[a]	↓ Rat model[b]	↑ Rat model[c]
Gal-sensitive (n=472)	254	20
Glc-sensitive (n=209)	133	20
Lac-sensitive (n=364)	113	18
Rha-sensitive (n=151)	52	20

[a]Number of LSBPs detected in fractions eluted by different sugars.
[b]Genes downregulated (↓) in the ASML cells re-isolated from rat liver.
[c]Genes upregulated (↑)in the ASML cells re-isolated from rat liver.
